# Effects of the AMPA Antagonist ZK 200775 on Visual Function: A Randomized Controlled Trial

**DOI:** 10.1371/journal.pone.0012111

**Published:** 2010-08-12

**Authors:** Richard Bergholz, Thomas Staks, Klaus Rüther

**Affiliations:** 1 Department of Ophthalmology, Charité Universitätsmedizin Berlin, Campus Virchow-Klinikum, Berlin, Germany; 2 Bayer Schering Pharma AG, Berlin, Germany; National Institutes of Health, United States of America

## Abstract

**Background:**

ZK 200775 is an antagonist at the α-Amino-3-hydroxy-5-methyl-4-isoxazolepropionate (AMPA) receptor and had earned attention as a possible neuroprotective agent in cerebral ischemia. Probands receiving the agent within phase I trials reported on an alteration of visual perception. In this trial, the effects of ZK 200775 on the visual system were analyzed in detail.

**Methodology:**

In a randomised controlled trial we examined eyes and vision before and after the intravenous administration of two different doses of ZK 200775 and placebo. There were 3 groups of 6 probands each: Group 1 recieved 0.03 mg/kg/h, group 2 0.75 mg/kg/h of ZK 200775, the control group received 0.9% sodium chloride solution. Probands were healthy males aged between 57 and 69 years. The following methods were applied: clinical examination, visual acuity, ophthalmoscopy, colour vision, rod absolute threshold, central visual field, pattern-reversal visual evoked potentials (pVEP), ON-OFF and full-field electroretinogram (ERG).

**Principal Findings:**

No effect of ZK 200775 was seen on eye position or motility, stereopsis, pupillary function or central visual field testing. Visual acuity and dark vision deteriorated significantly in both treated groups. Color vision was most remarkably impaired. The dark-adapted ERG revealed a reduction of oscillatory potentials (OP) and partly of the a- and b-wave, furthermore an alteration of b-wave morphology and an insignificantly elevated b/a-ratio. Cone-ERG modalities showed decreased amplitudes and delayed implicit times. In the ON-OFF ERG the ON-answer amplitudes increased whereas the peak times of the OFF-answer were reduced. The pattern VEP exhibited lower amplitudes and prolonged peak times.

**Conclusions:**

The AMPA receptor blockade led to a strong impairment of typical OFF-pathway functions like color vision and the cone ERG. On the other hand the ON-pathway as measured by dark vision and the scotopic ERG was affected as well. This further elucidates the interdependence of both pathways.

**Trial Registration:**

ClinicalTrials.gov NCT00999284

## Introduction

Glutamate is an important excitatory neurotransmitter of the retina [1], [2], [3]. Besides neuronal excitation it also participates in neuronal development, synaptic plasticity and neurotoxicity (excitoxicity) [4], [5], [6], [7]. Its activity is mediated by metabotropic and ionotropic glutamate receptors (mGluRs and iGluRs). The latter are subdivided into N-methyl-D-aspartate (NMDA) and non-NMDA receptors. The latter again are classified as α-Amino-3-hydroxy-5-methyl-4-isoxazolepropionate (AMPA, composed of the subunits iGluR1-4) and kainate (composed of the subunits iGluR6-7) receptors (see Figure S1).

Non-NMDA receptors predominate in the retinal OFF-pathway of the mammalian retina. In animal experiments, AMPA receptor subunits have been localized in the inner and outer plexiforme layer, in amacrine, bipolar and horizontal cells as well as in ganglion cells and Muller glial cells [8], [9], [10], [11]. Webvision gives a survey of published data in different species (see Table S1) [12].

ZK 200775 was developed by Schering AG (Berlin, Germany) as an antagonist at the AMPA receptor. It raised expectations as a possible neuroprotective agent in cerebral ischemia. The data presented here was obtained in the resarch phase I to evaluate the safety, tolerability and pharmacokinetics of the drug [13], [14], [15]. In earlier trial, probands receiving ZK 200775 claimed to have blurred vision and a strongly impaired color perception so the aim of this investigation was to quantifiy the ophthalmologic effects of ZK200775 by adequate examinations.

In the meanwhile, further development of the drug has been aborted because of intolerable, but vision unrelated adverse reactions [16], [17], [18].

On a microscopic level, distribution and physiology of retinal AMPA receptors have been studied exhaustively. The advent of a phase I trial of ZK200775 opened the unique opportunity to investigate the effects of a receptor blockade in vivo, i.e. to elucidate the function of AMPA receptors on visual perception in humans.

## Results

The administration of ZK 200775 had no effect on eye position (Table S2) and motility (Table S3), stereopsis as tested with the Titmus test, pupillary afference and efference (Table S4), central visual field testing with the Amsler grid (Table S5) and anterior and posterior segment morphology.

### Visual acuity

**Figure 1:**
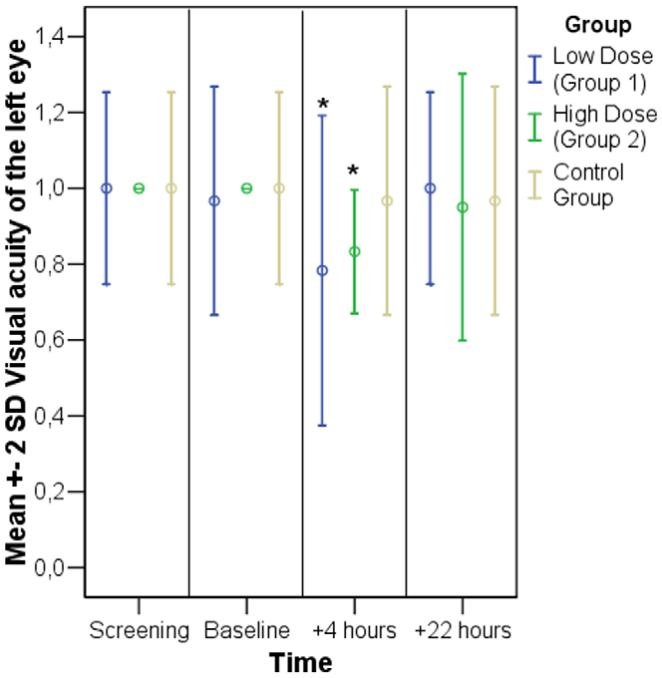
Visual acuity. Time course of mean values +/− 2 * standard deviation for 6 subjects of each group. A significant deterioration compared to baseline was observed in both verum groups 4 hours after treatment (asterisked bars). As the pupil of the right eye was dilated acuity testing was only performed in the left eye.

Visual acuity deteriorated significantly in both verum groups post-treatment (p = 0.038 in group 1 and p = 0.004 in group 2). After 22 hours the baseline level was almost restored. The right eye was not tested as the pupil was dilated. See Figure 1 and Table 1.

**Table 1:** Visual acuity.

Group	Comparison	P-value
Low dose (Group 1)	+4 hours vs. baseline	0.038*
	+22 hours vs. baseline	0.363
High dose (Group 2)	+4 hours vs. baseline	0.004*
	+22 hours vs. baseline	0.518
Control group	+4 hours vs. baseline	0.363
	+22 hours vs. baseline	0.363

P-Values for the comparison between 4 and 22 hours after treatment and baseline. Significant values are highlighted and asterisked.

### Color Vision

**Figure 2:**
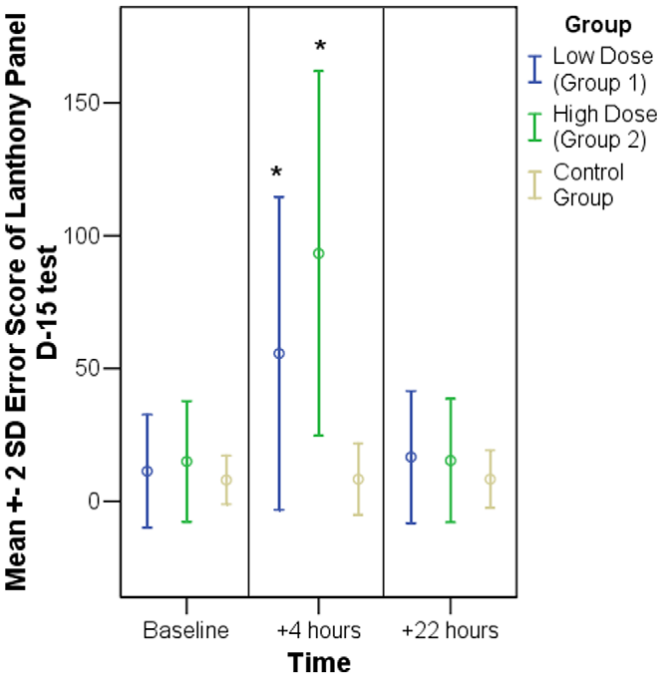
Lanthony Panel D-15 test of color vision. Time course of error score +/− 2 * standard deviation. Mean values for 6 subjects of each group. A significant deterioration compared to baseline was observed in both treated groups immediately after treatment (asterisked bars).

The most pronounced effect of ZK 200775 was seen on color vision as estimated with the desaturated Lanthony Panel D-15 test. The differences between the baseline and post-treatment error scores were significant for both treated groups being 22.16 and 39.15 in group 1 and 2, respectively, but only 0.17 in the control group. After 22 hours error scores of the treated groups returned to the baseline level (Figure 2 and Table 2).

**Table 2:** Lanthony Panel D-15 test of color vision.

Group	Comparison	P-value
Low dose (Group 1)	+4 hours vs. baseline	0.011*
	+22 hours vs. baseline	0.252
High dose (Group 2)	+4 hours vs. baseline	0.001*
	+22 hours vs. baseline	0.953
Control group	+4 hours vs. baseline	0.905
	+22 hours vs. baseline	0.908

P-Values for the comparison between 4 and 22 hours after treatment and baseline. Significant values are highlighted and asterisked.

### Dark vision

**Figure 3:**
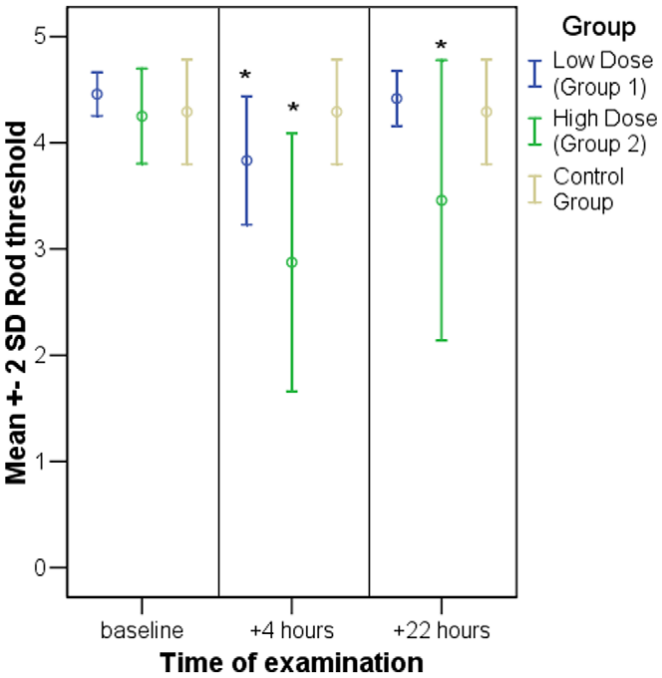
Rod threshold. Time course of mean rod threshold +/− 2 * standard deviation for both eyes of all probands. Significant changes (marked with an asterisk) compared to baseline occured in both treated groups after 4 hours and also after 22 hours in group 2.

Likewise, dark vision was impaired after drug administration. A significant elevation of the absolute rod threshold could be observed in both treated groups at 4 hours (p = 0.002 for both groups) and in group 2 even at 22 hours (p = 0.026). Figure 3 and Table 3 show that the deterioration of dark vision was dose-dependent and that no impairment was observed in the control group.

**Table 3:** Rod threshold.

	Group								
	Low Dose (Group 1)			High Dose (Group 2)			Control Group		
	Mean	STD	P-value	Mean	STD	P-value	Mean	STD	P-value
Rod threshold at baseline [log]	4.46	0.10		4.25	0.22		4.29	0.25	
Rod threshold at 4 hours [log]	3.83	0.30	0.002*	2.88	0.61	0.002*	4.29	0.25	X
Rod threshold at 22 hours [log]	4.42	0.13	0.363	3.46	0.66	0.026*	4.29	0.25	X

Mean values and standard deviation of rod threshold and t-test results for comparison to baseline. Significant values are highlighted and asterisked. T-test of control group was not possible due to equal mean values at all times.

**Figure 4:**
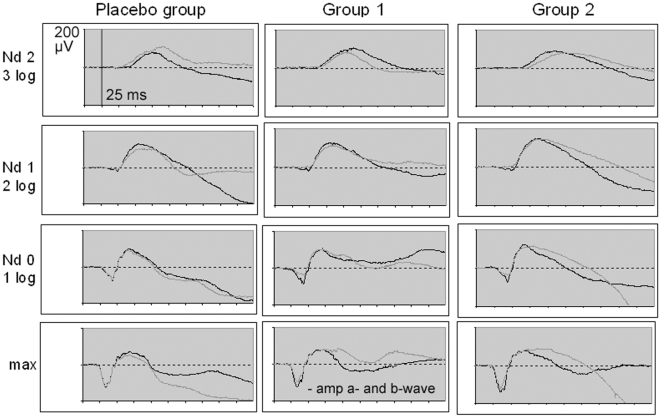
Mean dark adapted full-field ERG. Recordings for each group and flash strength at baseline (black line) and 4 hours after treatment (gray line). The effect of ZK200775 was subtle but recognizable. Statistically significant amplitude reduction of the a- and b-wave occured in the low dose group after 4 hours.

**Figure 5:**
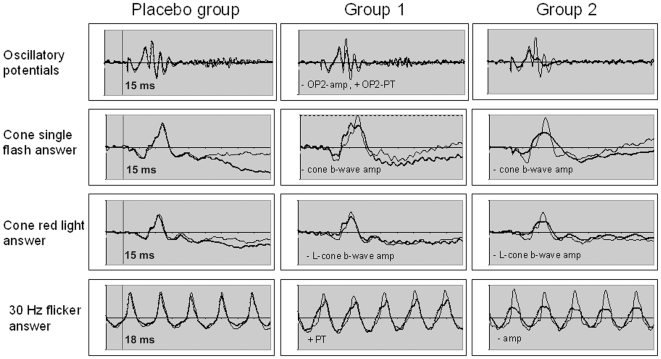
Mean cone-ERG. Recordings of each group for baseline (thin line) and 4 hours after treatment (thick line). An almost dose-dependent amplitude decrease can be seen in all cone-ERG modalities. Significant increments (+) and decrements (−) of the deflections as compared to baseline are marked in the according graphs (PT = peak time, amp = amplitude).

**Figure 6:**
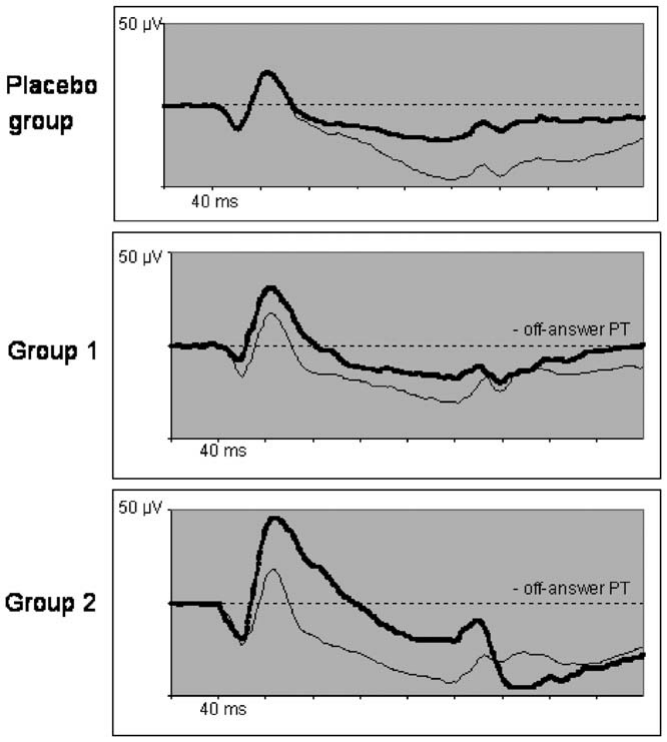
Mean ON-OFF-ERG. Recordings of each group for baseline (thin line) and 4 hours after treatment (thick line). In group 1 the recordings of only 4 of 6 probands were considered due to unrecognizable OFF-answers in 2 probands. An increase of on-answer amplitude in both treated groups seems obvious but was statistically not significant. However, there was a significant reduction of OFF-answer peak time (PT) in both treated groups 4 hours after treatment.

**Figure 7:**
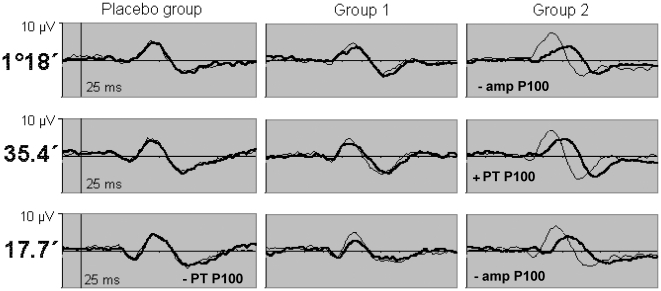
Mean pattern visual evoked potential. Recordings for each group and check size. Stimulation of the left eye with natural pupil. Thin lines: baseline recordings, thick lines: recordings 4 hours after treatment. This figure shows a virtually dose-dependent amplitude decrease and peak time prolongation. Significant increments (+) and decrements (−) of the deflections as compared to baseline are marked in the according graphs (PT = peak time, amp = amplitude).

ZK 200775 administration exerted various effects on the electrophysiologic recordings. In the statistic calculation we included amplitudes and peak times as measured in the single recordings. The mean values that can be read out of the diagrams (Figure 4, 5, 6, and 7) may differ from the mean values calculated from the single recordings. We hold that in case of ambiguous results the summed up responses as seen in the mentioned diagrams most clearly demonstrate the effects of the drug on electrophysiology.[Fig pone-0012111-g008]

**Figure 8 pone-0012111-g008:**
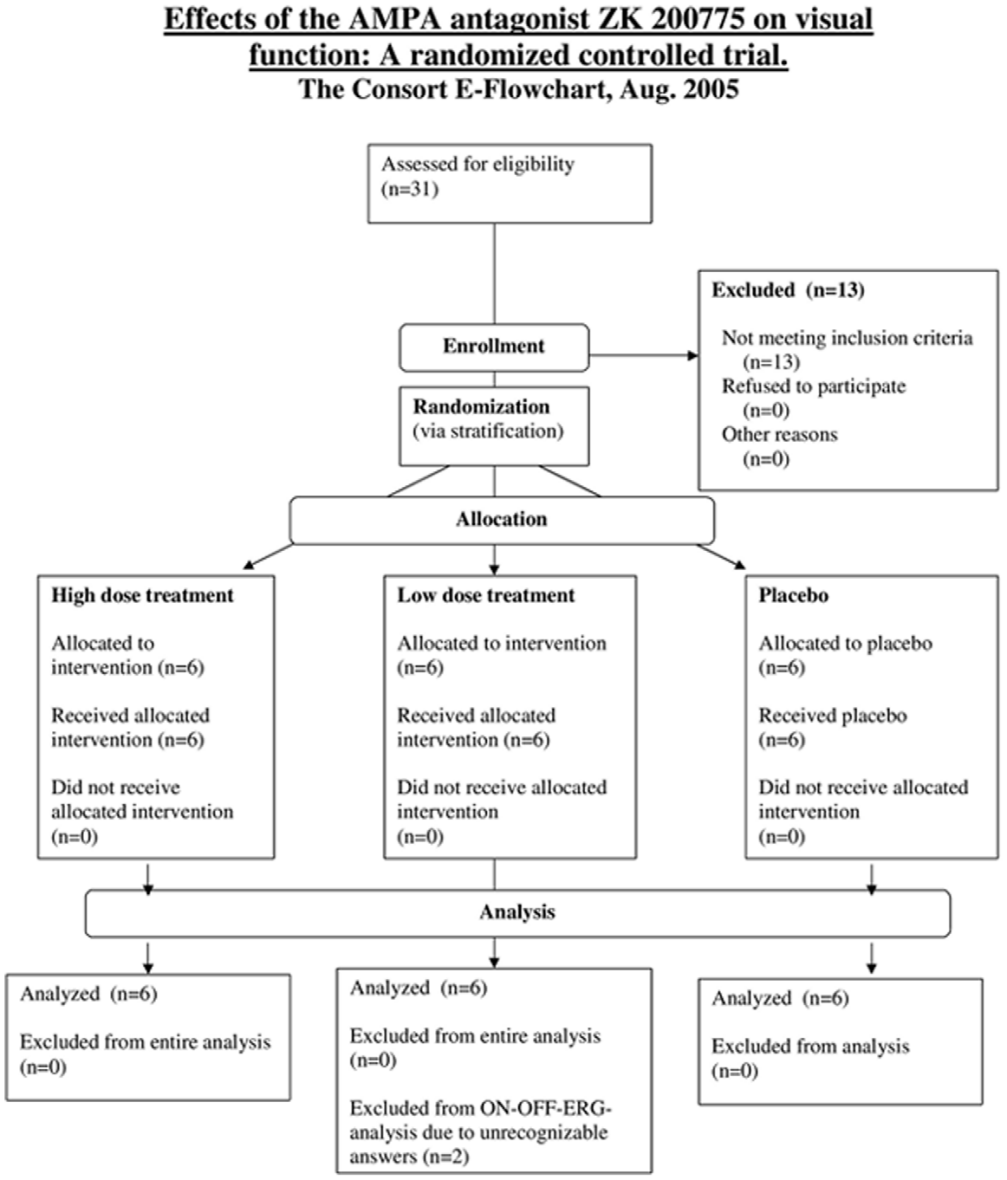
Consort flow chart.

### Scotopic Electroretinogram (ERG)

There was a small but obvious difference of the b-wave morphology before and after drug administration (Figure 4, Table 4). In the dark-adapted ERG elicited by higher stimulus intensities the b- and a-waves were reduced in amplitude, reaching a significant only in group 1. The b/a-ratio was slightly and unsignificantly elevated in both treated groups after four hours. Compared to this mild effect on the ERG a- and b-wave the dark adapted oscillatory potentials (OP) were significantly reduced in amplitude and showed significantly altered peak times in group 1 after 4 hours and in group 2 after 22 hours (Figure 5, Table 5).

**Table 4:** Scotopic ERG.

		Group								
		Low Dose			High Dose			Control Group		
		(Group 1)			(Group 2)					
		Mean	STD	P-value	Mean	STD	P-value	Mean	STD	P-value
**BASELINE**	Amplitude of the maximum a-wave [µV]	253.58	61.52		284.83	37.55		248.34	70.46	
	Peak time of the maximum a-wave [ms]	14.00	0.63		14.83	0.75		14.17	0.75	
	Amplitude of the maximum b-wave [µV]	414.96	119.75		451.61	58.97		392.52	72.56	
	Peak time of the maximum b-wave [ms]	42.33	2.50		42.67	3.01		43.5	4.32	
	b/a-ratio	1.63	0.21		1.59	0.14		1.63	0.33	
**4 HOURS**	Amplitude of the maximum a-wave [µV]	196.48	73.40	0.03*	247.99	70.60	0.293	253.64	66.20	0.77
	Peak time of the maximum a-wave [ms]	14.17	0.41	0.611	15.33	0.52	0.076	14.17	0.75	1.00
	Amplitude of the maximum b-wave [µV]	339.90	113.63	0.008*	408.12	111.78	0.407	369.17	63.45	0.298
	Peak time of the maximum b-wave [ms]	44.17	3.19	0.33	45.83	6.40	0.195	43.67	3.01	0.842
	b/a-ratio	1.77	0.29	0.076	1.67	0.26	0.305	1.50	0.22	0.225
**22 HOURS**	Amplitude of the maximum a-wave [µV]	257.57	67.54	0.855	246.74	55.92	0.212	249.19	48.77	0.959
	Peak time of the maximum a-wave [ms]	14.17	0.75	0.695	14.67	0.52	0.611	14.00	0.89	0.695
	Amplitude of the maximum b-wave [µV]	396.97	106.19	0.365	404.89	66.81	0.223	375.95	40.77	0.539
	Peak time of the maximum b-wave [ms]	42.00	2.68	0.809	41.67	3.56	0.536	43.50	3.56	1.00
	b/a-ratio	1.56	0.24	0.543	1.67	0.25	0.338	1.54	0.24	0.342

Mean data of the scotopic combined rod and cone a- and b-wave amplitude and peak time and of b/a-ratio. Significant changes compared to baseline (t-test) occured in the low-dose group and are highlighted and asterisked. Note that mean values were calculated from the individual recordings taken from each proband and may thus differ from the values depicted in Figure 4.

**Table 5:** Oscillatory potentials.

	Group								
	Low Dose (Group 1)			High Dose (Group 2)			Control Group		
	Mean	STD	P-value	Mean	STD	P-value	Mean	STD	P-value
OP2 amplitude at **baseline** [mikrovolt]	53.8	14.2		46.7	13.7		44.6	15.4	
OP2 peak time at **baseline** [ms]	22.8	0.31		22.9	0.33		22.5	0.45	
OP2 amplitude at **4 hours** [mikrovolt]	30.4	9.34	0.01*	19.4	8.17	0.06	41.8	16.3	0.425
OP2 peak time at **4 hours** [ms]	23.6	0.62	0.025*	19.8	3.39	0.061	22.4	0.49	0.363
OP2 amplitude at **22 hours** [mikrovolt]	48	15	0.243	26.3	13.6	0.04*	41.4	12.9	0.343
OP2 peak time at **22 hours** [ms]	23	0.82	0.465	24.3	0.96	0.013*	22.6	0.54	0.611

Mean values and standard deviation of amplitude and peak time and p-values for the comparison to baseline. Significant changes compared to baseline (t-test) are highlighted and asterisked. Note that mean values were calculated from the individual recordings taken from each proband and may thus differ from the values depicted in Figure 5.

### Photopic ERG

All photopic ERG responses showed a dose-dependent amplitude reduction in the treated groups (Figure 5, Table 6). Significant reductions occured in both treated groups after 4 hours and in group 2 even after 22 hours. Peak times were also reduced with the difference reaching a significant level only as to the cone flicker answer in group 1 after 4 hours.

**Table 6:** Cone-ERG.

		Group								
		Low Dose (Group 1)			High Dose (Group 2)			Control Group		
		Mean	STD	P-value	Mean	STD	P-value	Mean	STD	P-value
**BASELINE**	Cone single flash answer amplitude [µV]	155.3	44.2		149.2	29.8		120.3	24.3	
	Cone single flash answer peak time [ms]	32.2	1.2		31.2	1.3		31	1	
	Cone red light answer amplitude [µV]	95.39	17.4		91.88	31.7		77.41	17.2	
	Cone red light answer peak time [ms]	28.14	0.98		28.3	0.63		27.9	0.88	
	Cone flicker answer amplitude [µV]	135.3	35.8		137.6	29.1		122.1	31	
	Cone flicker answer peak time [ms]	27.24	1.4		26.52	1.24		26.64	1.02	
**+4 HOURS**	Cone single flash answer amplitude [µV]	106.3	40.1	0.003*	76.94	25.4	0.001*	114.2	20.4	0.456
	Cone single flash answer peak time [ms]	31.3	3.32	0.605	30	2.98	0.328	31.2	1.05	0.363
	Cone red light answer amplitude [µV]	67.5	24.5	0.038*	40.78	10.8	0.005*	74.73	13.9	0.744
	Cone red light answer peak time [ms]	28.2	1.24	0.945	26.6	3.23	0.233	27.9	0.59	1
	Cone flicker answer amplitude [µV]	108.1	39.3	0.108	78.49	15.9	0.000*	111.2	15.1	0.311
	Cone flicker answer peak time [ms]	30.12	1.73	0.002*	26.16	1.18	0.624	26.52	0.96	0.363
**+22 HOURS**	Cone single flash answer amplitude [µV]	136.1	35.6	0.233	108.1	22.6	0.002*	113.9	14.4	0.562
	Cone single flash answer peak time [ms]	31.6	1.26	0.497	30.7	1.85	0.383	31.4	0.82	0.235
	Cone red light answer amplitude [µV]	80.51	15.1	0.123	54.81	13.3	0.012*	80.91	19.8	0.638
	Cone red light answer peak time [ms]	27.8	1.55	0.419	27.9	0.7	0.444	28	0.54	0.809
	Cone flicker answer amplitude [µV]	122.2	32.8	0.456	102	25.4	0.021*	114.2	23	0.503
	Cone flicker answer peak time [ms]	27.24	1.06	1	27.36	1.2	0.058	26.52	0.96	0.363

Mean values and standard deviation of amplitude and peak time and p-values for the comparison to baseline. Significant changes (t-test) are highlighted and asterisked. Note that mean values were calculated from the individual recordings taken from each proband and may thus differ from the values depicted in Figure 5.

### On-OFF ERG

In group 1 the recordings of only 4 of 6 probands were considered due to unrecognizable OFF-answers in 2 probands.

The amplitude of the ON-response virtually showed a dose-dependent increment in both treated groups without reaching a significant level (Figure 6, Table 7). Peak times of the OFF-response were significantly reduced in both treated groups after 4 hours (p = 0.026 in group 1 and p = 0.021 in group 2), the ON-response was delayed after 22 hours in group 2 (p = 0.021). The OFF-amplitudes were only slighty affected.

**Table 7:** ON-OFF-ERG.

		Group								
		Low Dose (Group 1)			High Dose (Group 2)			Control Group		
		Mean	STD	P-value	Mean	STD	P-value	Mean	STD	P-value
**BASELINE**	Amplitude of the on answer [mikrovolt]	49.5	22.9		59.71	25.6		39.66	13.9	
	Peak time of the on answer [ms]	46.8	3.6		46.8	2.85		47.07	3.93	
	Amplitude of the off answer [mikrovolt]	14.4	7.08		14.6	7.5		9.86	3.34	
	Peak time of the off answer [ms]	226	1.46		225.3	1.8		224.8	1.75	
**+4 HOURS**	Amplitude of the on answer [mikrovolt]	59.1	38.2	0.322	70.77	20.2	0.211	42.59	12	0.552
	Peak time of the on answer [ms]	46.5	4.06	0.75	49.29	6.05	0.215	46.27	3.66	0.229
	Amplitude of the off answer [mikrovolt]	6.55	4.18	0.223	14.66	9.89	0.986	10.42	3.04	0.771
	Peak time of the off answer [ms]	219	2.56	0.026*	221.3	1.87	0.021*	225.6	1.96	0.348
**+22 HOURS**	Amplitude of the on answer [mikrovolt]	46	16.1	0.531	56.34	33	0.579	47.36	21.7	0.453
	Peak time of the on answer [ms]	44.7	3.41	0.199	45.07	2.36	0.021*	45.73	4.19	0.129
	Amplitude of the off answer [mikrovolt]	8.24	3.05	0.11	10.99	3.59	0.324	12.96	4.96	0.151
	Peak time of the off answer [ms]	225	3.22	0.432	224.1	1.15	0.235	225.3	1.57	0.484

Mean values, standard deviation of amplitude and peak time and p-values for comparison to baseline. Significant changes are highlighted and asterisked. Note that mean values were calculated from the individual recordings taken from each proband and may thus differ from the values depicted in Figure 6.

### Pattern visual evoked potential (pVEP)

Also the pVEP was affected by the administration of ZK 200775 (Figure 7, Table 8). The post-treatment pVEP could be identified easily due to characteristic morphologic changes. We saw a dose-dependent peak-time prolongation and amplitude decrease of the P100 complex. Significant changes were seen in group 2 after four hours concerning the amplitude for the 1°18′ and 17.7′ pattern (p = 0.028 and 0.08) and the peak time of the 35.4′ pattern (p = 0.038). Surprisingly, there was a peak time reduction for the check size 17.7′ after 4 and 22 hours (p = 0.032 and 0.044) in the control group.

**Table 8:** PVEP.

		Group								
		Low Dose			High Dose			Control Group		
		(Group 1)			(Group 2)					
		Mean	STD	P-value	Mean	STD	P-value	Mean	STD	P-value
**BASELINE**	1°18′ amplitude [µV]	7,11	2,01		9,38	4,47		7,44	2,68	
	1°18′ peak time [ms]	107,83	6,31		109,67	6,35		115,00	6,20	
	35.4′ amplitude [µV]	7,74	1,16		9,15	5,17		7,43	3,51	
	35.4′ peak time [ms]	109,83	5,34		112,00	5,44		115,83	6,15	
	17.7′ amplitude [µV]	8,35	3,07		10,04	5,16		7,99	2,79	
	17.7′ peak time [ms]	114,50	5,05		115,83	5,31		121,33	6,53	
**+4 HOURS**	1°18′ amplitude [µV]	6,24	1,11	0,354	7,12	4,09	0,028*	6,46	2,94	0,361
	1°18′ peak time [ms]	111,67	9,61	0,112	122,33	17,99	0,064	115,00	7,40	1
	35.4′ amplitude [µV]	6,53	1,42	0,11	7,30	3,17	0,194	7,43	3,85	0,997
	35.4′ peak time [ms]	112,33	9,33	0,273	123,67	13,29	0,038*	115,33	9,56	0,843
	17.7′ amplitude [µV]	7,17	3,80	0,58	6,66	3,87	0,08*	8,33	3,10	0,753
	17.7′ peak time [ms]	118,17	8,04	0,212	126,00	13,43	0,061	118,83	5,95	0,032*
**+22 HOURS**	1°18′ amplitude [µV]	6,42	1,51	0,495	9,25	4,54	0,465	6,95	2,86	0,529
	1°18′ peak time [ms]	112,50	6,60	0,158	110,20	4,32	0,17	117,83	7,44	0,186
	35.4′ amplitude [µV]]	7,61	2,56	0,915	10,43	5,35	0,657	7,79	4,31	0,745
	35.4′ peak time [ms]	110,17	8,98	0,868	109,60	5,59	0,871	116,67	5,32	0,601
	17.7′ amplitude [µV]	9,71	3,98	0,313	10,99	6,85	0,292	8,06	4,05	0,961
	17.7′ peak time [ms]	113,67	5,01	0,652	122,00	4,82	0,059	119,43	5,53	0,044*

Mean values, standard deviation and p-values for amplitude and peak time of P100. Significant changes compared to baseline (t-test) are highlighted and asterisked. Note that mean values were calculated from the individual recordings taken from each proband and may thus differ from the values depicted in Figure 7.

## Discussion

ZK 200775 is a glutamate antagonist at the AMPA receptor and was tested in clinical trials for its neuroprotective effects in cerebral ischemia. Probands receiving ZK 200775 constantly reported an alteration of their visual perception. This clinical trial offered the opportunity to objectify the impact of an AMPA receptor blockade on the visual system by psychophysical and electrophysiological means in vivo.

The antagonization of AMPA receptors by ZK200775 led to alterations of color and dark vision, visual acuity, cone ERG modalities and the pVEP and to a lesser extent on the scotopic ERG. ZK 200775 did not alter eye morphology, functioning of the extraocular muscles, binocular vision, visual fields or the pupil.

All subjective and objective symptoms found in this study which could be correlated to the administration of the AMPA antagonist ZK 200775 are most likely reversible. All parameters that were altered immediately after administration returned to the normal range or showed a strong tendency to this after 22 hours.

Most of our psychophysical and electrophysiologic findings are consistent with the localization of AMPA receptors in the retinal layers as it was reported by previous experimental research. There are many indications that AMPA-antagonists compromise the glutamate action of the middle and inner retina to a larger extent than the function of outer retinal layers. The alterations of the flash ERG were subtle and the components representing photoreceptor function (flash ERG a-wave) were only mildly reduced. On the other hand, ERG OFF-response and the OPs, all representing more interior retinal function, were reduced more markedly in the treated groups.

Our results especially hint at the affection of horizontal and amacrine cell function. It is known that both cell types play a role in color vision as well as in the integration of luminance detection. A functional defect of these cells may therefore lead to a deterioration of color vision as well as to a reduction of dark vision. The altered OPs can be regarded as the electrophysiologic correlate of the impaired amacrine cell function.

Separating the ON- and OFF-pathway, our results are largely in agreement with earlier findings. Rods connect primarily to ON bipolar cells, whereas cones connect to ON or OFF bipolar cells. AMPA receptors mainly participate in the OFF-pathway but are also said to be expressed by some ON bipolar cells [19], [20], [21], [22]. While rod bipolar cell dysfunction and synaptic failure between rods and rod bipolar cells and between cones and cone ON bipolar cells are well known from a couple of retinal disorders (e.g. congenital stationary night blindness), failure of the cone OFF-pathway is rare. ZK200775 mildly altered the ON-pathway (scotopic ERG and dark vision), the main effect could be observed in the OFF-pathway and postreceptorally (color vision, visual acuity, cone ERG modalities).

The most conspicuous finding of this study is the deterioration of color vision as measured by the Panel D-15 test. After infusion of ZK200775 the error-score increased significantly in both treated groups being an almost predictable result of the OFF-pathway blockade.

The preponderance of non-NMDA receptors in the OFF-pathway also makes an impairment of central visual acuity after a partial pharmacological blockade very likely. In this study the treated probands showed an impairment of visual acuity between one and two lines.

On the other hand we observed an impairment of dark vision of approximately 1 log unit that would not be primarily expected from a pure OFF-pathway blockade. Beside the potential effect of horizontal cell impairment on scotopic vision additional effect can be hypothesized. However, recent findings from animal experiments questioning the concept of two strictly independent rod and cone pathways make it well conceivable that an AMPA blockade influences scotopic vision as seen in our trial:

1. Several authors reported the expression of AMPA receptors even in ON bipolar cells [19],[20],[21],[22].
2. Concerning the rodent retina, Hack et al. and Tsukamoto et al. proved the existence of small populations of OFF bipolar cells that connect to both cones *and* rods [23], [24], [25].
3. The rod pathway is directly connected to the cone pathway via gap-junctions between cone and rod photoreceptors. Therefore, the cone OFF bipolar cells also recieve input from rods [23], [24], [26], [27], [25], [28].

Concerning the dark adapted full-field ERG, ZK 200775 decreased both the a- and b-wave. At high stimulus intensities amplitudes were reduced to a greater extent than at small intensities. The b/a-ratio was slightly elevated in both treated groups four hours after administration without reaching a significant level.

The b-wave of the dark-adapted ERG is generated mainly by the rod bipolar cells, the a-wave appears at higher stimulus intensities and reflects the activity of the rods. Again, as to dark vision, the small influence on the dark-adapted ERG can be explained with the minor part AMPA receptors are assumed to play in the ON-pathway and therefore in scotopic vision. The dependance between stimulus intensity and ERG alteration can be explained by the increasing influence of the cones on the ERG morphology at brighter flashes [29].

In the ON-OFF-ERG flashes of longer duration are used to separate the activities of both pathways. The ON-answer consists of the a- and b-wave. The difference to the scotopic ERG is that the photopic ERG b-wave results from opposing field potentials not only generated by depolarizing (ON) but also by hyperpolarizing (OFF) bipolar and horizontal cells as well as third-order neurons [30], [31]. Sieving et al. established a “push-pull model' of these cell types [32].

We observed a dose-dependent ON-response amplitude increment which corresponds to the results of Sieving et al. and probably reflects the fading influence of opposing third order OFF-neurons being blocked by ZK 200775.

The OFF-answer (or d-wave) appears after stimulus cessation and can be separated from the b-wave only if the stimulus is long enough. It is said to be generated in OFF-bipolar cells. The OFF-amplitudes we measured were almost unaffected by the drug but surprisingly OFF-peak times were shortened significantly. These are paradoxical findings that defy explanation with the hitherto existing data. Earlier research using DNQX to selectively block AMPA and KA GluRs even showed a reversed polarity of the OFF-answer [31].

The rod-response to flashing stimuli is too slow to allow a stimulation of rods by flickering light at 30 Hz. Therefore the flicker-ERG is a parameter of cone function. In the treated groups, the cone-mediated answers showed reduced amplitudes (flicker-response and red light and single-flash answer) and delayed peak times (flicker-response). Yet, all cone answers were still recognizable. This again proves the influence of ZK200775 on the OFF-pathway but argues against its complete blockade.

The VEP is a parameter for the integrity of the entire visual pathway [29]. The reduction of the pVEP amplitudes may be explained by the influence of the drug on the ganglion cell function or even on the middle and outer retinal layers. Moreover, we recorded significantly prolonged implicit times of the P100 complex which is commonly regarded as a marker of impaired nerval conduction like in optic nerve inflammation. We assume that upstream retinal alterations have led to a secondary peak time prolongation. The peak time reduction in the control group after treatment supposedly arises from the principal variability of this electrophysiologic method.

Our results maintain that AMPA receptors predominate in the OFF-pathway and play a minor role in the ON-pathway. Nevertheless, the interdependence of both pathways becomes apparent. Surprising findings were the partial preservation of visual acuity, deteriorated dark vision, the persistent OFF-answer with it's shortened peak time and the peak time delay of the pVEP.

Although the results of this study do not allow a precise definition of the role of AMPA receptors in the human retina they render some information concerning the relevance of glutamate for vision and for the components of the electroretinogram. Whereas the latter could also be studied in animals, this study offered a unique chance to elucidate the impact of AMPA on the psychophysically examined vision in humans.

## Materials and Methods

The protocol for this trial and supporting CONSORT checklist are available as supporting information; see Checklist S1 and Protocol S1.

This clinical trial was approved by the “Ethikkommission der Berliner Aerztekammer” (Ethics Committee of the Berlin Medical Association) and was conducted according to the Declaration of Helsinki. All probands gave written informed consent to participate in the study.

18 healthy probands were recruited for this study. Recruitment was done vía random selection of probands from the central Schering database on the basis of inclusion and exclusion criteria (Table S6). Recruitment period lasted from Februar to July 1997. All probands were of male sex, the mean age was 61 years (57 to 69 years). None of the probands participated in other phase I trials concerning ZK200775.

For recruitment of the probands, a physical and ophthalmological examination screened out any relevant medical or vision disorders. If the proband was included in the study this checkup also served as baseline examination and encompassed:

- Medical and opthalmological history
- Visual acuity
- Stereopsis (Titmus-test)
- Eye position, motility, pupillary reaction
- Color vision (Lanthony Panel D-15 desaturated)
  - To calculate the error score of the Panel test the differences of the values of every two connected Panels were calculated and added (sums up to 15 for an entirely correct test). Then, 15 was subtracted from the sum (as the error score for a correct test must be 0).
- Central visual field (Amsler grid)
- Applanation tonometry (Goldmann)
- Slit lamp examination of the anterior segment
- Funduscopy.

Randomization was executed via stratification. A sequential (‘dose escalation’) strategy beginning with the low-dose stratum and continuing to the high-dose stratum was planned. There were two strata, one for each dosage group (0.03 an 0.75 mg/kg/h). In every stratum there were two possible treatments: ZK200775 or placebo. The verum-group included 6 participants, the placebo group 3 participants per dosage group. In total, 18 participants were allocated to the treatments with a ratio of 2∶1 (12 ZK200775 [6 with a dosage of 0.03 mg/kg/h and 6 with a dosage of 0.75 mg/kg/h], 6 Placebo [0.9% sodium chloride solution], see Figure 8).

All participants as well as all persons involved in drug application and examination were blinded from drug or placebo assignment inside the group. There was no blinding between strata. An independent team with access to a randomization list prepared the investigational product and the infusion system on the morning of treatment. Another randomization list was deposited in the trial master file. Emergency envelopes were stored in the Schering Institute for Clinical Pharmacology and were also available for the investigator.

There were no explicit outcome measures. The aim of the study was to quantifiy the ophthalmologic effects of ZK200775 by adequate examinations.

On the day of drug administration an additional ophthalmological examination was performed before, immediately after (4 hours after treatment onset) and 22 hours after onset of drug administration. These examinations comprised the points mentioned above except history and tonometry. Additionally, the rod absolute threshold was tested in the right eye with dilated pupil with a Hartinger adaptometer (Carl Zeiss, Jena) after 30 minutes of dark adaptation.

Electrophysiologic examinations were also performed before, immediately after and 22 hours after drug administration. For the full-field ERG the pupil of the right eye was dilated with tropicamide. For the remaining examinations the pupil of the left eye remained neutral. For electrophysiological recordings a Nicolet Bravo® unit was employed (Nicolet Germany, Hoechberg). Thread conjunctival electrodes were used for electroretinography. All recordings were made according to the Standard of the International Society of Clinical Electrophysiology of Vision (ISCEV).

### Full-field electroretinogram (ERG)

Four scotopic responses with increasing stimulus intensity following dark adaptation.

### Oscillatory potentials

Initial dark adaptation. High pass filter: 100 Hz.

### Cone-ERG

Initially ten minutes of light adaptation (30 cdm^−2^). Recording of single-flash cone response, cone red light answer and response to 30 Hz flicker.

### ON-OFF ERG

Stimulation with red flashes of a duration of 200 ms delivered through light emitting diodes following light adaptation.

### Pattern-reversal visual evoked potential (pVEP)

Check size of 17.7′, 35.4′ and 1,18° at 3 reversals per second. Stimulation of the left eye with the natural pupil.

Mean amplitude and peak time was calculated for all tested individuals at each measurement (before administration, immediately after treatment [4:00 hours] and 22:00 hours after the onset of treatment).

Post-treatment values at 4 and 22 hours (visual acuity, error score of the Panel test, rod threshold, peak time and amplitude of the electrophysiologic recordings) were compared to baseline measurements using the t-test. P-values <0.05 were regarded as statistically significant.

## Supporting Information

Checklist S1 Consort Checklist
(0.19 MB DOC)

Protocol S1 Trial Protocol
(108.83 MB PDF)

Figure S1 Glutamate receptor subtypes.
(0.02 MB TIF)

Table S1 Ionotropic glutamate receptor expression in retinal neurons and retinal layers. Immunocytochemistry, in situ hybridization and polymerase chain reaction. Based on Webvision, completed by the authors (http://webvision.med.utah.edu, author: Vikki P. Connaughton). Some papers could not be considered in this survey.
(0.15 MB DOC)

Table S2 Eye position. Data for both eyes for each group before infusion and 4 and 22 hours after infusion of ZK 200775. No significant changes occurred.
(0.05 MB DOC)

Table S3 Eye Motility. Data for both eyes and each group before infusion and 4 and 22 hours after infusion of ZK 200775. No significant changes occurred.
(0.05 MB DOC)

Table S4 Pupil light response. Data of the left eye for each group before infusion and 4 and 22 hours after infusion of ZK 200775. No significant changes occurred.
(0.03 MB DOC)

Table S5 Amsler Card examination. Data of the left eye for each group before infusion and 4 and 22 hours after infusion of ZK 200775. No significant changes occurred.
(0.04 MB DOC)

Table S6 Inclusion and exclusion criteria.
(0.04 MB DOC)
