# Supplementary material for: Effects of the AMPA Antagonist ZK 200775 on Visual Function: A Randomized Controlled Trial
Source: PLoS One. 2010 Aug 12;5(8):e12111. doi: 10.1371/journal.pone.0012111 (PMC2920815; doi:10.1371/journal.pone.0012111)
Supplement: Protocol S1 — Trial Protocol (108.83 MB PDF) [file pone.0012111.s002.pdf]

SCHERING	Prüfplan **Nr. 96048**	**ENDFASSUNG**
**Klinische Pharmakologie**		Seite: 1
		Umfang: 44 Seiten<br>Datum: 10 Feb. 97

Fly Sheet 1

Ophthalmologische Untersuchungen nach 4-stündiger Infusion mit ZK 200775 bei älteren gesunden männlichen Freiwilligen

Datum: 05. Feb. 97 **Autor: Y. Tauchmann / Dr. Th. Staks**

Der Prüfplan wird mit einem Textverarbeitungssystem erstellt und trägt keine Unterschriften. Die Unterschriften befinden sich auf einem separaten Unterschriftendokument.

Prüfziel

Der AMPA-Antagonist ZK 200775 soll auf seine retinalen bzw. ophthalmologischen Effekte nach einer 4-stündigen Infusion untersucht werden.

V e r t r a u l i c h

Anlagen

- Spezielle Probandeninformation incl. Einverständniserklärung

Verteiler

	Version vor Ethik-Kommission	Endfassung
Leiter der klinischen Prüfung	1x	1x
Betreuende(r) Arzt/Ärztin	1x	1x
Technische(r) Mitarbeiter(in) IKP	1x	1x
Prüfungseinrichtung/Standards IKP	2x	2x
ZÄD IKP	1x	1x
Trial Master File IKP	Original	Original
Leitung Inst. Klinische Pharmakologie	1x	1x
Supervisor IKP	1x	1x
Medical Writing SGE		1x
GCP-Auditing	1x	1x
Biometrie SGE	1x	1x
Leiter klin. Entw. der zuständigen SGE	1x	1x
Dossier Management		1x
Ethik-Kommission	8x	1x
Übersetzer für englische Endfassung		1x
	verteilt am: ____________	verteilt am: 13/02/97

Fly Sheet 2: Synopsis

Prüfungstitel:	Ophthalmologische Untersuchungen nach 4-stündiger Infusion mit ZK 200775 bei älteren gesunden männlichen Freiwilligen.	
Leiter der klinischen Prüfung:	Dr. Thomas Staks	
Prüfungsort(e):	Institut für Klinische Pharmakologie, Sellerstraße 31, 13342 Berlin<br>Klinik und Poliklinik für Augenheilkunde der Charite´und des Virchow-Klinikums	
Publikationen (Referenzen):	nicht zutreffend	
Prüfungszeitraum:	(Plandatum erster Teilnehmereinschluß) Februar 1997<br>(Plandatum letzter Teilnehmerabschluß) Juli 1997	Klinische Phase I
Prüfungsziele:	Erfassung und Quantifizierung von ZK 200775 induzierten ophthalmologischen Nebenwirkungen.	
Methodologie:	Doppelblind, placebo-kontrolliert über zwei Dosisstufen 0,3 und 0,75 mg/kg/h<br>Ophthalmologische Untersuchung: Anamnese, Spaltlampenmikroskopie und Funduskopie.<br>Psychophysische Sehtestung: Visus und Refraktionsbestimmung, Stereopsisprüfung, Farbwahrnehmungstest (Farnworth Panel D 15 desat.), sowie Messung der Stäbchen- Endschwelle nach Dunkeladaptation.<br>Elektrophysiologische Untersuchung: Helligkeits- ERG, Muster- ERG und VEP.	
Gesamtzahl der Probanden (geplant):	18	
Probandenzahl je Behandlung (geplant):	9 je Dosisstufe, davon 6 Verum und 3 Placebo	
Diagnose und Hauptkriterium für den Einschluß:	Gesunde Männer im Alter von 55- 65 Jahren.	
Prüfpräparat:	Infusionslösung ZK 200775; übergeordnete SH-Nr.: SH Y 608 ZY	
Dosis:	2 Dosisstufen: 0,3 und 0,75 mg/kg/h; Einmalgabe	
Art der Verabreichung:	Intravenöse Infusion über 4 Stunden	
Chargen Nr.:		
Dauer der Behandlung:	Einmalige Gabe über 4 Stunden	
Vergleichsbehandlung:	Placebo: 0,9% Natriumchloridlösung; 3 Teilnehmer pro Dosisstufe	
Dosis:	Volumengleich zur Verumdosis	
Art der Verabreichung:	Intravenöse Infusion über 4 Stunden	
Chargen Nr.:	C 2171	
Kriterien für die Auswertung:		
Wirkungen:	Ophthalmologische Untersuchung: Psychophysische Sehtestung, Visusbestimmung, Stereopsisprüfung, Farbwahrnehmungstest (Farnworth Panel D 15 desat.).<br>Elektrophysiologische Untersuchung: Helligkeits- ERG, Muster-ERG, VEP	
Sicherheit:	Vor- und Nachuntersuchung, adverse events.	
Statistische Methoden:	Deskriptive Statistik, Grafiken	

Fly Sheet 3: Verantwortlichkeiten

Name (intern)	Adresse	Verantwortlichkeit
Dr. Th. Staks	Schering AG, Institut für Klinische Pharmakologie	Leiter der klinischen Prüfung und Vertriebsleiter Klinische Prüfung lt. AMG
Fr. Y. Tauchmann	Schering AG, Institut für Klinische Pharmakologie	Prüfärztin
Fr. E. Schönfelder-Mahdjour	Schering AG, Institut für Klinische Pharmakologie	Prüfärztin
Fr. I. Zimmer	Schering AG, Institut für Klinische Pharmakologie	prüfungs-verantwortliche TA, Qualitätskontrolle
Dr. K. Vick	Schering AG, Institut für Klinische Pharmakologie, 13342 Berlin	Prüfungseinrichtung/ Standards
Dr. K. Fichte	Biometrie SAG	Biometrie
H. J. Hartmann	Schering AG, Institut für Klinische Pharmakologie, 13342 Berlin	Teilnehmer-Einschluß- und Abschlußunter-suchungen
Dr. C. Stürzebecher	Schering AG, SGE Therapeutika	Klinischer Entwicklungsleiter/ Sponsor
Dr. W. Seifert	Schering AG, Institut für Klinische Pharmakologie	Sponsor Bereitstellung der Ressourcen

Name (extern)	Adresse	Verantwortlichkeit
Dr. Rüther	Klinik und Poliklinik für Augenheilkunde Charité und Virchow-Klinikum, Augustenburgerplatz 1, 13353 Berlin	Prüfarzt, Durchführung der ophthalmo-logischen Tests
Fr. Bohne	Kinik und Poliklinik für Augenheilkunde Charité und Virchow-Klinikum Augustenburgerplatz 1, 13353 Berlin	Technische Assistentin
Dr. H. Tabel	W & T GmbH, Waldstraße 14, 10551 Berlin	Laboranalytik

Fly Sheet 4: Pharmazeutische Daten

Daten zu Prüfpräparat(en) und Formulierung(en)		
**Arzneistoff(e)**		
ZK Nummer	ZK 200775	
Freiname	Quinophon	
Chargennummer	15052243	
Analysennummer	00122/95	
Stabilität	06/1997	
Hersteller	Schering AG	
**Formulierung(en)**		
Art der Formulierung	Lyophylisat für Infusionslösung in folgener Zusammensetzung: 270,00 mg ZK 200775 + 125,00 mg Mannit + 250,00 mg Natriumhydroxid; SH L 608 R als Rekonstitutionslösung	
SH Nummer	SH Y 608 A, Lyophilisat (übergeordnete SH Y 608 ZY)	
Handelsname	--	
Chargennummer	D 2891	
Analysennummer	12284/95 (SH Y 608 A); 14448/95 (SH L 608 R)	
Stabilität	wird nachgetragen	
Spezifische Radioaktivität	--	
Hersteller	Schering AG	
Menge an Arzneistoff pro Einheit	270 mg ZK 200775/54 ml vials	
Zusammensetzung des Trägers	Zusammensetzung der Rekonstitutionslösung (SH L 608 R): 250,00 mg Natriumchlorid + 302,5 mg Trometamol + 17,8 mg HCl 10%ig ad 50,0ml Wasser für Injektionszwecke	
Art der Verpackung und Einheiten pro Verpackung	50 ml-Vials, die mit 54 ml SH L 608 R befüllt werden	

Bemerkungen

Die Prüfpräparate werden von der Pharmazeutischen Entwicklung geliefert und sind gemäß § 10 AMG beschriftet.

Die Prüfpräparate werden an einem sicheren Ort bei Zimmertemperatur aufbewahrt.

Fly Sheet 4: Pharmazeutische Daten (Fortsetzung)

Daten zu Vergleichspräparat(en) und Formulierung(en)		
**Vergleichspräparat(e)**		
ZK Nummer	--	
Freiname	Natriumchloridlösung	
Chargennummer	FE 1212	
Analysennummer	9507573/05	
Stabilität	05/2000	
Hersteller	Fresenius	
**Formulierung(en)**		
Art der Formulierung	Infusionslösung	
SH Nummer	übergeordnete SH-Nr.: SH Y 608 ZY	
Handelsname	Isotonische Kochsalzlösung	
Chargennummer	D 2891	
Analysennummer	9507573/05	
Stabilität	05/2000	
Hersteller	Fresenius	
Spezifische Radioaktivität	keine	
Menge an Arzneistoff pro Einheit	0,9% NaCl	
Zusammensetzung des Trägers	--	
Art der Verpackung und Einheiten pro Verpackung		

Bemerkungen

Es wird nicht gegen die Rekonstitutionslösung SH L 608, sondern gegen eine isotonische Kochsalzlösung als Placebo verglichen.

Die Referenzpräparate sind Handelspräparate und werden gemäß § 10 AMG beschriftet.

Die Referenzpräparate werden an einem sicheren Ort bei Zimmertemperatur aufbewahrt.

Fly Sheet 5: Chemische Daten

Chemischer Name (IUPAC):	
7-Morpholino-2,3-dioxo-6-trifluoromethyl-1,2,3,4-tetrahydroquinoxaline-1-methylphosphonic acid	
ZK Nummer: 200775	Freiname: Quinophon

Strukturformel:

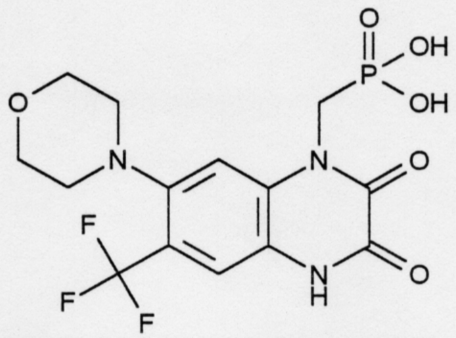

Inhaltsverzeichnis

1 Einleitung 8
2 Prüfziele 10
3 Untersuchungsplanung 10
3.1 Prüfdesign 10
3.2 Ethik 11
3.3 Zielpopulation 11
3.4 Behandlungen 16
3.5 Wirksamkeitskriterien 17
3.6 Verträglichkeits- und Wirkungsvariablen 17
3.7 Prüfungsablauf und Untersuchungszeitplan/Übersichtstabelle 21
3.8 Qualitätssicherung der Daten 24
3.9 Statistische Analysen 24
4 Teilnehmersicherheit 26
4.1 Belastungen/Risiken 26
4.2 Notfallregelungen 27
4.3 Nutzen-Risiko-Abwägung 27
4.4 Sperrfrist und Information des Teilnehmers über pathologische Befunde 27
5 Gesetzliche Grundlagen und GCP 28
5.1 Ethik-Kommission 28
5.2 Teilnehmerinformation 28
5.3 Teilnehmerversicherung 28
5.4 Dokumentations- und Archivierungshinweise 29
5.5 Verbrauch und Verbleib der Prüf- und Referenzpräparate 29
5.6 Qualitätskontrolle und -sicherung 29
Anhang 1: Beschreibung der Labormethoden 31
Anhang 2: KDH-Blocknamen 34
Anhang 3: Liste der Nebenwirkungen der Prüfung Nr. 95009 mit Graphiken der zeitlichen Verläufe 36

1 Einleitung

Der Schlaganfall ist die dritthäufigste Todesursache der über 60jährigen Menschen in den westlichen Industrienationen. Aufgrund der nach wie vor insuffizienten Therapiemöglichkeiten sterben 30 - 50 % der Betroffenen im Verlauf des Akutstadiums. Bis zu 70 % der einen Schlaganfall Überlebenden bleiben für den Rest ihres Lebens behindert.

Nach den heutigen pathophysiologischen und pathobiochemischen Erkenntnissen führt der ischämische Insult infolge arterieller Durchblutungsstörungen des Gehirns (85 % Auftrittshäufigkeit bei Schlaganfällen) zu einer Verminderung oder Unterbrechung der Versorgung des Hirngewebes mit Sauerstoff und Glukose. Sinkt der regionale Blutfluß unter 8 - 10 ml/100 g Gewebe/min., so wird das neuronale Gewebe irreversibel geschädigt (Infarzierungsschwelle) mit der Folge des neuronalen Zelltods. Im Verlauf der sog. zytotoxischen Kaskade spielt die Störung der Ionenhomöostase, und dort insbesondere die Freisetzung des exzitatorischen Neurotransmitters Glutamat, eine besondere Rolle. Durch Stimulierung der Glutamatrezeptoren, vornehmlich des NMDA-Rezeptors und des AMPA-Rezeptors, kommt es zu einem massiven Einstrom von Kalzium und Natrium in die Zelle. Die Folgen sind u.a. Zerstörung des Zytoskeletts, Schädigung der Zellmembran und Entstehung freier Radikale. Mit dem AMPA-Antagonisten ZK 200775 wird das Therapieprinzip verfolgt, durch Rezeptorbesetzung die zytotoxische Kaskade zu unterbrechen und neuroprotektive Wirkungen, besonders in der ischämischen Penumbranzone (Randzone) zu erzielen. ZK 200775 ist ein selektiver kompetitiver AMPA-Rezeptorantagonist, der spezifisch die ^{3}H-AMPA-Bindung hemmt und keine Affinität zu Nicht-Glutamatrezeptoren besitzt.

In Modellen der Neurodegeneration konnte gezeigt werden, daß ZK 200775 gegen den neuronalen Zellverlust durch globale Ischämie bei Gerbillus schützte und das Infarktvolumen bei fokaler Ischämie, die durch unilateralen Verschluß der Arteria cerebri medialis bei Ratten und Mäusen experimentell verursacht worden war, bis zu 35 % reduzierte. Das Zeitfenster für die Neuroprotektion betrug 4-6 Stunden nach Verschluß. Als zusätzliche pharmakologische Wirkungen von ZK 200775 bei Nagern fanden sich: Reduktion des Muskeltonus, Unterdrückung monosynaptischer Reflexe, Verminderung des Explorationsverhaltens, Verschlechterung der motorischen Koordination und Erhöhung der Schmerzschwelle.

Die erste Anwendung von ZK 200775 am gesunden älteren Mann diente der Überprüfung von Parametern der Sicherheit, der Verträglichkeit und der Kinetik. Es sollte festgestellt werden, ab welcher Dosisstufe und zu welchem Zeitpunkt Wirkungen auftreten (minimal effective dose - MED - Zeit-Wirkungs-Beziehungen). Innerhalb der vorgesehenen Dosisstufen von 0.015, 0.03, 0.075, 0.15, 0.3, 0.75 und 1.5 mg/kg/h ZK 200775, verabreicht als einmalige 4-stündige Infusion, sollte auch geklärt werden, wo die maximal verträgliche Dosis - MTD - beim gesunden Menschen liegt. Die Versuchsanordnung dieser prospektiven, randomisierten, pro Dosisstufe doppelblinden Prüfung war ein forced-dose-design mit jeweils einer Auswertungsphase zwischen den Dosierungsstufen. In jede Dosisstufe waren 6 Verumprobanden und 2 Placeboprobanden (physiologische Kochsalzlösung) im Alter von 55-65 Jahren eingeschlossen worden.

Die Ergebnisse der akuten Dosistitrationsprüfung lassen sich nach dem vorläufigen Stand der Auswertung wie folgt zusammenfassen:

- Die MED ist in den Dosisgruppen zwischen 0.03 einschließlich 0.15 mg/kg/h zu suchen. Je ein Proband hatte Sehstörungen in Dosierungen 0.03 und 0.15 mg/kg/h. Jeweils 3 Probanden auf den Dosisstufen 0.075 und 0.15 mg/kg/h wiesen zentralnervöse Begleitwirkungen auf, von den entsprechenden Placeboprobanden nur einer. Alle

Symptome waren von kurzer Dauer (Minuten bis wenige Stunden) und von geringer Intensität.

- In der Dosisstufe mit 0.3 mg/kg/h kam es zu einem markanten Zuwachs an Begleitwirkungen: 5 von 6 Verumprobanden zeigten Sehstörungen mit einer medianen Dauer von 4.6 h und zentralnervöse Symptome mit einer medianen Dauer von 0.8 h. Der Interpretation explorativer Statistiken der prä- minus post-Werte zufolge war im Balancetest eine verringerte Zahl der Null-Durchgänge festzustellen, d.h. der normale Schwankungsausgleich war gegenüber Placebo vermindert (reduzierte Reagibilität). Im EEG waren unter explorativ statistischen Gesichtspunkten Sedierungszeichen zu objektivieren (prozentuale Zunahme im Deltaband, Abnahme im Alphaband).
- Nach einer deutlichen Verstärkung des Wirkungsbildes in der Dosisstufe 0.75 mg/kg/h, bei der 8 Verumprobanden mit langanhaltenden Sehstörungen und zentralnervösen Symptomen belastet waren, wurde mit der Dosis 1.5 mg/kg/h die maximal verträgliche Dosis erreicht. Alle Probanden wiesen Sehstörungen mit einer medianen Dauer von 19,8 Stunden und ZNS-Symptome mit einer medianen Dauer von 28,8 Stunden auf. Zugleich erhöhte sich der Schweregrad der Symptome, der Beginn der Symptome verlagerte sich im Median von 2,1 Stunden (0.3 mg/kg/h) über eine Stunde (0.75 mg/kg/h) auf 0.4 Stunden (1,5 mg/kg/h) nach Start der Infusion. Zentralnervöse Symptome wurden in den Dosisstufen 0.75 und 1.5 mg/kg bis 48 Stunden, d.h. bis zum Ende der Beobachtungsperiode registriert.

Die von den Probanden selbst sehr eindringlich geschilderten optischen Phänomene umfaßten ein breites Spektrum von Sehstörungen (Lichtempfindlichkeit - Oberflächen gleißend oder sehr dunkel im Vergleich zum normalen Erinnerungsbild - Doppeltsehen, Farbfehlwahrnehmungen, bewegte Konturen, bewegte Schatten, verschwommenes Sehen und Akkomodationsstörungen). Zu den zentralnervösen Symptomen gehörten vornehmlich Müdigkeit, Sprachstörungen, Euphorie, Benommenheit, Drehschwindel, Koordinationsstörungen, Gangstörungen, Konfusion, Konzentrationsstörungen, Amnesie, verlangsamtes Reaktionsvermögen, Kritiklosigkeit, Agitation und Unruhe, Nervosität sowie Kopfschmerz. Das Spektrum der Begleitwirkungen ebenso wie die Anzahl der betroffenen Probanden nahmen deutlich in den Dosisstufen 0.3 - 1.5 mg/kg/h zu.

Gleichermaßen verdeutlichte sich das Wirkspektrum in den pharmakodynamischen Variablen von der Dosisstufe 0.75 zu 1.5 mg/kg/h, so daß auf der höchsten Stufe nach explorativ statistischer Inspektion im Vergleich zu Placebo mehr objektive Parameter zu früheren und späteren Zeitpunkten signifikant ausfielen. Besonders ausgeprägt waren die Sedierungszeichen im EEG (prozentuale Zunahme im Deltaband; prozentuale Abnahme im Alphaband), im Balancetest (weniger Nulldurchgänge, höhere Intensiätsgrade der Schwankungen, Erniedrigung der Abweichungsmaxima) und im Symbol-Zahlen-Test (verlängerte Durchführungsdauer, verringerte Anzahl der richtigen Zahlen).

In der 1.5 mg/kg/h Dosisgruppe sowie in allen vorhergehenden Dosisstufen waren keine Veränderungen in den Vitalparametern, im EKG und den Laborparametern sichtbar, die als Substanzwirkungen interpretiert werden können. Bis auf einen Probanden (Proband Nr.: 52, Dosisstufe 1.5 mg/kg/h, Differenz zu Baseline = -1°C) wurde keine Temperatursenkung, wie aus der Tierpharmakologie prognostiziert, beobachtet. Die febrile Temperatur eines Probanden (Nr.: 55, Dosisstufe 1.5 mg/kg/h) bis 24 Stunden nach Infusionsbeginn ist mit hoher Wahrscheinlichkeit auf eine Infektion zurückzuführen. Klinisch relevante Veränderungen der Atemfrequenz waren nicht festzustellen.

Die visuellen Analogskalen bezüglich Befindlichkeit und Schlafverhalten zeigten in der Dosisstufe 1.5 mg/kg/h erst ab 24-36 Stunden deutliche Veränderungen in Richtung reduziertem Befinden und vergrößertem Schlafbedürfnis an. Die unveränderten Mittelwerte in der Zeit während und nach der Infusion bis 24 Stunden p.a. deuten darauf hin, daß die Substanz zu einer gewissen Kritiklosigkeit gegenüber den Beeinträchtigungen von Begleitwirkungen führte.

Die Pharmakokinetikdaten zeigten eine lineare Dosisabhängigkeit der Plasmaspiegel (Konzentration am Ende der Infusion, AUC) bis zu einer Dosis von 0.75 mg/kg/h. Die Substanz wurde mit einer terminalen Halbwertzeit von ca. 2-2.5 h aus dem Plasma eliminiert. Die Berechnung der totalen Clearance und des scheinbaren Verteilungsvolumens ergab 5 bis 8 l/h bzw. 15 bis 25 l. Steady-state wurde während der 4-stündigen Infusion nicht erreicht. Gleichgewichtsbedingungen sind während einer Dauerinfusion erst nach 4 bis 5 Halbwertzeiten zu erwarten, d.h. für ZK 200775 nach 8 bis 10 h. In der höchsten Dosisgruppe (1.5 mg/kg/h) wurde eine Senkung der totalen Clearance beobachtet (4 bis 6 l/h), während das scheinbare Verteilungsvolumen unverändert blieb.

Die erste akute Dosistitrationsprüfung am gesunden Probanden erwies die Bioverfügbarkeit, Sicherheit und Verträglichkeit von ZK 200775 in den Dosisstufen zwischen 0.015 bis 1.5 mg/kg/h mit einer deutlichen Zunahme von zentralen und pheripheren Begleitwirkungen in allen Parametern, jedoch ohne klinisch relevante vitale Belastung der Teilnehmer.

Eine Liste prüfungsrelevanter Informationen, wie Forschungsberichte, historische und bibliographische Daten, befindet sich im Trial Master File.

2 Prüfziele

Aufgrund der Befunde mit ZK 200775 in der ersten Anwendung beim Menschen sollen in dieser Studie die ophthalmologischen Effekte durch geeignete Untersuchungstechniken erfaßt und quantifiziert werden. Die Wahl der Dosisstufe 0,3 mg ergab sich aus dem markanten Zuwachs an ophthalmologischen Nebenwirkungen, die sich in der Dosisstufe 0,75 mg/kg/h noch verstärkten.

3 Untersuchungsplanung

3.1 Prüfdesign

Das Design dieser randomisierten, doppel-blinden placebokontrollierten Studie ist so angelegt, daß sich in jeder der zwei Dosisgruppen jeweils 6-Verum-Teilnehmer und 3 Placebo-Teilnehmer befinden. Vor und nach 4-stündiger Infusion mit ZK 200775 sollen in der Klinik und Poliklinik für Augenheilkunde der Charité und des Vichow-Klinikums unter standardisierten Bedingungen geeignete Augenuntersuchungen zur Erfassung und Quantifizierung der ophthalmologischen Nebenwirkungen stattfinden.

3.2 Ethik

Ethische Betrachtungen werden in Kapitel 5 "Gesetzliche Grundlagen und GCP" beschrieben.

3.3 Zielpopulation

Die Teilnehmer werden durch ein Zufallsauswahlverfahren aus der Zentralen Probandendatei des Instituts für Klinische Pharmakologie entsprechend der folgenden Ein- und Ausschlußkriterien ausgewählt, danach angeschrieben oder angerufen.

3.3.1 Anzahl der Teilnehmer

Die Anzahl der Teilnehmer für die gesamte Studie beträgt N = 18. In jeder Dosisstufe werden 9 Teilnehmer partizipieren, 6 mit Verum und 3 mit Placebo. Ausfallende Teilnehmer werden nach Möglichkeit ersetzt (siehe auch Kapitel 3.9.5. " Details zur Berechnung des Stichprobenumfangs").

3.3.2 Einschlußkriterien

Die Erfüllung der Einschlußkriterien wird auf dem Prüfungs-Startbogen bestätigt.

Teilnehmer, die in die Prüfung eingeschlossen werden, müssen allen folgenden Kriterien entsprechen:

3.3.2.1 Allgemeine Einschlußkriterien

a Der Teilnehmer muß Proband sein.

b Das Alter soll mindestens 55 Jahre und höchstens 65 Jahre betragen.

c Das Körpergewicht soll folgenden Wert nicht über- oder unterschreiten: Körpergröße in cm minus 100 = Gewicht in kg ± 20%.

d Das Geschlecht der Teilnehmer soll männlich sein.

e Die Zugehörigkeit zu einer ethnischen Gruppe ist ohne Bedeutung.

f Der Teilnehmer muß sein schriftliches Einverständnis gegeben haben.

g Körperliche Untersuchung

Teilnehmer sollen Normalbefunde oder Befunde ohne klinische Relevanz aufweisen. Der Teinehmer soll körperlich und seelisch gesund sein, d.h. er sollte keine Abnormitäten mit klinischer Relevanz in allen untersuchten medizinischen Bereichen aufweisen. Insbesondere dürfen keine gastrointestinalen, leber- und nierenspezifischen, kardiovaskulären, hämatologischen, endokrinologischen und neurologisch-psychiatrischen Erkrankungen vorliegen.

3.3.2.2 Spezielle Einschlußkriterien

nicht zutreffend

3.3.3 Ausschlußkriterien

Die Prüfung der Ausschlußkriterien wird auf dem Prüfungs-Startbogen bestätigt.

Von der Prüfung sind Teilnehmer auszuschließen, sobald sie eines der folgenden Kriterien aufweisen:

3.3.3.1 Anamnese

a wesentliche, nicht vollständig ausgeheilte Vorerkrankungen, bei denen vermutet werden kann, daß die Absorption, Verteilung, Ausscheidung und Wirkung der zu untersuchenden Wirkstoffe nicht normal verläuft. Ferner dürfen keine klinisch relevanten Vorbefunde zu nicht ausgeheilten leber- und nierenspezifischen, kardiovaskulären, hämatologischen, endokrinologischen und neurologisch-psychiatrischen Erkrankungen vorliegen.

b bekannte allergische Reaktionen auf die verwendeten Wirkstoffe (z.B. Lokalanästhetikum zur Augeninnendruckmessung im Rahmen der opthalmologischen Voruntersuchung: Thilorbin mit dem Bestandteil Oxybuprocainhydrochlorid: Allergie auf örtl. Betäubungsmittel aus der Substanzgruppe der p-Aminobenzoesäureester) oder Bestandteile der galenischen Zubereitung.

3.3.3.2 Medikamenten- und Drogeneinnahmen

a Einnahme von systemisch oder örtlich wirkenden Medikamenten bzw. Stoffen, die dem Prüfziel entgegenstehen bzw. es beeinflussen könnten wie Psychopharmaka, insbesondere Antidepressiva, Barbiturate und Benzodiazepine.

b eine Anamnese, die auf Medikamenten- oder Alkoholmißbrauch hinweist

c der Genuß von mehr als 10 Zigaretten pro Tag

d Alkohol am Vortag

e extreme körperliche Belastungen durch Sport oder Arbeit innerhalb 8 Tagen vor der Prüfung

f Blutspenden in den vergangenen zwei Monaten

g relevante Impfungen und/oder Auslandsaufenthalte

h spezielle oder einseitige Ernährungsgewohnheiten, z.B. streng vegetarische oder unterkalorische Ernährung

i Sperrfrist aus anderen Prüfungen

3.3.3.3 Vital Signs (nach 3 min sitzend)

a Blutdruck: Hypertonie mit systolischen Werten > 160 mm Hg und/ oder diastolischen Werten > 95 mm Hg nach 3 Minuten in sitzender Position.

b Puls: Werte außerhalb von 50 - 100 Schlägen/Minute

3.3.3.4 EKG

a abnormales EKG (mit 12 Ableitungen)

3.3.3.5 Laborbefunde

a Hepatitis Antigen (HBsAG), Hepatitis C - Antikörper oder positiver HIV-Test

b positiver Drogenbefund

c klinisch relevante Abweichungen der untersuchten Parameter

3.3.3.6 Sonstige Ausschlußkriterien

Wenn nach Meinung des Leiters der klinischen Prüfung oder des Arztes, der die Einschlußuntersuchung durchführt, aus wissenschaftlichen Gründen, aus Gründen der Gewährleistung eines ordnungsgemäßen Ablaufs der Prüfung (Compliance) oder aus Gründen der Sicherheit des Teilnehmers eine Teilnahme an der Prüfung nicht erfolgen sollte.

3.3.3.7 Spezielle Ausschlußkriterien

- Teilnehmer sollen keine relevanten Trübungen der optischen Medien, Netzhauterkrankungen, Sehnerverkrankungen, Amblyopie oder Farbsinnstörungen aufweisen.
- Z.n. Intraocularchirurgie (Ausnahme Cat. ex. mit Hinterkammerlinse), Z.n. Laserkoagulation.
- Kurzsichtigkeit > -5 dpt, Weitsichtigkeit > +5 dpt
- Engwinkelglaukom

3.3.4 Einschlußuntersuchungen

Innerhalb von 8 Wochen vor Beginn der Prüfung wird jeder Teilnehmer ophthalmologisch, internistisch und laborchemisch untersucht. Die ophthalmologische Voruntersuchung wird in der Klinik und Poliklinik für Augenheilkunde der Charité und des Virchow Klinikums bei Herrn Dr. Rüther durchgeführt. Die Augenvoruntersuchung umfasst das Ausfüllen eines Anamnese- und Befundbogens, eine Visus- und Refraktionsbestimmung, sowie eine Spaltlampenbiomikroskopie, Funduskopie und eine Messung des Augeninnendrucks, um eventuelle ophthalmologische Vorerkrankungen auszuschließen.

Bei der laborchemischen Untersuchung werden Parameter zur Hämatologie (Leukozyten, Erythrozyten, Hämoglobin, Hämatokrit, MCH, MCV, Thrombozyten, neutrophile Granulozyten, eosinophile Granulozyten, basophile Granulozyten, Lymphozyten, Monozyten) zum Gerinnungsstatus (Prothrombinzeit, partielle Thromboplastinzeit) und zur klinischen Chemie untersucht (GPT, Gamma-GT, AP, Bilirubin, Kreatinin, Chlorid, Kalium, Natrium, Gesamtprotein, Albumin, Alpha1-Globulin, Alpha2-Globulin, Beta-Globulin, Gamma-Globulin, Ferritin, Urinstatus). (siehe Kapitel 3.7.1. "Methoden und zeitliche Zuordnung"). Die Laboruntersuchungen zur Vor- und Nachuntersuchung werden bei W&T durchgeführt.

Vor Prüfungsbeginn werden ein HIV- , ein HBsAG-Test und ein Hepatitis C - Antikörper - Test (die Teste dürfen nicht älter als 3 Monate sein) und ein Drogenscreening (Amphetamine, Barbiturate, Benzodiazepine, Cannabinoide, Cocain, Opiate, Methadon) durchgeführt.

Die Erfüllung der Ein- und Ausschlußkriterien wird auf dem Startbogen dokumentiert und der Teilnehmer als endgültig in die klinische Prüfung aufgenommen.

3.3.5 Abschlußuntersuchungen

Spätestens eine Woche nach der Infusion wird eine laborchemische Abschlußuntersuchung durchgeführt. Dabei werden Parameter zur Hämatologie, zum Gerinnungsstatus und zur klinischen Chemie untersucht (siehe Kapitel 3.7.1. "Methoden und zeitliche Zuordnung").

3.3.6 Behandlungsabbruch

3.3.6.1 Kriterien für den Abbruch der gesamten Prüfung

Schwerwiegende unerwünschte Ereignisse, die nach dem vorliegenden Erkenntnisstand durch die Prüfsubstanz ZK 200775 bedingt sein können und bei mehreren Teilnehmern auftreten, sowie wenn Kriterien erreicht werden, die für den Abbruch der Infusion im Kap. 3.3.6.3. definiert sind und bei mehr als 3 Probanden innerhalb einer der Dosisgruppen während einer Infusionsdauer < 30 Min. beobachtet werden, erfordern eine sofortige, eingehende Begutachtung durch den Leiter der klinischen Prüfung oder den behandelnden Arzt. Auf Basis dieser Begutachtung wird über den Abbruch der gesamten Prüfung entschieden.

3.3.6.2 Kriterien für den Abbruch bei einzelnen Teilnehmern

- Schwerwiegende unerwünschte Ereignisse, die nach dem vorliegenden Erkenntnisstand durch die Prüfpräparate bedingt sein können, erfordern eine sofortige, eingehende medizinische Begutachtung durch den Leiter der klinischen Prüfung. Auf Basis dieser Begutachtung wird über den Abbruch bei einem einzelnen Teilnehmer entschieden;
- während der Prüfung auftretende, behandlungsbedürftige Erkrankungen, die keine schwerwiegenden unerwünschten Ereignisse darstellen, die jedoch nach Meinung des Leiters der klinischen Prüfung dem Erreichen des Prüfziels entgegenstehen;
- wiederholt auftretende unerwüschte Ereignisse, deren Intensität mit "stark" bewertet wird;
- Laborbefunde, die nach ärztlichem Ermessen eine Erkrankung vermuten lassen
- Nichteinhaltung der Prüfungsbedingungen
- Zurücknahme des Einverständnisses

3.3.6.3. Kriterien für den Abbruch der Infusion

Die Infusion wird beendet, wenn folgende Bedingungen gegeben sind:

- Schwere, aus der vorhergehenden Prüfung unbekannte Sehstörungen
- Die geplanten ophthalmologischen Tests lassen sich nicht mehr durchführen.
- Befinden des Probanden:

1. Der Proband fühlt sich außerordentlich unwohl.
2. Der Proband ist apathisch mit deutlicher Vigilanzreduktion und mangelnder Weckreaktion.

3.3.7. Ausfallende oder die Prüfung abbrechende Teilnehmer und Compliance

Fällt ein Teilnehmer aus nicht prüfungsbedingten Gründen aus, wird er nach Möglichkeit ersetzt. Dabei erhalten nachrückende Teilnehmer aufsteigende Nummern beginnend mit 28. Die für den Ausfaller vorgesehene Behandlung geht auf den Nachfolger über. Der ausgefallene Teilnehmer wird im Forschungsbericht kasuistisch beschrieben. Seine Daten werden in einem separaten Dokumententyp aufgeführt.

Fällt ein Teilnehmer aus prüfungsbedingten Gründen aus (drop out), wird über den Ersatz nach Bewertung des Einzelfalles entschieden. Alle die Verträglichkeit betreffenden Daten werden in die Bewertung einbezogen, alle übrigen Daten werden in einem Appendix aufgeführt.

3.3.8 Blutentnahmen

Die während der gesamten Prüfung entnommene Blutmenge beträgt maximal 40 ml. Die Gesamtmenge setzt wie folgt zusammen: Vor- und Nachuntersuchung je 20 ml.

3.4 Behandlungen

3.4.1 Dosierung und Behandlungsschema

Die Behandlung je Dosisstufe bei 6 Teilnehmern besteht aus einer einmaligen Infusion von ZK 200775 über 4 Stunden. Die Dosierung der beiden Dosisstufen ist wie folgt vorgesehen: 0,3 und 0,75 mg/kg/h ZK 200775. Das Prüpräparat für die jeweilige Dosisstufe wird entsprechend der Herstellungsvorschrift hergestellt. Die Infusionslösung wird mittels eines Injektomaten (IVAC-4000) über eine Venenverweilkanüle (Braunüle, Braun Melsungen, 20G×33mm) infundiert. Als Kontrollbehandlung wird physiologische Kochsalzlösung eingesetzt.

Behandlung	Dosis/ Anwendungsart	Besondere Anwendungshinweise
Dosisgruppe 1 (6 Verum und 3 Placebo)	0,3 mg/kg/h Infusion über 4 Stunden	Placebo: 0,9% Natriumchloridlösung volumengleich
Dosisgruppe 2 (6 Verum und 3 Placebo)	0,75 mg/kg/h Infusion über 4 Stunden	Placebo: 0,9% Natriumchoridlösung volumengleich

3.4.2. Handhabung und Anwendungshinweise

Die Gabe des Prüfpräparates wird von einem prüfungsunbeteiligten Mitarbeiter vorgenommen. Der prüfungsunbeteiligte Mitarbeiter vergewissert sich und protokolliert, daß der Teilnehmer

die Präparate in der vorgesehenen Weise erhält. Treten Abweichungen vom Prüfprotokoll auf, so werden sie dokumentiert.

3.4.3 Randomisierung

Die Randomisierung folgt einem Versuch in unabhängigen Behandlungsgruppen mit Schichtung. Es gibt 2 Schichten, entsprechend den Dosisgruppen und in jeder Schicht gibt es 2 Behandlungen: ZK 200775 und Placebo. Die Verumgruppe umfaßt 6 Teilnehmer, die Placebogruppe 3 Teilnehmer je Dosisstufe. Insgesamt werden 18 Teilnehmer im Verhältnis 2:1 (12 ZK 200775, 6 Placebo) den Behandlungen zugeteilt. Die Randomisierungsliste wird von der Biometrie erstellt.

3.4.4 Verblindung

Um die Verblindung zu gewährleisten, wird ein prüfungsunabhängiges Team am Morgen vor der Infusion die Vorbereitung der Prüfpräparate und des Infusionssystems übernehmen. Das unabhängige Prüfteam erhält für seine Arbeit eine Randomliste. Eine weitere existiert in einem verschlossenen Umschlag im Trial Master File. Entsprechene Notfallbriefe werden im Institut für Klinische Pharmakologie und beim Prüfarzt gelagert.

3.4.5 Begleitmedikation

4 Wochen vor und während der Prüfung bis zur Nachuntersuchung ist Begleitmedikation nur nach Rücksprache mit dem Leiter der klinischen Prüfung erlaubt. Sollte während der Durchführung der klinischen Prüfung eine ärztliche Behandlung notwendig werden, wird im Einzelfall über die weitere Teilnahme entschieden.

3.5 Wirksamkeitskriterien

In dieser Prüfung werden keine Wirksamkeitskriterien erhoben.

3.6 Verträglichkeits- und Wirkungsvariablen

3.6.1 Untersuchungen zur Verträglichkeit

3.6.1.1 Laboruntersuchungen

Aufgrund von Kenntnissen über die Anwendung höhrerer Dosen werden Standard-Laborparameter zur klinischen Chemie, zur Hämatologie und zu Urinparametern außer zur Vor- und Nachuntersuchung nicht untersucht.

3.6.1.2 Weitere Untersuchungen zur Verträglichkeit

3.6.2 Pharmakodynamik

3.6.2.1. Ophthalmologische Untersuchung

- Augenmotilität, Pupillenreaktion
- Spaltlampenbiomikroskopie

- Funduskopie

Die ophthalmologische Untersuchung wird mit der 2-maligen Gabe von 1-2 Tropfen MYDRUM (Wirkstoff: Tropicamid) zur Weitstellung der rechten Pupille eingeleitet. Die erste Gabe erfolgt 20 Minuten, die zweite direkt vor der jeweiligen ophthalmologischen Untersuchung. Die Wirkstoffmenge beträgt etwa 0.25 -0.5 mg.

3.6.2.2. Psychophysische Verfahren

- Visusbestimmung (mit Nahlesevisus und Refraktionsbestimmung)
- Prüfung mit der Amsler-Karte
- Farbwahrnehmungsfähigkeitstest (Farnsworth Panel D 15 desat.):

  15 verschiedene Farbklötzchen sollen unter dem Aspekt der größtmöglichen

  Ähnlichkeit aneinandergereiht werden.
- Stereopsisprüfung
- Dunkeladaptation zur Messung der Stäbchenendschwelle (in einseitiger Mydriasis notwendig), Adaptometer nach Hartinger, Carl Zeiss Jena

3.6.2.3. Elektrophysiologische Verfahren

- Elektroretinogramm (Nicolet Biomedical)

  Helligkeits - ERG (standardmäßig nur am rechten Auge untersucht und dokumentiert)

  Nach vorheriger Dunkeladaption und in einseitiger Mydriasis (standardmäßig am rechten Auge) wird zur Ableitung der Netzhautpotentiale eine Fadenelektrode im unteren Bindehautsack (Dawson, Trick, Litzkow - Elektrode) eingesetzt. Zusätzlich erhält der Proband 3 Hautelektroden als Referenz und Erdung. Die eigentliche Untersuchung besteht aus kurzen Lichtblitzen mit ansteigender Intensität (max. 2.0 $cdsm^{-2}$), anschließender Helladaptation und erneuter Darbietung von Lichtblitzen (rot und weiß) sowie Flimmerreizung (Reizfrequenz 30 Hz), die dem vor einer Halbkugel sitzenden Probanden dargeboten werden. Anschließend Darbietung längerer, farbiger LED-Reize mit niedriger Lichtintensität.

  Muster - ERG (standardmäßig nur am linken Auge untersucht und dokumentiert)

  Beim Musterelektroretinogramm (MERG) wird mit gleicher Elektrodenplazierung (Fadenelektrode) statt eines Helligkeitsreizes zur Evozierung eines retinalen Potentials ein zeitlich modulierter Musterreiz (Schachbrettmusterwechselreize) verwendet mit einer Wechselfrequenz von 1.5 und 7.5 Hz, wobei die Gesamtleuchtdichte bei jeder Reizkonfiguraton konstant bleibt. Das MERG wird grundsätzlich bei Helladaption durchgeführt. Im Gegensatz zum Helligkeits-ERG wird an dem linken Auge mit "natürlicher" Pupille abgeleitet.

- Muster-VEP (visuell evozierte Potentiale)

  Beim Muster-VEP wird bei Schachbrettmusterwechselreizung des linken Auges mit "natürlicher" Pupille über dem okzipitalen Kortex abgeleitet. Es gestattet eine Prüfung der Nervenleitgeschwindigkeit im Nervus opticus und gibt Hinweise auf die Verarbeitung des visuellen Reizes in der primären Sehrinde. Es wird mit 2 Schachbrettmustergrößen (18 Winkelminuten und 1,2°) gereizt. Bei jeder Mustergröße wird in mindestens 2

Durchgängen 64-mal gemittelt. Dabei werden in einem Schachbrettmuster helle und dunkle Anteile in einer definierten Frequenz (1.5 Hz) vertauscht, wobei die globale Leuchtdichte konstant bleibt und lediglich die Bildstruktur verändert wird.

3.6.3 Unerwünschte Ereignisse

Unerwünschte Ereignisse sind alle im Rahmen einer klinischen Prüfung beobachteten Befindlichkeitsstörungen, subjektiven und objektiven Krankheitssymptome (einschließlich Laborwertveränderungen), interkurrente Krankheiten (und Unfälle), die nach dem Beginn der Prüfung auftreten oder sich verschlechtern. Dies gilt unabhängig von einem möglichen ursächlichen Zusammenhang mit der Gabe der Prüfsubstanz.

Unerwünschte Ereignisse sind immer als schwerwiegend einzustufen, wenn sie lebensbedrohlich sind bzw. zum Tode, zu einer dauerhaften wesentlichen Behinderung oder einem bösartigen Tumor oder zu einer stationären Behandlung führen bzw. diese verlängern.

Unerwünschte Ereignisse werden entweder vom Teilnehmer spontan berichtet oder von einem prüfungsbeteiligten Mitarbeiter beobachtet bzw. erfragt und dokumentiert. Unerwünschte Ereignisse werden auf dem CRF nach HARTS 2.3 (Hoechst adverse reaction terminology system, Version 2.3) codiert.

Schwere unerwünschte Ereignisse (serious adverse events) werden unabhängig von der üblichen Erfassung in einem nachgeschalteten Bearbeitungsvorgang auf dem SUE-Bogen dokumentiert und weitergeleitet.

Unerwünschte Ereignisse werden hinsichtlich der Intensität wie folgt bewertet:

1 = "leicht"	Das Symptom beeinträchtigt das normale Funktionsniveau des Teilnehmer nicht oder nur geringfügig.
2 = "mittel"	Das Symptom beeinträchtigt in gewissem Maße das normale Funktionsniveau des Teilnehmer.
3 = "stark"	Das Symptom bedeutet eine eindeutige, starke Beeinträchtigung des normalen Funktionsniveaus des Teilnehmers.

Die Beurteilung des Wahrscheinlichkeitsgrades eines ursächlichen Zusammenhangs der unerwünschten Ereignisse mit dem Prüfpräparat wird wie folgt bewertet:

Zusammenhang/Code	Definition
Keiner (1)	Die Zeitabfolge zwischen Gabe des Prüfpräparates und Auftreten bzw. Verschlechterung des UEs schließt einen ursächlichen Zusammenhang aus und/oder<br>eine andere Ursache ist gesichert und Hinweise auf eine (Mit)verursachung/Verschlechterung durch das Prüfpräparat liegen nicht vor.

Unwahrscheinlich (2)	Die Zeitabfolge zwischen Gabe des Prüfpräparates und Auftreten bzw. Verschlechterung des UEs macht einen ursächlichen Zusammenhang unwahrscheinlich<br>und/oder<br>die bekannten Wirkungen des Prüfpräparates bzw. der Substanzklasse geben keinen Hinweis auf eine (Mit)verursachung/Verschlechterung durch das Prüfpräparat und eine andere zur Erklärung hinreichende Ursache ist bekannt.<br>und/oder<br>aus den bekannten Wirkungen des Prüfpräparates bzw. der Substanzklasse läßt sich zwar eine plausible Kausalkette hinsichtlich einer (Mit)verursachung/Verschlechterung ableiten, eine andere Ursache ist jedoch wesentlich wahrscheinlicher<br>und/oder<br>eine andere Ursache ist gesichert und eine (Mit)verursachung/Verschlechterung des UEs durch das Prüfpräparat ist unwahrscheinlich.
Möglich (3)	Aus den pharmakologischen Eigenschaften des Prüfpräparates bzw. der Substanzklasse läßt sich eine plausible Kausalkette hinsichtlich einer (Mit)verursachung/Verschlechterung des UEs ableiten, andere etwa gleich wahrscheinliche Ursachen sind jedoch bekannt<br>oder<br>die pharmakologischen Eigenschaften des Prüfpräparates bzw. der Substanzklasse geben zwar keinen Hinweis auf eine (Mit)verursachung/Verschlechterung des UEs, eine andere zur Erklärung hinreichende Ursache ist jedoch nicht bekannt.
Wahrscheinlich (4)	Die pharmakologischen Eigenschaften des Prüfpäparates bzw. der Substanzklasse<br>und/oder<br>der Verlauf des UEs nach Absetzen des Prüfpräparates und einer ggf. folgenden Reexposition<br>und/oder<br>spezifische Befunde (z.B. positive Allergietestung bzw. Antikörper gegen Prüfpräparat/Metaboliten) legen eine (Mit)verursachung/Verschlechterung des UEs durch das Prüfpräparat nahe, eine andere Ursache kann jedoch nicht ausgeschlossen werden.
Gesichert (5)	Die pharmakologischen Eigenschaften des Prüfpräparates bzw. der Substanzklasse<br>und<br>der Verlauf des UEs nach Absetzen des Prüfpräparates und einer ggf. folgenden Reexposition<br>und<br>spezifische Befunde (z.B. positive Allergietestung bzw. Antikörper gegen Prüfpräparat/Metaboliten) sprechen ausnahmslos für eine (Mit)verursachung/Verschlechterung des UEs durch das Prüfpräparat und Hinweise auf andere Ursachen liegen nicht vor.

3.7 Prüfungsablauf und Untersuchungszeitplan/Übersichtstabelle

3.7.1 Methoden und zeitliche Zuordnung

Methoden und zeitliche Zuordnung

Periode		-1	1	1	1	1	1	1	1	9
**Relativzeit**	**Tag (dd)**	00	-00	-00	00	00	00	00	00	00
**Relativzeit**	**Std (hh)**	00	02	02	00	04	04	22	22	00
**Relativzeit**	**Min (mm)**	00	30	25	00	00	05	00	05	00
**Maßnahme**										
demographic data		X								
medical history		X								
physical examination		X								
vital signs		X								
quantitative ECG		X								
ECG diagnosis		X								
blood sample		X								X
drug screen		X								
adverse event									X	
concomitant medication									X	
end of study										X
drug administration					X					
ophthalmological history		X								
refraction measurement		X								
visual acuity		X	X	X		X	X	X	X	
Amsler-Card measurement		X	X	X		X	X	X	X	
ophthalmological examination		X	X	X		X	X	X	X	
examination of chromatopsia			X	X		X	X	X	X	
determination of dark adaptation			X	X		X	X	X	X	
electrophysiology: Flash ERG, Pattern-ERG, VEP			X	X		X	X	X	X	

Periode -1: Voruntersuchung

Periode 1-8: Hauptuntersuchung

Periode 9: Nachuntersuchung

Die Beobachtung erfolgt über den gesamten Zeitraum. Die Dokumentation ist dem Ende des Beobachtungszeitraum zugeordnet. Die Relativzeiten geben den Start der Untersuchungsserie an. Die Echtzeit der Einzeluntersuchung wird auf dem KDH-Bogen dokumentiert.

Die ophthalmologischen Untersuchungen (außer Examination of chromatopsia) werden für die Durchführung am linken bzw. rechten Auge zusätzlich mit dem Schlüssel localization geplant.

Für die Planung ergeben sich daraus 2 Relativzeiten im Abstand von 5 Minuten.

3.7.2. Praktischer Ablauf

3.7.2.1. Besonderheiten während der Einschlußphase

keine

3.7.2.2. Aufenthalt während der Prüfung

Die Durchführung des Behandlungsteils und der ophthalmologischen Untersuchungen findet in der Klinik für Augenheilkunde des Rudolf-Virchow-Klinikum statt. Zur Überwachung der Vor- und Nachphase bleiben die Probanden in den Untersuchungsräumen des Instituts für klinische Pharmakologie der Schering AG. Die Teilnehmer werden 1 Tag und 1 Nacht bei Schering verbringen (siehe auch 3.7.2.6 "Tagesablaufschema").

3.7.2.3. Teilnehmerverpflegung

Während des Aufenthalts bei Schering bekommen die Teilnehmer ein Standardessen.

Die Teilnehmer erhalten am Hauptprüftag um ca. 06.30 Uhr ein standardisiertes Frühstück (2 Brötchen, 100 g Wurst/ Käse, 25 g Marmelade, 40 g Butter, entkoffeinierten Kaffee) .

Um ca.13.00 Uhr wird ein Standard-Mittagessen (Tiefkühlkost, 1 Stück Obst und kohlensäurearmes Mineralwasser) gereicht.

Um ca. 16.30 Uhr erhalten die Teilnehmer "Kaffee" (1 Stück Kuchen, entkoffeinierten Kaffee).

Um 19.00 Uhr wird ein Abendbrot angeboten (3 Scheiben Brot, 20 g Butter, 150 g Wurst/Schinken/Käse, Tomaten/Gurken/Paprikascheiben, kohlensäurearmes Mineralwasser oder Früchte-Tee).

Das Essen an den Prüftagen muß von den Teilnehmer möglichst vollständig verzehrt werden und die Mindest-Trink-Menge sollte 1,5 Liter betragen. Während der Washout-Phase 24 h nach Start der Infusion können die Teilnehmer Normalkost essen.

3.7.2.4. Einschränkungen während der Prüfung

- Rauchen: nicht erlaubt
- Alkohol: nicht erlaubt
- Körperliche Aktivitäten: eingeschränkt

3.7.2.5. Aufbewahrung, Beschriftung usw. von biologischem Material

Entnommene Proben werden mit Prüfungs-Nr., Teilnehmer-Nr. und Relativzeitt beschriftet. Die nicht sofort analysierten Proben werden bei ca. -20 °C bis zur Aufarbeitung gelagert.

3.7.2.6. Tagesablaufschema

Tabelle 1: Tagesablaufschema

Period	rel. Zeit vor/nach Appl. [dd:hh:mm]	Uhrzeit	Maßnahme
-1	00:00:00		allgemeine und augenärztliche Voruntersuchung, Blutabnahme
-1	00:00:00	6.15	Proband erscheint im Institut für Klinische Pharmakologie,<br>Drogenscreening,<br>anschließend Frühstück und Fahrt in die Klinik und Poliklinik für Augenheilkunde der Charité und Virchow-Klinikums
1	-00:02:30	7.30	Psychophysische Augenuntersuchungen, elektrophysiologische Untersuchungen
1		9.30	Braunüle
1	00:00:00	10.00	Infusionsbeginn
1		13.00	Mittagessen
1	00:04:00	14.00	Ende der Infusion<br>Psychophysische Augenuntersuchungen, elektrophysiologische Untersuchungen,<br>Rückkehr ins Institut für Klinische Pharmakologie

Tabelle 1: Tagesablauf (Fortsetzung)

Period	rel. Zeit vor/nach Appl. [dd:hh:mm]	Uhrzeit	Maßnahme
1		16:30	Kaffee
1		19.00	Abendbrot, Übernachtung
1		7.00	Frühstück und Anfahrt in die Klinik und Poliklinik für Augenheilkunde der Charite´und Virchow-Klinikums
	00:22:00	8.00	Psychophysische Augenuntersuchungen, elektrophysiologische Untersuchungen, Entlassung, falls aus ärztlicher Sicht notwendig, weiterer Aufenthalt für 24 Stunden
9	00:00:00		Blutabnahme zur Nachuntersuchung

3.8 Qualitätssicherung der Daten

Maßnahmen zur Qualitätssicherung werden in Kapitel 5 "Gesetzliche Grundlagen und GCP" beschrieben.

3.9 Statistische Analysen

3.9.1 Ziel der Studie

Durch stufenweisen Erhöhen der Dosis von ZK 200775 soll entschieden werden, bis zu welcher Dosierung die Substanz bei Probanden als sicher und tolerierbar angesehen werden kann.

Dise Entscheidung wird sowohl nach jedem Dosisschritt als auch am Ende unter simultaner Bewertung aller Dosierungen und der Placebobehandlung getroffen.

3.9.2 Design

Es sind bis zu 2 Stufen vorgesehen. Beginnend mit der niedrigsten Dosis, kann diese einmal erhöht werden. Innerhalb jeder Stufe werden die Probanden der jeweiligen Dosisgruppe sowie Placebo im Verhältnis 6:3 zufällig zugeordnet.

Für die Gesamtbewertung werden alle mit Placebo behandelten Probanden zusammengefaßt. Daher entstehen K+1 unabhängige Gruppen. K ist der Index, der der höchsten tatsächlich applizierten Dosis entspricht. Die Studie ist innerhalb der Stufen doppelblind, nicht zwischen den Stufen.

3.9.3 Zwischenauswertungen

Nach jeder Stufe wird eine Bewertung aller sicherheitsrelevanten Aspekte vorgenommen. Tabellen, Grafiken und Listen werden erzeugt. Diese dienen der strukturierten Aufbereitung aller notwendigen Informationen für die medizinische Bewertung.

Zwischenergebnisse werden pro Dosisgruppe und Placebo sowie pro Meßzeitpunkt aufbereitet. Der Doppelblindcharakter wird im Hinblick auf die nächsten Diosisstufen beibehalten.

3.9.4 Analyse über alle Stufen

Ergebnisse werden pro (Dosis-) Gruppe einschließlich der gepoolten Placebobehandlung ((k+1) unabhängige Gruppen) und je Zeitpunkt aufbereitet; und zwar mit Hilfe deskriptiver statistischer Methoden. Für ausgewählte Variable erfolgt ggf. eine Verdichtung über mehrere Zeitpunkte bzw. Zeitintervalle.

Stichprobenparameter sind: n, Mittelwert, Standardabweichung, Minimum, Median, Maximum für Variable mit metrischer Skala (sowie entsprechenden Verteilungsannahmen), Häufigkeitkeiten / Prozente für Variable mit Nominal- oder Ordinalskalenniveau.

Vergleiche der Gruppen (global, paarweise Vergleiche) werden auf der Basis der folgenden Methoden durchgeführt:

Metrik der Variablen	Methode / Statistik
nominal	χ^2-test, Fishers exakte Methode
ordinal or diskrete Zählungen	Kruskal-Wallis Test, Wilcoxon Rank Summentest
metrisch	einfaktorielle Varianzanalysen, LSD Procedure
„time to event" Daten (gruppiert)	logrank test

P-Werte, die den Gruppendifferenzen entsprechen, werden nicht adjustiert. Dies korrespondiert zu dem explorativen Charakter und dem Design der Studie. (Dosiseinflüsse können nicht von Zeiteffekten getrennt werden.)

3.9.5 Details zur Berechnung des Stichprobenumfangs

Der Stichprobenumfang beruht nicht auf einer statistischen Rationale, die klinisch relevante Unterschiede heranzieht. Nur sehr große Unterschiede zwischen den Dosierungen und gegenüber Placebo könnten auf akzeptablen Niveaus für die Irrtumsrisiken entdeckt werden, wobei die Gruppenumfänge 6:6:6 betragen, wenn zwei Stufen durchgeführt werden können.

Die Entscheidung ggf. über den Abbruch der Studie nach oder während der ersten oder zweiten Stufe soll auf Expertenurteil beruhen. Ein hoher Fehler 1. Art zuungunsten der Testsubstanz wird dabei bewußt in Kauf genommen, da die Sicherheit den Vorrang hat.

Die o.g. Fallzahl soll erreicht werden, ggf. durch geeignetes Nachsetzen.

4 Teilnehmersicherheit

4.1 Belastungen/Risiken

4.1.1. Belastungen/Risiken aus der Prüfungsanordnung

Zur Vorbereitung auf die Applanationstonometrie im Rahmen der ophthalmologischen Voruntersuchung werden den Probanden einmalig jeweils 1 Tropfen Thilorbin als Oberflächenanästhetikum in die Augen eingetropft. Dadurch kann eine kurzeitige Beeinflussung der Hornhaut, die nach max. 30 Minuten abgeklungen ist, sowie ein leichtes Brennen auftreten. In seltenen Fällen kann es bei Probanden mit bestehender Allergie auf örtl. Betäubungsmittel aus der Substanzgruppe der p-Aminobenzoesäureester zu einer allergischen Reaktion auf den Bestandteil Oxybuprocainhydrochlorid kommen. Dieses Allergierisiko wird durch den Ausschluß von Probanden mit bekannter Überempfindlichkeit minimiert.

Belastungen/Risiken durch Anlage einer Verweilkanüle für Blutentnahmen und Infusion kann von Blutergüssen, in seltenen Fällen auch von vorübergehenden Entzündungen der Venenwand begleitet sein. Nach anfänglicher Irritation ist der Verbleib der Plastikkanülen in der Regel schmerzfrei und wird kaum wahrgenommen. Gleiches gilt für Einzelpunktionen zur Blutabnahme.

Der ca. 5-stündige Aufenthalt in sitzender Position kann für einzelne Teilnehmer durch evtl. auftretende Beschwerden, wie Rückenschmerzen belastend, jedoch nicht risikobeladen sein.

Die Untersuchung durch das Helligkeitselektroretinogramm geschieht in einseitiger Mydriasis. Die Mydriasis ist nach Ausschluß eines Engwinkelglaukoms praktisch ohne Gefahren. Sollte sich eine Augendruckerhöhung in Folge der Mydriasis einstellen, werden die Probanden unverzüglich augenärztlich behandelt; über entsprechende Symptome werden sie aufgeklärt. Die Probanden müssen außerdem aufgeklärt werden, daß gewisse Tätigkeiten, insbesondere Lesen, für einen bestimmten Zeitraum (meist 3-5 Stunden) nicht möglich sind. Die Einstellung der Mydriasis erfolgt durch 1-2 Tropfen MYDRUM (Wirkstoff Tropicamid).

Die Fadenelektroden beim Elektroretinogramm, die in den unteren Bindehautsack eingelegt werden, verursachen ein gewisses Fremdkörpergefühl mit eventuellem Augentränen. Eine Verletzung der Hornhaut ist nicht zu erwarten.

4.1.2 Belastungen/Risiken durch die Prüfpräparate

Die erste akute Dosistitrationsprüfung am gesunden ältern männlichen Probanden erwies die Bioverfügbarkeit, Sicherheit und Verträglichkeit von ZK 200775 in den Dosisstufen 0.015 - 1.5 mg/kg/h über eine 4-Stunden Infusion mit einer deutlichen Zunahme von visuellen, peripheren und zentralnervösen Begleitwirkungen sowie einer sich verstärkenden Sedierung in

pharmakodynamischen Parametern, jedoch ohne klinische relevante vitale Belastung der Teilnehmer. Die Einzelheiten können in Kapitel 1 Einleitung entnommen werden.

4.2 Notfallregelungen

4.2.1 Allgemeine Notfallregelungen

- Im Notfall wird der Leiter der klinischen Prüfung sofort hinzugezogen bzw. unterrichtet

4.2.2 Spezifische Notfallmaßnahmen

Die Behandlung unerwünschter Ereignisse soll zunächst symptomatisch erfolgen, im Bedarfsfall steht notfallmedizinisches Gerät zur Verfügung.

4.2.3 Administrative Regelungen

Schwerwiegende unerwünschte Ereignisse sind vom Leiter der klinischen Prüfung innerhalb der vorgeschriebenen Zeiträume dem Leiter des Instituts für Klinische Pharmakologie, dem Stufenplanbeauftragten und der zuständigen Ethik-Kommission zu melden. Dazu ist es erforderlich, alle schwerwiegenden unerwünschten Ereignisse eindeutig zu dokumentieren.

4.3 Nutzen-Risiko-Abwägung

Das Risiko der Prüfungsteilnehmer ist anhand der Daten aus der akuten Dosisfindungsprüfung über 4-Stunden-Infusion von 0.015 - 1,5 mg/kg/h mit den bekannten Resultaten zur Sicherheit, Verträglichkeit und Pharmakokinetik gut abzuschätzen. In der Erst-Studie mit ZK 200775 am Menschen wurden in den durchgeführten Dosisstufen keine irreversiblen Veränderungen oder lebensbedrohlichen Zustände beobachtet. Gefahren und Risiken sind als gering einzuschätzen. Eine Interaktion mit dem Mydriatikum MYDRUM 0,5% ist nicht zu erwarten. Die ophthalmologischen Untersuchungen sind seit Jahren klinische Routine und stellen nach dem heutigen Stand der Kenntnis keine Gefahren dar. Das Mydriatikum, MYDRUM (Tropicamid 0,5%), das für das Helligkeits-ERG benötigt wird, ist nach Ausschluß eines Engwinkel-Glaukoms praktisch ohne Gefahren.

Nach Abwägung von Nutzen und Risiko wird die Durchführung der Prüfung als ethisch vertretbar eingestuft, weil der erwartete spätere therapeutische Nutzen durch das Prüfpräparat heute größer erscheint als die Risiken für die Teilnehmer. Der Prüfplan wird der zuständigen Ehtikkommission zur Begutachtung vorgelegt.

4.4 Sperrfrist und Information des Teilnehmers über pathologische Befunde

Die Sperrfrist nach individuellem Prüfungsende beträgt 2 Monate.

Sperrfristen, die aufgrund der Teilnahme an klinischen Prüfungen des Instituts für Klinische Pharmakologie oder der Teilnahme an anderen Prüfungen, die durch andere Arzneimittelprüfungs-Institute oder andere Abteilungen der Schering AG gesetzt wurden, müssen eingehalten werden.

Werden während der Voruntersuchung, während der Durchführung der klinischen Studie oder während der Nachuntersuchung Befunde mit Krankheitswert gefunden, wird der Teilnehmer informiert und aufgefordert, mit seinem Hausarzt Kontakt aufzunehmen.

5 Gesetzliche Grundlagen und GCP

Die Planung und Durchführung dieser klinischen Prüfung unterliegen den Bestimmungen der §§ 40 und 41 des deutschen Arzneimittelgesetzes (AMG). Die Empfehlungen der Deklaration von Helsinki (i.d.F. von Hongkong, 1989) und die europäischen GCP-Richtlinien vom 11.7.1990 werden beachtet.

5.1 Ethik-Kommission

Der Prüfplan wird der zuständigen Ethik-Kommission vorgelegt.

5.2 Teilnehmerinformation

Jeder Teilnehmer ist durch die Allgemeine Probandeninformation schriftlich und mündlich durch einen Arzt über die Rahmenbedingungen einer klinischen Prüfung informiert worden. Darüber hinaus wird jeder Teilnehmer mündlich durch einen Arzt über die Prüfung aufgeklärt. Er erhält ein Exemplar der schriftlichen Speziellen Probandeninformation und ein Exemplar seiner unterschriebenen Einverständnis Erklärung. Auf Wunsch kann ihm auch noch einmal eine aktuelle Allgemeine Probandeninformation ausgehändigt werden.

Der Teilnehmer ist darüber informiert worden, daß seine Daten nach Anonymisierung elektronisch gespeichert und zum Zwecke der wissenschaftlichen Auswertung verarbeitet werden. Er erklärt mit seiner Unterschrift, daß er damit einverstanden ist, daß autorisierte Personen der Schering AG oder der zuständigen (inländischen oder auch ausländischen) Behörden zur Überprüfung der ordnungsgemäßen Durchführung der klinischen Prüfung Einblick in die beim Leiter der klinischen Prüfung vorhandenen personenbezogenen Daten nehmen. Eine Weitergabe dieser Daten erfolgt - neben den gesetzlich geregelten Fällen der Weitergabe von Krankheitsdaten - nur zu Zwecken der Prüfung und nur in anonymisierter Form. Die Bestimmungen des Datenschutzgesetzes werden beachtet.

5.3 Teilnehmerversicherung

Jeder Teilnehmer ist gemäß § 40 Abs. 3 des AMG gegen Gesundheitsschädigungen versichert, die bei der Durchführung der Prüfung auftreten können.

Hiervon ausgeschlossen sind jedoch Gesundheitsschädigungen und Verschlimmerungen bereits bestehender Krankheiten, die auch dann eingetreten wären oder fortbestünden, wenn der Teilnehmer nicht an der klinischen Prüfung teilgenommen hätte.

Der Versicherungsschutz wird gefährdet, wenn der Teilnehmer sich während der Dauer der klinischen Prüfung - soweit sie den einzelnen Teilnehmern betrifft - einer anderen medizinischen Behandlung ohne Zustimmung des Leiters der klinischen Prüfung oder des Prüfarztes unterzieht oder eine Gesundheitsschädigung, die als Folge der klinischen Prüfung eingetreten sein könnte, nicht unverzüglich anzeigt.

Die Versicherungsbestimmungen sehen vor, daß eine Gesundheitsschädigung dem Versicherer anzuzeigen ist. In diesem Fall genügt es, wenn der Teilnehmer die Anzeige an die Schering Versicherungs-Vermittlung GmbH, Müllerstraße 163b, 13342 Berlin, richtet, die für die Weiterleitung an die Versicherungsgesellschaft sorgen wird.

Der Teilnehmer kann die Anzeige aber auch dem Prüfarzt oder dem Leiter der klinischen Prüfung erstatten, da diese auch zur Weitergabe verpflichtet sind.

Die Teilnehmerversicherung wird von der Schering AG - bei IPM Netzplanbüro (zur Weiterleitung an die Schering Versicherungs-Vermittlung GmbH) - auf Basis des "Antrages auf Durchführung einer klinischen Prüfung" (Teilnehmeranzahl) veranlaßt. Der Versicherer ist der Gerling-Konzern-Allgemeine Versicherungs-Aktiengesellschaft, Postfach 100808, 50448 Köln; die Nummer der Versicherungspolice lautet 1/000/00/02/815 9717/16.

5.4 Dokumentations- und Archivierungshinweise

- Die Dokumentation wird nach den Vorgaben des Aktenplans geführt.
- Elektronische Datenträger werden eindeutig mit Prüfungs-Nr./Teilnehmer-Nr./Datenart/Angaben zur Hard- und Software (Lesbarkeit der Daten) gekennzeichnet.
- Prüfungsunterlagen, die in Papierform erstellt werden bzw. vorliegen, wie z.B. Rohdaten auf Datenformblättern, CRFs, formlose Blätter, Ausdrucke, o.ä., werden einheitlich mit Prüfungs-Nr./Teilnehmer-Nr./Meßzeitpunkt bzw. Uhrzeit/Datum/Kurzzeichen des prüfungsbeteiligten Mitarbeitern gekennzeichnet. Die CRF-Formblätter werden während des Aufenthaltes in der Klinik und Poliklinik für Augenheilkunde der Charité und des Virchow Klinikums von prüfungsbeteiligten Mitarbeitern ausgefüllt. Für die Dokumentation der Untersuchungen die jeweils nur an einem Auge geplant sind, werden auch KDH-Bögen für das jeweils kontralaterale Auge mitgeführt. Diese Bögen werden standardmäßig nicht, sondern nur im begründeten Bedarfsfalle ausgefüllt. Sie werden im Trial Master File als Leerbögen abgelegt.
- Von Daten, die sich während der Prüfung auf elektronischen Datenträgern befinden, sind für Archivierungszwecke Papierausdrucke mit Datum und Kurzzeichen des zuständigen prüfungsbeteiligten Mitarbeitern herzustellen.
- Alle Prüfplanänderungen werden nach Freigabe der Endfassung des Prüfplans auf separatem Vordruck mit Anlaß, Inhalt, Begründung, Konsequenz, Datum und Unterschrift des Leiters der klinischen Prüfung dokumentiert. Prüfungsbeteiligte Mitarbeiter werden darüber durch Verteilung einer Kopie unterrichtet.
- Abweichungen vom Prüfplan während der Prüfung werden mit Begründung und Angabe des Zeitpunktes dokumentiert.
- Nach Prüfungsende sind die Prüfungsunterlagen (Trial Master File) entsprechend dem Aktenplan an das TMF-Archiv zu übergeben.
- Die Aufbewahrungsfrist für archivierte Prüfungsunterlagen beträgt mindestens 15 Jahre. Im Falle einer Zulassung des Arzneimittels müssen die Unterlagen mindestens bis 5 Jahre nachdem das Arzneimittel vom Markt genommen wurde, aufbewahrt werden.

5.5 Verbrauch und Verbleib der Prüf- und Referenzpräparate

Der Verbrauch und Verbleib der Prüf- und Referenzpräparate wird dokumentiert. Nach Behandlungsende sind die Behältnisse incl. restlicher Prüf- und Referenzpräparate an die ZV-Lagerverwaltung zur Entsorgung zu übergeben. Über den Verbleib fehlender Prüf- und Referenzpräparate oder deren Behältnisse ist eine schriftliche Erklärung zu verfassen.

5.6 Qualitätskontrolle und -sicherung

Ein prüfungsbeteiligter Mitarbeiter wird die erhobenen Daten zum Zweck der Qualitätskontrolle auf formale Richtigkeit, Vollständigkeit und Lesbarkeit der Einträge kontrollieren.

Eine unabhängige Qualitätssicherungseinheit wird Audits des Prüfplans, der Dokumentation und der Durchführung der Prüfung sowie gegebenenfalls des Forschungsberichtes vornehmen, um sicherzustellen, daß die Prüfung in Übereinstimmung mit den GCP-Standards durchgeführt wird.

Alle prüfungsbeteiligten externen Labore und Untersuchungsstellen nehmen an Qualitätssicherungsmaßnahmen teil. Die Unterlagen zu den Ringversuchen und den ständigen Qualitätskontrollen der Auftragslabore werden zentral im Raum der Laborkoordination des Institutes für Klinische Pharmakologie archiviert.

Anhang 1: Beschreibung der Labormethoden

In den folgenden Labor-Übersichtstabellen werden zu den einzelnen Parametern Referenzwerte angegeben. Diese stellen Orientierungshilfen dar, da sie sich streng genommen nur auf einen morgens nüchternen Zustand beziehen und ihre Spannweite verschiedenen biologischen und analysentechnischen Variationen unterliegt. Im Fall des Überschreitens des Referenzbereiches eines Meßwerts ist dieses Ereignis stets im Zusammenhang mit Vor- und Nachwerten sowie im Vergleich zu einer Kontrollgruppe zu sehen.

1. Standard-Laborparameter (mit Referenzbereichen für Männer)

ITEM	NAME	reference ranges		UNITS	METHOD
		low	high		
		**Hematology**			
L70101	Leucocytes, EDTA-Blood	4,4	11,3	10^9/l	Cell-DYN system, Abbott
L70201	Erythrocytes, EDTA-Blood	4,5	5,9	10^12/l	Cell-DYN system, Abbott
L70301	Hemoglobin, EDTA-Blood	140	175	g/l	Cell-DYN system, Abbott
L70401	Hematocrit, EDTA-Blood	0,42	0,5	l/l	Cell-DYN system, Abbott
L70501	Mean corp. vol., EDTA-Blood	80	96	fl	Cell-DYN system, Abbott
L70601	MCH (HBE), EDTA-Blood	28	33	pg	Cell-DYN system, Abbott
L70701	Platelets, EDTA-Blood	140	392	10^9/l	Cell-DYN system, Abbott
L75801	Neutrophilis, EDTA-Blood	53	75	%	Cell-DYN system, Abbott
L76101	Eosinophils, EDTA-Blood	2	4	%	Cell-DYN system, Abbott
L76201	Basophilis, EDTA-Blood	0	1	%	Cell-DYN system, Abbott
L75901	Lymphocytes, EDTA-Blood	25	40	%	Cell-DYN system, Abbott
L76001	Monocytes, EDTA-Blood	0	12	%	Cell-DYN system, Abbott
		**Clotting status**			
L80101	Prothrombin-time (Quick), Citrat.	70	120	%	Electra, Baxter, clotting
L80301	Partial thromboplastin time (PTT), Citratplasma	24	35	seconds	Electra, Baxter, clotting
		**Serum Chemistry**			
L00601	GPT, Serum	0	41	U/l	DGKC, optimized 37°C, Hitachi
L00401	Gamma-GT, Serum	0	49	U/l	Meth. acc. to Szasz. 1974, 37°C, Hitachi
L00301	Alkaline phosphatase, Serum	0	270	U/l	DGKC, optimized 37°C, Hitachi
L10101	Total Bilirubin, Serum (DPD)	0	18,8	umol/l	DPD, Diazonium-React. to Azobilirubin, 37°C, Hitachi
L10501	Creatinine, Serum	74	110	umol/l	Acc. to Jaffe, Kinetically. without deproteination, 37°C, Hitachi
L30201	Chloride, Serum	97	108	mmol/l	coulometry
L30301	Potassium, Serum	3,6	4,8	mmol/l	Flame Photometry, IL 943, Standard: Cs
L30401	Sodium, Serum	135	144	mmol/l	Flame Photometry, IL 943, Standard: Cs
L11401	Total Protein, Serum	66	83	g/l	Biuret-Methode, 37°C. Hitachi
L21901	Albumin, Serum	55,3	68,9	%	Electrophoresis with Poinceau-red, LRE System
L22101	Alpha-1-Globulin, Serum	1,6	5,8	%	El'phoresis w. Poinceau-red, LRE System
L22201	Alpha-2-Globulin, Serum	5,9	11,1	%	El'phoresis w. Poinceau-red, LRE System
L22301	Beta-Globulin, Serum	7,9	13,9	%	El'phoresis w. Poinceau-red, LRE System
L22401	Gamma-Globulin, Serum	11,4	18,2	%	El'phoresis w. Poinceau-red, LRE System
L57301	Ferritin, Serum	35	217	ug/l	MEIA. Abbott

Urine Status (Dip Stick)					
ITEM	NAME	reference ranges		UNITS	METHOD
		low	high		
L60101	pH-values (Stix), Urine	4,6	8	-LOG10 [H+]	COMBUR STIX
L60205	Urobilinogen (Stix), Urine			Clasu	COMBUR STIX 1=negativ, 2=(+)schwach positiv, 3=+, 4=++, 5=+++, 6=++++
L60305	Erythrocytes (Stix), Urine			Clasu	COMBUR STIX 1=negativ, 2=(+)schwach positiv, 3=+, 4=++, 5=+++, 6=++++
L16405	Total Protein (Stix), Urine			Clasu	COMBUR STIX 1=negativ, 2=(+)schwach positiv, 3=+, 4=++, 5=+++, 6=++++
L60605	Ketone (Stix), Urine			Clasu	COMBUR STIX, 1=negativ, 2=(+)schwach positiv, 3=+, 4=++, 5=+++, 6=++++
L60705	Bilirubin (Stix), Urine			Clasu	COMBUR STIX, 1=negativ, 2=(+)schwach positiv, 3=+, 4=++, 5=+++, 6=++++
L60804	Nitrite (Stix), Urine			Clasu	COMBUR STIX, 1=negativ, 2=positiv
L47505	Glucose (Stix), Urine			Clasu	COMBUR STIX, 1=negativ, 2=(+)schwach positiv, 3=+, 4=++, 5=+++, 6=++++
**Urine Drug Screen**					
L62304	Amphetamines, Urine			Class	ENZYME-IMMUNO-ASSAY. 1=negativ, 2=positiv
L62404	Barbiturates, Urine			Class	EIA, 1=negativ, 2=positiv
L62504	Benzodiazepines, Urine			Class	EIA, 1=negativ, 2=positiv
L62604	Cannabinoids, Urine			Class	EIA, 1=negativ, 2=positiv
L62704	Cocaine, Urine			Class	EIA, 1=negativ, 2=positiv
L62804	Opiates, Urine			Class	EIA, 1=negativ, 2=positiv
L62904	Methadone, Urine			Class	EIA, 1=negativ, 2=positiv

Virology					
L95004	Hepatitis-B-S-Antigen, serum			Class	MEIA. Abbott. 1=negativ, 2=positiv
L95104	Anti-HIV 1+2 (3rd generation)			Class	MEIA, Abbott 1=negativ, 2=positiv
L95204	Hepatitis-C-antibody			Class	MEIA, Abbott 1=negativ, 2=positiv

Anhang 2: KDH-Blocknamen

KDH-Blocknamen zum Kapitel: 3.7.1: Methoden und zeitliche Zuordnung

	**KDH-Blocknummer**	**KDH-Blockname**
pre-examination	002201	DATE OF EXAMINATION
	000301	DEMOGRAPHIC DATA
	000304	INITIALS / FILE NO.
	001201<br>001202	SMOKING HISTORY
	001203	ALCOHOL / DIET
	500022	MEDICATION HISTORY
	500017	PHYSICAL EXAMINATION
	001301	VITAL SIGNS
	501603	HEMODYNAMICS AND QUANTITATIVE ECG
	001613	ECG DIAGNOSIS
	500019	MEDICAL HISTORY
	002105	BLOOD SAMPLE
Laboratory values (hematology,hemostasis, chemistry)<br>Urinanalyse	002050	LABORATORY EXAMINATION
Virology	002029	VIROLOGY
Urin drug screen	001108<br>002106	URINE DRUG SCREEN<br>URINE SAMPLE
Blood sample	002105	BLOOD SAMPLE
Ophthalmological history	500023	OPHTHALMOLOGICAL HISTORY
ophthalmological examination	500024	OPHTHALMOLOGICAL EXAMINATION
Visual acuity	500026	EXAMINATION OF VISUAL ACUITY
Refraction measurement	500025	MEASUREMENT OF REFRACTION
Amsler-Card measurement	500027	AMSLER-CARD EXAMINATION

Examination of chromatopsia	500028	EXAMINATION OF CHROMATOPSIA
Determination of dark adaptation	500031	DETERMINATION OF DARK ADAPTATION
Electrophysiology (ERG)	500029<br>500030	ELECTRORETINOGRAM<br>ASSESSMENT OF ELECTRORETINOGRAM
Drug administration	001407<br>001404<br>001406	APPLICATION OF STUDY MEDICATION<br>TOTAL AMOUNT OF STUDY MEDICATION<br>DATE OF LAST STUDY MEDICATION
Adverse event	000103<br>000107	ADVERSE EVENTS
Concomit. medication	001504<br>001502	CONCOMITANT MEDICATION
End of study	001402	END OF STUDY
	000702	Investigator
	000703	Treatment
	000000	Start page trial

Anhang 3: Liste der Nebenwirkungen der Prüfung Nr. 95009 mit Graphiken der zeitlichen Verläufe

DATA-LISTING OF ADVERSE EVENTS (AE TC2083)
PER BODY-SYSTEM, TREATMENT, DOSE, SUBJECT, AE CODE (NO), AE CODE (TERM),
EVENT (GERMAN), STARTTIME, DURATION, INTENSITY AND DRUG RELATION

BODY-SYSTEM	GROUP	SUB	AE-CODE NO	AE-CODE TERM	EVENT	START TIME	DURA-TION	INTEN-SITY	DRUG RELAT
BODY/BACK	PLACEBO	24	3885	PAIN BACK	HEXENSCHUß	118:00	47:30	MODE	NO
BODY/BACK	ZK0.015	6	3885	PAIN BACK	RUECKENSCHMERZEN VOM SITZEN	2:15	4:40	MILD	NO
BODY/BACK	ZK1.500	53	3885	PAIN BACK	KREUZSCHMERZEN	40:00	31:00	MILD	UNLI
BODY/GEN	PLACEBO	5	3875	PAIN	ELEKTRODENDRUCK AN DER STIRN	0:55	0:26	MILD	NO
BODY/GEN	PLACEBO	5	3875	PAIN	ELEKTRODENDRUCK AN DER STIRN	1:21	3:05	MODE	NO
BODY/GEN	PLACEBO	5	3875	PAIN	ELEKTRODENDRUCK AN DER STIRN	11:30	2:00	MILD	NO
BODY/GEN	PLACEBO	24	2032	FLU SYND	GRIPPEAEHNLICHE SYMPTOME	-68:30	259:45	MILD	NO
BODY/GEN	PLACEBO	31	3875	PAIN	SCHMERZEN AM KOPF DURCH ELEKTRODENDRUCK	-1:30	5:25	MILD	NO
BODY/GEN	PLACEBO	31	3875	PAIN	SCHMERZEN AM KOPF DURCH ELEKTRODENDRUCK	4:50	8:10	MILD	NO
BODY/GEN	PLACEBO	36	3875	PAIN	BRENNEN AM AFTER DURCH SONDE	7:30	0:00	MODE	NO
BODY/GEN	PLACEBO	51	3875	PAIN	DRUCK IM STIRNBEREICH	0:48	4:00	MILD	POSS
BODY/GEN	ZK0.075	20	2032	FLU SYND	GRIPPEAEHNLICHE SYMPTOME	26:20	84:00	MILD	NO
BODY/GEN	ZK0.150	29	3875	PAIN	DRUCKSCHMERZEN AM KOPF DURCH ELEKTRODEN	0:57	0:30	MILD	NO
BODY/GEN	ZK0.300	35	3875	PAIN	DRUCK AN DER STIRN BIS ZUM SCHEITEL DURCH ELEKTRODEN	0:45	1:12	MILD	NO
BODY/GEN	ZK0.750	42	3875	PAIN	KOPFDRUCK IM STIRN- UND SCHLÄFENBEREICH, BDS.	5:35	2:59	MILD	POSS
BODY/GEN	ZK0.750	45	3875	PAIN	ELEKTRODENDRUCK AN DER STIRN	1:55	1:05	MILD	NO
BODY/GEN	ZK0.750	45	195	ALLERG REACT	PFLASTERALLERGIE VON DEN EKG-KLEBEELEKTRODEN	28:00	20:00	MILD	POSS
BODY/GEN	ZK0.750	49	3919	PHOTOSENSITIVITY	DER RAUM ERSCHEINT DUNKLER.	0:55	1:13	MILD	POSS
BODY/GEN	ZK0.750	49	3919	PHOTOSENSITIVITY	DER RAUM ERSCHEINT DEUTLICH DUNKLER.	2:04	0:05	MODE	POSS
BODY/GEN	ZK1.500	55	990	CHILLS	FRÖSTELN	19:29	1:50	MODE	POSS
BODY/GEN	ZK1.500	55	995	CHILLS FEVER	SCHÜTTELFROST	20:14	1:05	MODE	POSS
BODY/GEN	ZK1.500	55	1945	FEVER	FIEBER	20:49	2:40	SEVE	POSS
BODY/GEN	ZK1.500	55	1945	FEVER	FIEBER	23:29	0:45	MODE	POSS
BODY/GEN	ZK1.500	55	540	ASTHENIA	MUSKELSCHWÄCHE	24:14	1:30	MILD	POSS
BODY/GEN	ZK1.500	55	1945	FEVER	FIEBER	24:14	0:45	MILD	POSS
BODY/GEN	ZK1.500	55	1945	FEVER	FIEBER	35:59	12:30	MILD	POSS
BODY/GEN	ZK1.500	55	2032	FLU SYND	ERKÄLTUNG OHNE FIEBER	95:29	120:00	MODE	POSS
CV/GEN	PLACEBO	48	925	CARDIOVASC DIS	KREISLAUFKOLLAPS NACH DEM EINLEGEN DER BRAUNÜLE	-1:38	0:03	MILD	NO
CV/GEN	ZK0.300	40	925	CARDIOVASC DIS	KREISLAUFKOLLAPS OHNE BEWUSSTSEINSVERLUST	-25:53	0:18	MODE	NO
CV/GEN	ZK1.500	53	925	CARDIOVASC DIS	KREISLAUFKOLLAPS OHNE BEWUßTSEINSVERLUST	-1:15	0:15	MILD	NO
CV/GEN	ZK1.500	77	925	CARDIOVASC DIS	KREISLAUFKOLLAPS OHNE BEWUßTSEINVERLUST	5:22	0:38	MODE	POSS
CV/VASC/BP	ZK0.150	28	2815	HYPOTENS	HYPOTONIE	2:35	0:08	MODE	UNLI
CV/VASC/BP	ZK1.500	55	2815	HYPOTENS	KREISLAUFSCHWÄCHE	24:14	0:15	MILD	POSS
DIG/AN	PLACEBO	22	3882	PAIN ANUS	SCHMERZEN AM ANUS	-26:15	62:00	MODE	NO
DIG/AN	PLACEBO	22	3882	PAIN ANUS	SCHMERZEN AM ANUS	35:45	34:00	MILD	NO
DIG/AN	PLACEBO	36	3882	PAIN ANUS	BRENNEN AM AFTER DURCH SONDE	7:30	40:20	MODE	NO
DIG/AN	ZK0.075	21	3882	PAIN ANUS	SCHMERZEN AM ANUS	-1:30	47:30	MILD	NO
DIG/AN	ZK0.075	75	3882	PAIN ANUS	SCHMERZEN IM AFTER DURCH TEMPERATURSONDEN	-0:05	63:15	MILD	NO
DIG/BUC	ZK1.500	55	2110	GINGIVITIS	GINGIVITIS RE. UNTEN	-25:31	25:00	MODE	NO
DIG/BUC	ZK1.500	55	2110	GINGIVITIS	GINGIVITIS RE. UNTEN	-0:31	216:00	MILD	NO
DIG/BUC	ZK1.500	55	2110	GINGIVITIS	GERÖTETE AUGEN	4:08	0:00	MILD	POSS
DIG/BUC	ZK1.500	55	5057	TONGUE DIS	ZUNGE LEICHT WEIßLICH BELEGT	22:19	25:40	MILD	POSS
DIG/ESOPH	ZK1.500	56	1540	DYSPHAGIA	SCHLUCKBESCHWERDEN	28:20	0:05	MILD	POSS

DATA-LISTING OF ADVERSE EVENTS (AE TC2083)
PER BODY-SYSTEM, TREATMENT, DOSE, SUBJECT, AE CODE (NO), AE CODE (TERM),
EVENT (GERMAN), STARTTIME, DURATION, INTENSITY AND DRUG RELATION

BODY-SYSTEM	GROUP	SUB	AE-CODE NO	AE-CODE TERM	EVENT	START TIME	DURATION	INTENSITY	DRUG RELAT
DIG/GEN	PLACEBO	5	2100	GI DIS	SODBRENNEN	9:50	1:10	MILD	NO
DIG/GEN	ZK0.075	20	3535	NAUSEA	UEBELKEIT	26:20	84:00	MILD	NO
DIG/GEN	ZK0.150	28	3535	NAUSEA	ÜBELKEIT	-1:15	0:03	MILD	NO
DIG/GEN	ZK0.150	28	3535	NAUSEA	ÜBELKEIT	2:35	0:08	MILD	UNLI
DIG/GEN	ZK0.750	42	3535	NAUSEA	ÜBELKEIT	4:30	8:30	MILD	POSS
DIG/GEN	ZK1.500	55	3535	NAUSEA	ÜBELKEIT	24:14	0:15	MILD	POSS
DIG/GEN	ZK1.500	56	2100	GI DIS	FLAUES GEFÜHL IM MAGEN	1:25	1:05	MILD	POSS
DIG/GEN	ZK1.500	77	3535	NAUSEA	ÜBELKEIT BEIM AUFSTEHEN	5:19	2:11	MODE	POSS
DIG/OROP	ZK1.500	56	3944	PAIN THROAT	KRATZEN IM HALS	13:45	11:00	MILD	POSS
DIG/REC	ZK0.030	15	2482	HEMORRHOIDS	HÄMORRHOIDENBESCHWERDEN	5:42	45:00	MILD	NO
DIG/REC	ZK0.075	19	2482	HEMORRHOIDS	HÄMORRHOIDENBESCHWERDEN	13:50	46:13	MODE	NO
DIG/REC	ZK0.075	19	2482	HEMORRHOIDS	HÄMORRHOIDENBESCHWERDEN	60:03	63:47	MILD	NO
DIG/REC	ZK0.075	20	2415	HEM RECTAL	BLUTUNG AM AFTER DURCH TEMPERATURSONDE	47:45	62:35	MILD	NO
IJCN	ZK0.075	20	2945	INFLAM INJECT SITE	SCHMERZEN AN DEN EINSTICHSTELLEN DER BRAUNÜLEN	38:20	72:00	MODE	NO
IJCN	ZK0.075	20	3925	PAIN INJECT SITE	SCHMERZEN AN DER EINSTICHSTELLE DER BRAUNÜLE	38:20	0:00	MILD	NO
MAN/WB	ZK0.750	42	4940	THIRST	DURSTGEFUEHL	7:50	0:58	MODE	POSS
MAN/WB	ZK0.750	42	4940	THIRST	DURSTGEFUEHL	8:48	14:27	MILD	POSS
MAN/WB	ZK1.500	55	4940	THIRST	DURST	20:34	12:55	MODE	POSS
NER/ANS/SYM/L	PLACEBO	76	4835	SWEAT	NACHTSCHWEISS	15:54	6:15	MILD	POSS
NER/ANS/SYM/L	ZK0.300	40	4835	SWEAT	NACHTSCHWEISS	-10:05	6:00	MODE	NO
NER/ANS/SYM/L	ZK0.300	40	4835	SWEAT	NACHTSCHWEISS	13:40	8:15	MODE	UNLI
NER/ANS/SYM/L	ZK0.750	47	4835	SWEAT	SCHWITZEN	15:50	6:15	MODE	POSS
NER/ANS/SYM/L	ZK0.750	50	4835	SWEAT	NACHTSCHWEISS	15:55	7:35	MILD	POSS
NER/ANS/SYM/L	ZK1.500	55	4835	SWEAT	SCHWEIßAUSBRUCH	12:58	20:31	MODE	POSS
NER/ANS/SYM/L	ZK1.500	55	4835	SWEAT	NACHTSCHWEIß	109:29	34:00	MODE	POSS
NER/ANS/SYM/L	ZK1.500	77	4835	SWEAT	SCHWEIßAUSBRUCH	8:40	0:10	MODE	POSS
NER/CNS/B	PLACEBO	3	4597	SEDATION	MÜDIGKEIT	0:11	0:01	MILD	NO
NER/CNS/B	PLACEBO	10	430	ANXIETY	UNSICHERHEIT	0:55	1:20	MILD	POSS
NER/CNS/B	PLACEBO	10	1105	CNS DEPRESS	REAKTIONSVERMOEGEN, VERLANGSAMTES	0:55	1:20	MILD	POSS
NER/CNS/B	PLACEBO	10	4597	SEDATION	MUEDIGKEIT	0:55	1:27	MILD	POSS
NER/CNS/B	PLACEBO	10	4597	SEDATION	MUEDIGKEIT	10:55	11:30	MILD	UNLI
NER/CNS/B	PLACEBO	24	4597	SEDATION	MUEDIGKEIT	0:50	0:10	MILD	UNLI
NER/CNS/B	PLACEBO	24	4597	SEDATION	MÜDIGKEIT	1:35	0:20	MILD	UNLI
NER/CNS/B	PLACEBO	24	4597	SEDATION	MÜDIGKEIT	2:20	0:30	MILD	UNLI
NER/CNS/B	PLACEBO	31	4597	SEDATION	MUEDIGKEIT	5:15	2:30	MODE	POSS
NER/CNS/B	PLACEBO	31	4597	SEDATION	MUEDIGKEIT	7:45	5:15	MILD	POSS
NER/CNS/B	PLACEBO	32	4597	SEDATION	MÜDIGKEIT	1:55	2:30	MILD	POSS
NER/CNS/B	PLACEBO	51	4597	SEDATION	MÜDIGKEIT	0:48	7:50	MILD	POSS
NER/CNS/B	PLACEBO	51	1470	DIZZINESS	BENOMMENHEIT	1:00	3:48	MILD	POSS
NER/CNS/B	ZK0.015	4	4597	SEDATION	MÜDIGKEIT	2:55	1:00	MILD	UNLI
NER/CNS/B	ZK0.015	6	1470	DIZZINESS	BENOMMENHEIT	27:40	5:15	MILD	NO
NER/CNS/B	ZK0.030	15	4597	SEDATION	MÜDIGKEIT	2:02	4:00	MILD	POSS
NER/CNS/B	ZK0.075	17	4597	SEDATION	MUEDIGKEIT	9:00	4:45	MILD	UNLI
NER/CNS/B	ZK0.075	19	4597	SEDATION	MÜDIGKEIT	2:45	0:10	MILD	POSS
NER/CNS/B	ZK0.075	75	4597	SEDATION	MÜDIGKEIT	1:40	1:15	MILD	UNLI
NER/CNS/B	ZK0.075	75	4597	SEDATION	MÜDIGKEIT	3:00	2:10	MILD	UNLI
NER/CNS/B	ZK0.150	28	1470	DIZZINESS	SCHWINDEL	2:35	0:08	MILD	UNLI
NER/CNS/B	ZK0.150	29	1470	DIZZINESS	SCHWINDEL	5:38	0:08	MODE	POSS
NER/CNS/B	ZK0.150	30	4597	SEDATION	MUEDIGKEIT	1:45	2:00	MODE	POSS
NER/CNS/B	ZK0.150	30	4597	SEDATION	MUEDIGKEIT	3:45	2:15	MILD	POSS

DATA-LISTING OF ADVERSE EVENTS (AE TC2083)
PER BODY-SYSTEM, TREATMENT, DOSE, SUBJECT, AE CODE (NO), AE CODE (TERM),
EVENT (GERMAN), STARTTIME, DURATION, INTENSITY AND DRUG RELATION

BODY-SYSTEM	GROUP	SUB	AE-CODE NO	AE-CODE TERM	EVENT	START TIME	DURATION	INTENSITY	DRUG RELAT
NER/CNS/B	ZK0.300	33	4685	SPEECH DIS	SPRACHE VERLANGSAMT	5:37	0:15	MILD	POSS
NER/CNS/B	ZK0.300	34	4597	SEDATION	MUEDIGKEIT	1:25	0:30	MILD	POSS
NER/CNS/B	ZK0.300	34	4597	SEDATION	MUEDIGKEIT	2:40	0:15	MODE	POSS
NER/CNS/B	ZK0.300	35	4597	SEDATION	MUEDIGKEIT	3:23	0:07	MODE	POSS
NER/CNS/B	ZK0.300	35	4597	SEDATION	MUEDIGKEIT	4:48	0:05	MODE	POSS
NER/CNS/B	ZK0.300	35	4597	SEDATION	MUEDIGKEIT	8:15	0:45	MODE	POSS
NER/CNS/B	ZK0.300	38	4597	SEDATION	MUEDIGKEIT	2:05	7:25	MILD	POSS
NER/CNS/B	ZK0.300	38	1470	DIZZINESS	SCHWINDEL	5:15	2:40	MILD	POSS
NER/CNS/B	ZK0.300	39	1105	CNS DEPRESS	LEICHT VERLANGSAMTES REAKTIONSVERMÖGEN	1:26	5:00	MILD	POSS
NER/CNS/B	ZK0.300	39	1177	CONCENTRATION ABNORM	LEICHTE KONZENTRATIONSSTÖRUNGEN	1:26	5:00	MILD	POSS
NER/CNS/B	ZK0.750	42	4597	SEDATION	MÜDIGKEIT	3:20	0:10	MODE	POSS
NER/CNS/B	ZK0.750	42	1470	DIZZINESS	SCHWINDEL MIT DUNKLEN PUNKTEN V.D. AUGEN	5:35	2:25	MILD	POSS
NER/CNS/B	ZK0.750	42	4597	SEDATION	MATTIGKEITSGEFÜHL	5:35	5:25	MILD	POSS
NER/CNS/B	ZK0.750	42	215	AMNESIA	GEDÄCHTNISLÜCKEN	7:30	20:00	MILD	POSS
NER/CNS/B	ZK0.750	42	215	AMNESIA	PB. ERINNERTE SICH NICHT AN DIE FARBFEHLWAHRNEHMUNG	8:20	0:05	MILD	POSS
NER/CNS/B	ZK0.750	42	4597	SEDATION	SCHLAEFRIGKEIT	12:05	0:10	MODE	POSS
NER/CNS/B	ZK0.750	42	4597	SEDATION	SCHLAEFRIGKEIT	12:35	0:15	MODE	POSS
NER/CNS/B	ZK0.750	42	1470	DIZZINESS	SCHWINDEL	23:00	4:30	MILD	POSS
NER/CNS/B	ZK0.750	42	1105	CNS DEPRESS	VERLANGSAMTES REAKTIONSVERMÖGEN	25:00	2:30	MILD	POSS
NER/CNS/B	ZK0.750	42	4935	THINKING ABNORM	VERLANGSAMTES DENKEN	25:00	2:30	MILD	POSS
NER/CNS/B	ZK0.750	43	4597	SEDATION	MÜDIGKEIT, BEI RT AB 3, 5'KEINE REAKTION NUR BEI LAUTEM RUFEN	8:30	2:00	MILD	POSS
NER/CNS/B	ZK0.750	44	4597	SEDATION	MUEDIGKEIT,EINSCHLAF.BEI RT NACH 2,5MIN,CA 15XANSPRECHEN BEI EO	8:15	0:18	MODE	POSS
NER/CNS/B	ZK0.750	45	1470	DIZZINESS	GEFÜHL:"WIE IM ALKOHOLRAUSCH"	0:35	7:55	MILD	POSS
NER/CNS/B	ZK0.750	45	1105	CNS DEPRESS	VERLANGSAMTE REAKTIONEN, Z.B. BEIM UMSTECKEN ODER SYMBOLZAHLENT	1:10	6:50	MILD	POSS
NER/CNS/B	ZK0.750	45	215	AMNESIA	"FILMRIß", KEINE ERINNERUNG AN ERHALT SEINER ARMBANDUHR	5:30	0:05	SEVE	POSS
NER/CNS/B	ZK0.750	45	215	AMNESIA	VERGESSLICHKEIT	6:00	42:00	MILD	POSS
NER/CNS/B	ZK0.750	46	4597	SEDATION	MÜDIGKEIT	2:30	3:00	MILD	POSS
NER/CNS/B	ZK0.750	46	140	AGITATION	UNRUHE	8:04	4:56	MILD	POSS
NER/CNS/B	ZK0.750	46	1177	CONCENTRATION ABNORM	KONZENTRATIONSSTOERUNG WAEHREND DES UMSTECKTESTS	8:14	0:06	MILD	POSS
NER/CNS/B	ZK0.750	46	4597	SEDATION	MUEDIGKEIT WAEHREND DES EEG	8:30	0:15	MODE	POSS
NER/CNS/B	ZK0.750	46	4597	SEDATION	MUEDIGKEIT WAEHREND DES EEG	12:25	0:20	MODE	POSS
NER/CNS/B	ZK0.750	46	1180	CONFUS	VERWIRRTHEIT, PASSAGER, NACH DEM WECKEN	15:53	0:04	MILD	POSS
NER/CNS/B	ZK0.750	47	1470	DIZZINESS	SCHWINDEL	5:35	5:00	MILD	POSS
NER/CNS/B	ZK0.750	47	1870	EUPHORIA	SELBSTWAHRNEHMUNG DER GANGUNSICHERHEIT EINGESCHRAENKT	5:35	25:00	MILD	POSS
NER/CNS/B	ZK0.750	47	215	AMNESIA	GEDAECHTNISSTOERUNG	6:35	9:15	MILD	POSS
NER/CNS/B	ZK0.750	47	1470	DIZZINESS	PB VERHÄLT SICH WIE "ANGETRUNKEN" !	11:35	4:15	MILD	POSS
NER/CNS/B	ZK0.750	47	1870	EUPHORIA	LEICHTE EUPHORIE	11:35	4:15	MILD	POSS
NER/CNS/B	ZK0.750	47	4597	SEDATION	PB IST SCHWER ERWECKBAR	15:50	0:01	MODE	POSS
NER/CNS/B	ZK0.750	49	1105	CNS DEPRESS	VERLANGSAMTE UNSICHERE REAKTIONEN BEIM UMSTECKTEST	3:03	5:10	MILD	POSS
NER/CNS/B	ZK0.750	49	1870	EUPHORIA	SELBSTWAHRNEHMUNG DER GANGUNSICHERHEIT NICHT VORHANDEN	5:38	3:15	MODE	POSS
NER/CNS/B	ZK0.750	49	4597	SEDATION	MÜDIGKEIT, BEIM EEG 8 XIGES ANSPRECHEN BEI "AUGEN OFFEN"	8:31	0:02	MODE	POSS
NER/CNS/B	ZK0.750	49	215	AMNESIA	GEDÄCHTNISSTÖRUNG	10:13	2:25	MILD	POSS
NER/CNS/B	ZK0.750	49	215	AMNESIA	GEDÄCHTNISSTÖRUNG	15:55	0:02	MILD	POSS
NER/CNS/B	ZK0.750	50	4597	SEDATION	MÜDIGKEIT	2:35	0:18	MILD	POSS
NER/CNS/B	ZK0.750	50	215	AMNESIA	GEDÄCHTNISLÜCKE	23:30	0:02	MILD	POSS
NER/CNS/B	ZK1.500	52	1870	EUPHORIA	EUPHORIE	2:37	21:13	MILD	POSS
NER/CNS/B	ZK1.500	52	4685	SPEECH DIS	VEREINZELT LALLENDE LAUTBILDUNG BEIM SPRECHEN	3:07	20:43	MILD	POSS
NER/CNS/B	ZK1.500	52	4597	SEDATION	MÜDIGKEIT	4:55	1:20	MILD	POSS
NER/CNS/B	ZK1.500	52	4597	SEDATION	MÜDIGKEIT	6:15	17:35	MODE	POSS
NER/CNS/B	ZK1.500	52	1470	DIZZINESS	SCHWINDEL	8:25	38:35	MILD	POSS
NER/CNS/B	ZK1.500	52	1470	DIZZINESS	RAUSCHZUSTAND	23:50	3:15	MILD	POSS
NER/CNS/B	ZK1.500	52	4685	SPEECH DIS	VERWASCHENE SPRACHE	23:50	3:15	MILD	POSS
NER/CNS/B	ZK1.500	52	4105	PERSON DIS	HÄUFIGE WIEDERHOLUNG SEINER ERZÄHLUNGEN	28:30	7:30	MILD	POSS
NER/CNS/B	ZK1.500	52	215	AMNESIA	PB KONNTE SICH NICHT AN DIE MORGENTLICHE BETREUUNG NW ERINNERN	31:45	0:01	MILD	POSS
NER/CNS/B	ZK1.500	52	4597	SEDATION	MATTIGKEIT	48:36	10:54	MILD	POSS
NER/CNS/B	ZK1.500	53	1470	DIZZINESS	PB. FÜHLT SICH WIE BETRUNKEN	4:13	3:57	MILD	POSS
NER/CNS/B	ZK1.500	53	1470	DIZZINESS	RAUSCHZUSTAND	4:13	17:17	MODE	POSS
NER/CNS/B	ZK1.500	53	1105	CNS DEPRESS	VERLANGSAMTES REAKTIONSVERMÖGEN	5:27	25:33	MODE	POSS
NER/CNS/B	ZK1.500	53	4597	SEDATION	MÜDIGKEIT	6:07	2:03	MILD	POSS
NER/CNS/B	ZK1.500	53	4685	SPEECH DIS	VERWASCHENE SPRACHE	6:25	1:45	MODE	POSS
NER/CNS/B	ZK1.500	53	1177	CONCENTRATION ABNORM	KONZENTRATIONSSTOERUNG	10:05	0:13	MODE	POSS
NER/CNS/B	ZK1.500	53	1470	DIZZINESS	RAUSCHZUSTAND	21:30	9:30	MILD	POSS
NER/CNS/B	ZK1.500	53	4685	SPEECH DIS	SPRACHE VERLANGSAMT	21:30	9:30	MILD	POSS
NER/CNS/B	ZK1.500	55	1470	DIZZINESS	PB WIRKT WIE ALKOHOLISIERT	3:59	44:00	MILD	POSS
NER/CNS/B	ZK1.500	55	1870	EUPHORIA	PB WIRKT WIE ALKOHOLISIERT	3:59	0:00	MILD	POSS

ATA-LISTING OF ADVERSE EVENTS (AE TC2083)
ER BODY-SYSTEM, TREATMENT, DOSE, SUBJECT, AE CODE (NO), AE CODE (TERM),
VENT (GERMAN), STARTTIME, DURATION, INTENSITY AND DRUG RELATION

BODY-SYSTEM	GROUP	SUB	AE-CODE NO	TERM	EVENT	START TIME	DURA-TION	INTEN-SITY	DRUG RELAT
NER/CNS/B	ZK1.500	55	4685	SPEECH DIS	VERWASCHENE SPRACHE	5:44	18:30	MILD	POSS
NER/CNS/B	ZK1.500	55	4597	SEDATION	MÜDIGKEIT	6:30	26:59	MODE	POSS
NER/CNS/B	ZK1.500	55	215	AMNESIA	GEDÄCHTNISSTÖRUNG: IST ER IM KRANKENHAUS O. SCHERING?	6:44	41:15	MODE	POSS
NER/CNS/B	ZK1.500	55	1180	CONFUS	ZEITLICHE UND RÄUMLICHE DESORIENTIERUNG	6:44	30:45	MILD	POSS
NER/CNS/B	ZK1.500	55	1180	CONFUS	ZEITLICHE DESORIENTIERUNG	22:54	10:35	MILD	POSS
NER/CNS/B	ZK1.500	56	4685	SPEECH DIS	DER PB FÜHRT SELBSTGESPRÄCHE	-8:30	56:35	MILD	POSS
NER/CNS/B	ZK1.500	56	1470	DIZZINESS	PB. HAT DAS GEFÜHL, ALS WENN ER ALKOHOL GETRUNKEN HÄTTE.	4:30	1:30	MILD	POSS
NER/CNS/B	ZK1.500	56	4685	SPEECH DIS	PB. BEMERKT AN SICH SELBER EINE VERWASCHENE SPRACHE MIT AKZENT.	4:30	1:30	MILD	POSS
NER/CNS/B	ZK1.500	56	1720	EMOTION LABIL	TRAURIGKEIT	5:00	5:30	MILD	POSS
NER/CNS/B	ZK1.500	56	4597	SEDATION	MÜDIGKEIT	6:15	9:43	MILD	POSS
NER/CNS/B	ZK1.500	56	1470	DIZZINESS	"PB HAT DAS GEFÜHL BETRUNKEN ZU SEIN".	7:45	14:20	MODE	POSS
NER/CNS/B	ZK1.500	56	1177	CONCENTRATION ABNORM	KONZENTRATIONSSTÖRUNG	10:00	12:05	MODE	POSS
NER/CNS/B	ZK1.500	56	215	AMNESIA	GEDÄCHTNISSTÖRUNG, DER PB WEIß NICHT WO ER WOHNT UND WO SEIN AUTO	11:30	0:15	MILD	POSS
NER/CNS/B	ZK1.500	56	1105	CNS DEPRESS	VERLANGSAMTES REAKTIONSVERMÖGEN	15:58	32:07	MODE	POSS
NER/CNS/B	ZK1.500	56	4597	SEDATION	MÜDIGKEIT	15:58	0:32	SEVE	POSS
NER/CNS/B	ZK1.500	56	4597	SEDATION	MÜDIGKEIT	16:30	0:00	MILD	POSS
NER/CNS/B	ZK1.500	56	215	AMNESIA	VERMINDERTES ERINNERUNGSVERMÖGEN	22:05	10:25	SEVE	POSS
NER/CNS/B	ZK1.500	56	1215	COORDINAT ABNORM	UNSICHERHEIT BEIM STEHEN	26:00	22:05	MILD	POSS
NER/CNS/B	ZK1.500	56	5410	VERTIGO	DREHSCHWINDEL	26:00	8:30	MILD	POSS
NER/CNS/B	ZK1.500	56	1180	CONFUS	DER PB HAT KEIN ZEITGEFÜHL	31:30	16:35	MILD	POSS
NER/CNS/B	ZK1.500	56	4597	SEDATION	MÜDIGKEIT	40:30	7:35	MILD	POSS
NER/CNS/B	ZK1.500	77	1105	CNS DEPRESS	VERLANGSAMTES REAKTIONSVERMÖGEN	3:00	-3:30	MILD	POSS
NER/CNS/B	ZK1.500	77	1215	COORDINAT ABNORM	KOORDINATIONSSTÖRUNG	3:00	20:30	MODE	POSS
NER/CNS/B	ZK1.500	77	5410	VERTIGO	DREHSCHWINDEL	3:00	5:40	MODE	POSS
NER/CNS/B	ZK1.500	77	4685	SPEECH DIS	VERWASCHENE SPRACHE	7:10	-9:10	MILD	POSS
NER/CNS/B	ZK1.500	77	4597	SEDATION	MÜDIGKEIT	8:08	0:12	MODE	POSS
NER/CNS/B	ZK1.500	77	4597	SEDATION	MÜDIGKEIT	8:20	3:59	MILD	POSS
NER/CNS/B	ZK1.500	77	1470	DIZZINESS	PROBAND WIRKT WIE BETRUNKEN	9:00	14:30	MILD	POSS
NER/CNS/B	ZK1.500	77	215	AMNESIA	GEDÄCHTNISSTÖRUNG	9:50	-10:20	MILD	POSS
NER/CNS/B	ZK1.500	77	4105	PERSON DIS	SELBSGESPRÄCHE	10:15	2:45	MILD	POSS
NER/CNS/B	ZK1.500	77	4597	SEDATION	MUEDIGKEIT	12:20	11:10	MODE	POSS
NER/CNS/B	ZK1.500	77	4105	PERSON DIS	SELBSTGESPRÄCHE	27:00	0:30	MILD	POSS
NER/CNS/B	ZK1.500	78	1470	DIZZINESS	"DÜSELIGES GEFÜHL, WIE ALS WENN ICH ETWAS GETRUNKEN HÄTTE"	0:17	6:13	MILD	POSS
NER/CNS/B	ZK1.500	78	1105	CNS DEPRESS	"REAKTIONSVERMÖGEN LÄßT NACH, WERDE LANGSAMER"	0:37	23:53	MILD	POSS
NER/CNS/B	ZK1.500	78	4597	SEDATION	MÜDIGKEIT	1:00	7:30	MILD	POSS
NER/CNS/B	ZK1.500	78	1215	COORDINAT ABNORM	SEINE SCHRIFT VOM SZT WIRD QUALITATIV SCHLECHTER	4:03	20:27	MILD	POSS
NER/CNS/B	ZK1.500	78	1470	DIZZINESS	PROB. WIRKT ALKOHOLISIERT	4:30	4:00	MILD	POSS
NER/CNS/B	ZK1.500	78	4685	SPEECH DIS	VERWASCHENE SPRACHE	4:30	20:00	MILD	POSS
NER/CNS/B	ZK1.500	78	215	AMNESIA	RETROGRADE AMNESIE	6:30	17:00	MILD	POSS
NER/CNS/B	ZK1.500	78	1470	DIZZINESS	PROBAND WIRKT BETRUNKEN	8:30	12:05	MODE	POSS
NER/CNS/B	ZK1.500	78	4597	SEDATION	MÜDIGKEIT	8:30	4:10	MODE	POSS
NER/CNS/B	ZK1.500	78	1105	CNS DEPRESS	SZT NICHT DURCHFÜHRBAR	10:00	0:15	MODE	POSS
NER/CNS/B	ZK1.500	78	215	AMNESIA	ANTEROGRADE AMNESIE	12:00	11:30	MILD	POSS
NER/CNS/B	ZK1.500	78	4597	SEDATION	MÜDIGKEIT	12:40	7:55	SEVE	POSS
NER/CNS/B	ZK1.500	78	4597	SEDATION	MÜDIGKEIT	20:35	5:55	MILD	POSS
NER/CNS/B	ZK1.500	78	4685	SPEECH DIS	SELBSTGESPRÄCHE	26:30	9:30	MILD	POSS
NER/GEN	PLACEBO	22	2285	HEADACHE	KOPFDRUCK	2:45	4:30	MILD	UNLI
NER/GEN	ZK0.015	2	2285	HEADACHE	KOPFSCHMERZEN	-15:40	1:00	MILD	NO
NER/GEN	ZK0.015	6	2285	HEADACHE	KOPFSCHMERZEN	2:15	7:10	MILD	UNLI
NER/GEN	ZK0.015	6	2285	HEADACHE	KOPFSCHMERZEN	27:40	5:15	MILD	NO
NER/GEN	ZK0.030	15	2285	HEADACHE	KOPFSCHMERZ	1:58	0:15	MILD	POSS
NER/GEN	ZK0.075	20	2285	HEADACHE	KOPFSCHMERZEN LINKSSEITIG	26:20	84:00	MILD	NO
NER/GEN	ZK0.750	42	2060	GAIT ABNORM	GANGUNSICHERHEIT	4:15	8:45	MILD	POSS
NER/GEN	ZK0.750	42	2060	GAIT ABNORM	GANGUNSICHERHEIT	25:00	2:30	MILD	POSS
NER/GEN	ZK0.750	44	2060	GAIT ABNORM	GANGUNSICHERHEIT	7:10	2:50	MILD	POSS
NER/GEN	ZK0.750	47	2060	GAIT ABNORM	GANGUNSICHERHEIT	5:35	5:00	MILD	POSS
NER/GEN	ZK0.750	47	2060	GAIT ABNORM	GANGUNSICHERHEIT	11:35	24:00	MILD	POSS
NER/GEN	ZK0.750	49	2060	GAIT ABNORM	GANGUNSICHERHEIT	5:11	3:42	MODE	POSS
NER/GEN	ZK1.500	52	2285	HEADACHE	KOPFSCHMERZEN NACH DEM AUFSTEHEN AUS DEM ÜBERNACHTUNGSBETT	-2:45	8:15	MILD	NO
NER/GEN	ZK1.500	52	2060	GAIT ABNORM	SCHWANKEN BEIM BALANCETEST	4:25	0:05	MILD	POSS
NER/GEN	ZK1.500	52	2285	HEADACHE	KOPFSCHMERZEN	8:25	15:25	MILD	POSS
NER/GEN	ZK1.500	52	2060	GAIT ABNORM	GANGUNSICHERHEIT	11:30	15:35	MODE	POSS
NER/GEN	ZK1.500	52	2060	GAIT ABNORM	GANGUNSICHERHEIT	27:05	20:55	MILD	POSS
NER/GEN	ZK1.500	52	2060	GAIT ABNORM	PB BERICHTET, DAß ER NOCH GGL. LEICHTE GANGSTÖRUNGEN HAT	48:36	10:54	MILD	POSS
NER/GEN	ZK1.500	53	2060	GAIT ABNORM	GANGUNSICHERHEIT: ASSISTENZ NÖTIG	4:13	17:17	MODE	POSS

DATA-LISTING OF ADVERSE EVENTS (AE TC2083)
PER BODY-SYSTEM, TREATMENT, DOSE, SUBJECT, AE CODE (NO), AE CODE (TERM),
EVENT (GERMAN), STARTTIME, DURATION, INTENSITY AND DRUG RELATION

BODY-SYSTEM	GROUP	SUB	AE-CODE NO	TERM	EVENT	START TIME	DURA-TION	INTEN-SITY	DRUG RELAT
NER/GEN	ZK1.500	53	2060	GAIT ABNORM	GANGUNSICHERHEIT	21:30	12:30	MILD	POSS
NER/GEN	ZK1.500	53	2060	GAIT ABNORM	UNSICHERHEIT BEIM GEHEN	47:50	4:10	MILD	POSS
NER/GEN	ZK1.500	55	2060	GAIT ABNORM	GANGUNSICHERHEIT	4:19	22:10	MODE	POSS
NER/GEN	ZK1.500	55	2060	GAIT ABNORM	GANGUNSICHERHEIT	26:29	21:30	MILD	POSS
NER/GEN	ZK1.500	55	2285	HEADACHE	KOPFSCHMERZEN	34:29	13:00	MILD	POSS
NER/GEN	ZK1.500	55	2285	HEADACHE	KOPFSCHMERZEN	95:29	72:00	MODE	POSS
NER/GEN	ZK1.500	55	2285	HEADACHE	KOPFSCHMERZEN	167:29	48:00	MILD	POSS
NER/GEN	ZK1.500	56	2285	HEADACHE	KOPFSCHMERZ	11:45	10:20	MODE	POSS
NER/GEN	ZK1.500	56	2060	GAIT ABNORM	UNSICHERHEIT BEIM STEHEN	26:00	0:00	MILD	POSS
NER/GEN	ZK1.500	56	2060	GAIT ABNORM	GANGSTÖRUNGEN	29:30	19:00	MILD	POSS
NER/GEN	ZK1.500	77	2060	GAIT ABNORM	GANGUNSICHERHEIT	3:00	-3:30	MODE	POSS
NER/GEN	ZK1.500	77	2060	GAIT ABNORM	GANGUNSICHERHEIT	23:30	23:00	MILD	POSS
NER/GEN	ZK1.500	78	2060	GAIT ABNORM	GANGUNSICHERHEIT	11:30	15:00	MODE	POSS
NER/GEN	ZK1.500	78	2060	GAIT ABNORM	GANGUNSICHERHEIT	26:30	20:00	MILD	POSS
NER/PNS	PLACEBO	5	4050	PARESTHESIA	TAUBHEIT AN DER STIRN WEGEN ELEKTRODEN	2:56	1:30	MODE	NO
NER/PNS	PLACEBO	5	4050	PARESTHESIA	TAUBHEIT AN DER STIRN	6:57	6:33	MILD	NO
NER/PNS	ZK0.150	29	4050	PARESTHESIA	KRIBBELN AN HÄNDEN UND FÜßEN	0:40	0:04	MILD	POSS
RES/GEN	ZK1.500	77	4490	RESPIRAT DIS	ATEMBESCHWERDEN	6:30	1:05	MILD	POSS
RES/NOSE	PLACEBO	32	4530	RHINITIS	SCHNUPFEN	5:30	2:00	MILD	NO
RES/NOSE	ZK0.750	49	3728	NOSE DRY	VERSTOPFTE NASE	2:09	0:29	MILD	POSS
RES/SINS	ZK1.500	55	4655	SINUSITIS	DRUCKSCHMERZ I.B. DER LI. NASENNEBENHÖHLE MIT DEZENTER RÖTUNG.	95:29	120:00	MILD	POSS
SKIN/DERM/ERY	PLACEBO	3	1835	ERYTHEMA MULT	HAUTRÖTUNG AM KOPF (ELEKTRODEN)	13:55	22:17	MODE	NO
SKIN/DERM/ERY	PLACEBO	3	1835	ERYTHEMA MULT	HAUTRÖTUNG AM KOPF(ELEKTRODEN)	36:12	84:43	MILD	NO
SS/EAR/GEN	ZK1.500	52	1560	EAR DIS	DUMPFES GEFÜHL IN DEN OHREN, DEDÄMPFTES HÖREN	8:25	15:25	MILD	POSS
SS/EYE/CON	ZK0.750	45	1185	CONJUNCTIVITIS	GERÖTETE AUGEN	3:30	3:30	MILD	POSS
SS/EYE/CON	ZK0.750	49	1185	CONJUNCTIVITIS	GERÖTETE AUGEN	2:09	0:29	MILD	POSS
SS/EYE/CON	ZK1.500	55	1185	CONJUNCTIVITIS	GERÖTETE AUGEN	4:08	20:06	MILD	POSS
SS/EYE/GEN	ZK0.015	7	3920	PAIN EYE	AUGENBRENNEN	12:26	0:09	MODE	NO
SS/EYE/GEN	ZK0.750	49	3077	LACRIMATION DIS	TRÄNENDE AUGEN	2:09	0:29	MILD	POSS
SS/EYE/GEN	ZK1.500	56	3077	LACRIMATION DIS	PB HAT DAS GEFÜHL DIE AUGEN TRÄNEN	6:30	1:30	MILD	POSS
SS/EYE/VIS	ZK0.030	15	5420	VISION ABNORM	DER PB SAH NEBELWOLKEN BEI DER EEG-ABLEITUNG MIT OFFENEN AUGEN	0:52	0:02	MILD	POSS
SS/EYE/VIS	ZK0.030	15	5420	VISION ABNORM	PB15 HATTE SEHSTÖHRUNGEN	3:22	0:02	MILD	POSS
SS/EYE/VIS	ZK0.150	29	4140	PHOTOPHOBIA	UNANGENEHME BLENDUNG DURCH HELLE OBJEKTE	3:10	2:20	MILD	POSS
SS/EYE/VIS	ZK0.300	33	4140	PHOTOPHOBIA	LICHTEMPFINDLICHKEIT	5:32	3:10	SEVE	PROB
SS/EYE/VIS	ZK0.300	35	4140	PHOTOPHOBIA	LICHTEMPFINDLICHKEIT DER AUGEN, WIE FLACKERN	3:10	0:47	MILD	POSS
SS/EYE/VIS	ZK0.300	35	4140	PHOTOPHOBIA	LICHTEMPFINDLICHKEIT	4:02	0:33	MILD	POSS
SS/EYE/VIS	ZK0.300	35	4140	PHOTOPHOBIA	LICHTEMPFINDLICHKEIT	5:30	0:30	SEVE	POSS
SS/EYE/VIS	ZK0.300	35	4140	PHOTOPHOBIA	LICHTEMPFINDLICHKEIT	6:00	1:40	MILD	POSS
SS/EYE/VIS	ZK0.300	38	205	VISION BLURRED	VERSCHWOMMENES SEHEN	2:05	5:30	MILD	POSS
SS/EYE/VIS	ZK0.300	39	5420	VISION ABNORM	LEICHTER NEBEL BEIM SEHEN	1:11	4:07	MILD	POSS

DATA-LISTING OF ADVERSE EVENTS (AE TC2083)
PER BODY-SYSTEM, TREATMENT, DOSE, SUBJECT, AE CODE (NO), AE CODE (TERM),
EVENT (GERMAN), STARTTIME, DURATION, INTENSITY AND DRUG RELATION

BODY-SYSTEM	GROUP	SUB	AE-CODE NO	TERM	EVENT	START TIME	DURA-TION	INTEN-SITY	DRUG RELAT
SS/EYE/VIS	ZK0.300	39	205	VISION BLURRED	VERSCHWOMMENES SEHEN	3:04	4:37	MILD	POSS
SS/EYE/VIS	ZK0.300	39	1450	DIPLOPIA	DOPPELTSEHEN	3:26	2:00	MILD	POSS
SS/EYE/VIS	ZK0.300	39	5420	VISION ABNORM	WOLKIGE BEWEGUNGEN DER BETTDECKE	3:26	4:15	MILD	POSS
SS/EYE/VIS	ZK0.300	40	205	VISION BLURRED	VERSCHWOMMENES SEHEN	1:00	4:25	MILD	POSS
SS/EYE/VIS	ZK0.300	40	1450	DIPLOPIA	DOPPELTSEHEN	2:10	3:15	MILD	POSS
SS/EYE/VIS	ZK0.300	40	5420	VISION ABNORM	SEHEN VON KREISENDEN BUNTEN PUNKTEN	2:25	0:05	MILD	POSS
SS/EYE/VIS	ZK0.300	40	4140	PHOTOPHOBIA	LICHTEMPFINDLICHKEIT DER AUGEN	2:30	5:15	MILD	POSS
SS/EYE/VIS	ZK0.750	42	4140	PHOTOPHOBIA	EXTREM HELLES LICHT IM KÄFIG, KITTEL REFLEKTIEREN STARK	1:00	1:50	MODE	POSS
SS/EYE/VIS	ZK0.750	42	5420	VISION ABNORM	WELLENFÖRMIGE BEWEGUNGEN AN DER TÜR	1:00	9:15	MILD	POSS
SS/EYE/VIS	ZK0.750	42	1060	CHROMATOPSIA	LILA FARBEFFEKTE AUF DEM PAPIER; SCHWARZE UNTERL. ERSCHEINT BLAU	1:20	1:30	MILD	POSS
SS/EYE/VIS	ZK0.750	42	5420	VISION ABNORM	BUCHSTABEN ZEIGEN EINEN SCHATTEN	1:20	4:47	MILD	POSS
SS/EYE/VIS	ZK0.750	42	4140	PHOTOPHOBIA	HELLES LICHT IM KÄFIG, KITTEL REFLEKTIEREN	2:51	0:54	MILD	POSS
SS/EYE/VIS	ZK0.750	42	5420	VISION ABNORM	DAMPFWOLKEN VOR DEN AUGEN, WIE IN DER WASCHKÜCHE, NEBELSCHWADEN	6:07	6:53	MILD	POSS
SS/EYE/VIS	ZK0.750	42	1060	CHROMATOPSIA	VERÄNDERTE FARBWAHRNEHMUNG VON KLEIDUNGSSTÜCKEN	8:05	0:15	MODE	POSS
SS/EYE/VIS	ZK0.750	43	205	VISION BLURRED	LEICHTE TRÜBUNG BEIM SCHREIBEN, WIE IM NEBEL	1:03	6:42	MILD	POSS
SS/EYE/VIS	ZK0.750	44	5420	VISION ABNORM	FLIMMERN VOR DEN AUGEN, DUNKEL UND HELL	1:01	7:29	MILD	POSS
SS/EYE/VIS	ZK0.750	44	5420	VISION ABNORM	FENSTERKONTUREN BEWEGEN SICH	1:36	6:24	MILD	POSS
SS/EYE/VIS	ZK0.750	44	205	VISION BLURRED	VERSCHWOMMENES SEHEN	3:00	5:00	MILD	POSS
SS/EYE/VIS	ZK0.750	44	5420	VISION ABNORM	NEBEL VOR DEN AUGEN BEI BLICK I.D. FERNE	5:00	3:00	MILD	POSS
SS/EYE/VIS	ZK0.750	44	5420	VISION ABNORM	SEHEN VON SCHATTEN, DIE SICH BEWEGEN	5:30	3:30	MILD	POSS
SS/EYE/VIS	ZK0.750	45	5420	VISION ABNORM	TRÜBUNG OHNE SEHSCHÄRFEVERLUST	0:35	2:04	MILD	POSS
SS/EYE/VIS	ZK0.750	45	1450	DIPLOPIA	DOPPELTSEHEN VON BUCHSTABEN	1:10	6:50	MILD	POSS
SS/EYE/VIS	ZK0.750	45	5420	VISION ABNORM	BRAUNE FARBRÄNDER AN DEN BUCHSTABEN; KEINE FARBVERÄNDERUNGEN	2:35	5:25	MILD	POSS
SS/EYE/VIS	ZK0.750	45	5420	VISION ABNORM	TRÜBUNG	2:40	5:20	MODE	POSS
SS/EYE/VIS	ZK0.750	45	4140	PHOTOPHOBIA	LICHTEMPFINDLICHKEIT DER AUGEN: HELLERE FARBEN	2:50	5:10	MILD	POSS
SS/EYE/VIS	ZK0.750	45	1060	CHROMATOPSIA	VERÄNDERTE FARBWAHRNEHMUNG: LILA WIE BLAU, ROT WIE BRAUN	3:05	2:10	MILD	POSS
SS/EYE/VIS	ZK0.750	45	5420	VISION ABNORM	SCHATTEN AUF DEM HANDRÜCKEN UND BETTDECKE	3:45	2:15	MILD	POSS
SS/EYE/VIS	ZK0.750	46	5420	VISION ABNORM	ROTBRAUNE SCHATTEN, DIE SICH BEWEGEN UND VERSCHWINDEN	1:45	7:15	MILD	POSS
SS/EYE/VIS	ZK0.750	46	5420	VISION ABNORM	NACH RUHEPHASEN: GEFÜHL, DAß D. FENSTER AN DER DECKE SEI.	2:00	2:15	MILD	POSS
SS/EYE/VIS	ZK0.750	47	4140	PHOTOPHOBIA	ALLES IST HELLER; WIE VON DER SONNE ÜBERSTRAHLT	0:55	0:05	MILD	POSS
SS/EYE/VIS	ZK0.750	47	4140	PHOTOPHOBIA	LICHTEMPFINDLICHKEIT	1:00	2:54	MODE	POSS
SS/EYE/VIS	ZK0.750	47	5420	VISION ABNORM	"DIE BILDTEILE BEWEGEN SICH"	1:00	0:49	MILD	POSS
SS/EYE/VIS	ZK0.750	47	205	VISION BLURRED	VERSCHWOMMENES SEHEN	1:00	3:35	MILD	POSS
SS/EYE/VIS	ZK0.750	47	5420	VISION ABNORM	BRAUN-ROTE PUNKTE MIT WECHSELNDER GRÖßE U.BEWEGUNG AUF D.HAUT	1:25	9:10	MILD	POSS
SS/EYE/VIS	ZK0.750	47	5420	VISION ABNORM	GEGENSTÄNDE BEWEGEN SICH VOR UND ZURÜCK UND WERDEN DUNKLER.	1:50	4:45	MILD	POSS
SS/EYE/VIS	ZK0.750	47	5420	VISION ABNORM	ALLE GEGENSTÄNDE BEWEGEN SICH CA. 1 MAL PRO SEC	1:50	4:45	MODE	POSS
SS/EYE/VIS	ZK0.750	47	205	VISION BLURRED	SEHSCHÄRFE VERMINDERT BEIM LESEN (SDT5); DADURCH MOTOR. VERLANGS.	2:03	4:32	MODE	POSS
SS/EYE/VIS	ZK0.750	47	5420	VISION ABNORM	LÖCHER D.PEG6 MIT SICH BEWEGENDEN BRAUNEN SCHATTEN UMGEBEN;	3:05	4:05	MODE	POSS
SS/EYE/VIS	ZK0.750	47	4140	PHOTOPHOBIA	LICHTEMPFINDLICHKEIT GERINGER, FLÄCHEN WIRKEN DUNKLER	3:55	3:15	MILD	POSS
SS/EYE/VIS	ZK0.750	47	4140	PHOTOPHOBIA	BLICK AUS D. FENSTER: "LICHTWIRKUNG, WIE NACH SCHNEEFALL"	5:35	2:15	MILD	POSS
SS/EYE/VIS	ZK0.750	49	205	VISION BLURRED	VERSCHWOMMENES SEHEN IN DER FERNE	0:23	5:45	MILD	POSS
SS/EYE/VIS	ZK0.750	49	4140	PHOTOPHOBIA	HELLES NACHBILD WIE SCHEINWERFERLICHT FÜR 10 SEK	0:53	0:01	MILD	POSS
SS/EYE/VIS	ZK0.750	49	5420	VISION ABNORM	DER RAUM ERSCHEINT DUNKLER.	0:55	0:00	MILD	POSS
SS/EYE/VIS	ZK0.750	49	5420	VISION ABNORM	DER RAUM ERSCHEINT DEUTLICH DUNKLER	2:10	6:43	MODE	POSS
SS/EYE/VIS	ZK0.750	49	5420	VISION ABNORM	OBJEKTE DRAUßEN DUNKEL UND MILCHIG; WARUM AUTOS OHNE LICHT?	5:38	1:24	MODE	POSS
SS/EYE/VIS	ZK0.750	49	45	ACCOMMODATION ABNORM	AKKOMMODATIONSTÖRUNGEN: SCHARFSEHEN DER FERNEN OBJEKTE NACH 2 SEK	6:08	2:35	MILD	POSS
SS/EYE/VIS	ZK0.750	49	5420	VISION ABNORM	ALTERSFLECKEN AUF DER HAND WIRKEN DUNKLER	6:08	2:45	MILD	POSS
SS/EYE/VIS	ZK0.750	50	5420	VISION ABNORM	PULSIERENDE VERÄNDERUNGEN DES WANDBILDES	1:02	8:06	MILD	POSS
SS/EYE/VIS	ZK0.750	50	205	VISION BLURRED	VERSCHWOMMENES SEHEN VON	1:25	7:43	MILD	POSS

DATA-LISTING OF ADVERSE EVENTS (AE TC2083)
PER BODY-SYSTEM, TREATMENT, DOSE, SUBJECT, AE CODE (NO), AE CODE (TERM),
EVENT (GERMAN), STARTTIME, DURATION, INTENSITY AND DRUG RELATION

BODY-SYSTEM	GROUP	SUB	AE-CODE NO	/TERM	EVENT	START TIME	DURA- TION	INTEN- SITY	DRUG RELAT
SS/EYE/VIS	ZK0.750	50	205	VISION BLURRED	BUCHSTABEN VERSCHWOMMENES SEHEN ALLG. FERN U. NAH	5:00	4:08	MILD	POSS
SS/EYE/VIS	ZK0.750	50	5420	VISION ABNORM	SEHEN VON DUNKLEN FLECKEN BEI BEWEGUNGEN AUF GEGENSTÄNDEN,HÄNDEN	6:30	2:38	MILD	POSS
SS/EYE/VIS	ZK0.750	50	205	VISION BLURRED	SEHEN VON DUNKLEN FLECKEN BEI BEWEGUNGEN AUF GEGENSTÄNDEN,HÄNDEN	6:30	0:00	MILD	POSS
SS/EYE/VIS	ZK1.500	52	5420	VISION ABNORM	LEICHTES FLIMMERN VOR DEN AUGEN BEIM AUFSETZEN DER BRILLE	0:18	0:20	MILD	POSS
SS/EYE/VIS	ZK1.500	52	5420	VISION ABNORM	WEIßER FLECK V.D. AUGEN,B. SCHLIEßEN D.AUGEN DUNKEL M. WEIßEM RAND	0:27	0:35	MILD	POSS
SS/EYE/VIS	ZK1.500	52	5420	VISION ABNORM	MILCHIGES SEHEN,DER RAUM ERSCHEINT HELLER	0:38	3:17	MILD	POSS
SS/EYE/VIS	ZK1.500	52	1060	CHROMATOPSIA	FARBSEHEN BEEINTRÄCHTIGT, UNTERSCHEIDUNG VON FARBEN SCHWIERIG	0:45	3:35	MILD	POSS
SS/EYE/VIS	ZK1.500	52	5420	VISION ABNORM	SCHLECHTERES LESEN BEIM SYMBOLZAHLENTEST	1:08	3:02	MILD	POSS
SS/EYE/VIS	ZK1.500	52	5420	VISION ABNORM	PB KANN BEIM SEHTEST DEN ANGEZEIGTEN PUNKT NICHT FIXIEREN	2:37	6:42	MODE	POSS
SS/EYE/VIS	ZK1.500	52	5420	VISION ABNORM	RAUM ERSCHEINT DUNKLER	3:55	19:55	MILD	POSS
SS/EYE/VIS	ZK1.500	52	5420	VISION ABNORM	SCHLECHTES LESEN BEIM SYMBOLZAHLENTEST	4:10	0:05	MODE	POSS
SS/EYE/VIS	ZK1.500	52	5420	VISION ABNORM	BEIM AUGENTEST (BLICKRICHTUNGSTEST) WANDERN DIE AUGEN ZURÜCK	9:19	0:01	MILD	POSS
SS/EYE/VIS	ZK1.500	52	4140	PHOTOPHOBIA	TAGESLICHT WIRD ALS GLEIßEND EMPFUNDEN	25:30	4:00	MILD	POSS
SS/EYE/VIS	ZK1.500	53	4140	PHOTOPHOBIA	ALLES WIRKT ETWAS HELLER	0:13	0:41	MILD	POSS
SS/EYE/VIS	ZK1.500	53	5420	VISION ABNORM	MILCHIGE TRÜBUNG DER SICHT OHNE SEHSCHÄRFEVERLUST	0:13	2:37	MILD	POSS
SS/EYE/VIS	ZK1.500	53	1060	CHROMATOPSIA	FARBEN WIRKEN BLASSER	0:35	1:00	MILD	POSS
SS/EYE/VIS	ZK1.500	53	4140	PHOTOPHOBIA	KITTEL SIND HELL WIE IN DER SCHÖNSTEN REKLAME	0:55	3:54	MODE	POSS
SS/EYE/VIS	ZK1.500	53	1060	CHROMATOPSIA	HELLES BLAU WIRKT WIE PINK	1:40	6:15	MILD	POSS
SS/EYE/VIS	ZK1.500	53	5420	VISION ABNORM	WAND WIRKT DUNKLER ALS NORMAL; FOTO WIRKT KONTRASTREICHER	2:51	8:09	MILD	POSS
SS/EYE/VIS	ZK1.500	53	205	VISION BLURRED	BUCHSTABEN UND ZAHLEN SIND UNSCHARF	4:13	3:57	MILD	POSS
SS/EYE/VIS	ZK1.500	53	5420	VISION ABNORM	ALLES MIT GRAUSCHLEIER BEDECKT	4:50	3:20	MILD	POSS
SS/EYE/VIS	ZK1.500	53	5420	VISION ABNORM	WAND WIRKT DUNKLER ALS NORMAL	11:00	10:30	MILD	POSS
SS/EYE/VIS	ZK1.500	55	205	VISION BLURRED	MILCHIGE TRÜBUNG	0:31	0:21	MILD	POSS
SS/EYE/VIS	ZK1.500	55	205	VISION BLURRED	VERSCHWOMMENES SEHEN	0:53	4:50	MODE	POSS
SS/EYE/VIS	ZK1.500	55	4140	PHOTOPHOBIA	RAUM WIRKT DUNKLER	1:59	0:00	MILD	POSS
SS/EYE/VIS	ZK1.500	55	5420	VISION ABNORM	RAUM WIRKT DUNKLER	1:59	7:30	MILD	POSS
SS/EYE/VIS	ZK1.500	55	5420	VISION ABNORM	FLACKERNDES SEHEN	2:59	21:00	MILD	POSS
SS/EYE/VIS	ZK1.500	55	4140	PHOTOPHOBIA	RAUM WIRKT WIEDER HELLER, WENN AUGEN GÖFFNET WERDEN	3:19	6:10	MILD	POSS
SS/EYE/VIS	ZK1.500	55	205	VISION BLURRED	VERSCHWOMMENES SEHEN	5:44	18:15	MILD	POSS
SS/EYE/VIS	ZK1.500	56	5420	VISION ABNORM	"NEBEL SEHEN"	0:20	21:45	MILD	POSS
SS/EYE/VIS	ZK1.500	56	1060	CHROMATOPSIA	FARBEN SIND ZUM TEIL VERÄNDERT	1:00	29:30	MILD	POSS
SS/EYE/VIS	ZK1.500	56	5420	VISION ABNORM	OBJEKTE IN DER UMGEBUNG SEHEN HELLER AUS	1:00	7:50	MILD	POSS
SS/EYE/VIS	ZK1.500	56	5420	VISION ABNORM	BRAUNE FLECKE AUF OBJEKTEN	1:25	6:35	MILD	POSS
SS/EYE/VIS	ZK1.500	56	5420	VISION ABNORM	RAUM ERSCHEINT DUNKEL, MILCHKAFFEE SCHWARZ	8:50	13:15	MILD	POSS
SS/EYE/VIS	ZK1.500	56	5420	VISION ABNORM	BRAUNE FLECKEN AUF OBJEKTEN	10:35	11:30	MILD	POSS
SS/EYE/VIS	ZK1.500	56	4140	PHOTOPHOBIA	RAUMLICHT BLENDET	16:05	6:00	MODE	POSS
SS/EYE/VIS	ZK1.500	56	5420	VISION ABNORM	SEHEN VON HELLGRAUEN "WOLKEN"	22:05	3:55	MODE	POSS
SS/EYE/VIS	ZK1.500	56	5420	VISION ABNORM	"HELLGRAUE WOLKEN" VOR HINTERGRUND	26:00	2:30	MILD	POSS
SS/EYE/VIS	ZK1.500	77	5420	VISION ABNORM	PB. SIEHT STERNE BEI GESCHLOSENEN AUGEN,DUNKLER BEI OFFENEN AUGE	0:25	0:09	MILD	POSS
SS/EYE/VIS	ZK1.500	77	5420	VISION ABNORM	PB. SIEHT STERNE; SCHNELLER WECHSEL ZW. HELL/DUNKEL	0:35	5:04	MODE	POSS
SS/EYE/VIS	ZK1.500	77	205	VISION BLURRED	TRÜBUNG; FLECKEN UND SCHATTEN AUF DER HAND STÄRKER	0:55	4:44	MODE	POSS
SS/EYE/VIS	ZK1.500	77	5420	VISION ABNORM	PB. SIEHT BEWEGTE FLECKEN UND FLÄCHEN	3:00	2:39	MODE	POSS
SS/EYE/VIS	ZK1.500	77	205	VISION BLURRED	VERSCHWOMMENES SEHEN	5:40	3:00	MILD	POSS
SS/EYE/VIS	ZK1.500	78	5420	VISION ABNORM	FLIMMERN VOR DEN AUGEN	0:35	11:35	MILD	POSS
SS/EYE/VIS	ZK1.500	78	205	VISION BLURRED	BUCHSTABEN VERSCHWIMMEN UND TANZEN	1:00	19:35	MILD	POSS
SS/EYE/VIS	ZK1.500	78	4140	PHOTOPHOBIA	"WEIßER KITTEL LEUCHTET GRELL WIE BEIM WEIßEN RIESEN".	1:22	19:13	MILD	POSS
SS/EYE/VIS	ZK1.500	78	5420	VISION ABNORM	"DUNKLES UND HELLES SEHEN" IN INTERVALLEN WIE DER HERZSCHLAG	1:22	6:23	MILD	POSS
SS/EYE/VIS	ZK1.500	78	205	VISION BLURRED	"DUNKLES UND HELLES SEHEN" IN INTERVALLEN WIE DER HERZSCHLAG	1:22	0:00	MILD	POSS
SS/EYE/VIS	ZK1.500	78	5420	VISION ABNORM	SCHATTEN AUF DEN AUGEN UND MUND DER ASSISTENTIN	3:45	4:00	MILD	POSS
SS/EYE/VIS	ZK1.500	78	5420	VISION ABNORM	SCHRIFT LESEN IST WEITER SCHLECHTER GEWORDEN	4:05	1:10	MODE	POSS
SS/EYE/VIS	ZK1.500	78	5420	VISION ABNORM	SCHRIFT LESEN WIRD WIEDER BESSER	5:15	19:15	MILD	POSS

DATA-LISTING OF ADVERSE EVENTS (AE TC2083)
PER BODY-SYSTEM, TREATMENT, DOSE, SUBJECT, AE CODE (NO), AE CODE (TERM),
EVENT (GERMAN), STARTTIME, DURATION, INTENSITY AND DRUG RELATION

BODY-SYSTEM	GROUP	SUB	AE-CODE NO/TERM		EVENT	START TIME	DURA-TION	INTEN-SITY	DRUG RELAT
SS/TST	ZK1.500	55	4890	TASTE LOSS	GESCHMACKSVERLUST	21:09	1:10	MILD	POSS
UG/UT/B/F	PLACEBO	36	5275	URIN FREQUENCY	HARNDRANG	7:30	16:00	MODE	POSS
UG/UT/B/F	ZK0.750	46	5275	URIN FREQUENCY	HARNDRANG	-0:25	0:02	MODE	NO
UG/UT/B/F	ZK0.750	46	5275	URIN FREQUENCY	HARNDRANG	0:55	0:02	MODE	UNLI
UG/UT/B/F	ZK0.750	46	5275	URIN FREQUENCY	HARNDRANG	1:55	0:02	MODE	UNLI
UG/UT/B/F	ZK0.750	46	5275	URIN FREQUENCY	HARNDRANG	4:05	0:02	MODE	UNLI
UG/UT/B/F	ZK0.750	46	5275	URIN FREQUENCY	GEHAUFTES WASSERLASSEN	5:18	0:02	MODE	UNLI
UG/UT/B/F	ZK0.750	46	5275	URIN FREQUENCY	GEHAUFTES WASSERLASSEN	5:18	2:47	MILD	POSS
UG/UT/B/F	ZK0.750	49	5275	URIN FREQUENCY	VERMEHRTER HARNDRANG (4 MAL IN 4 H)	1:53	4:00	MILD	POSS
UG/UT/B/F	ZK1.500	56	5275	URIN FREQUENCY	VERMEHRTER HARNDRANG	4:00	7:00	MILD	POSS
UG/UT/B/F	ZK1.500	56	1555	DYSURIA	SUPRAPUBISCHER DRUCKSCHMERZ	23:00	11:30	MILD	POSS
UG/UT/B/F	ZK1.500	56	1555	DYSURIA	DER PB HAT SCHWIERIGKEITEN BEIM WASSER LASSEN.	23:30	24:35	MILD	POSS
UG/UT/B/F	ZK1.500	77	5275	URIN FREQUENCY	HARNDRANG	3:00	4:05	MODE	POSS
UG/UT/URN	ZK0.030	12	5265	URIN ABNORM	MIKROHÄMATORIE	8:07	27:57	MILD	POSS
UG/UT/URN	ZK0.030	12	5265	URIN ABNORM	MIKROALBUMINURIE	11:54	12:06	MILD	POSS
UG/UT/URN	ZK1.500	55	5265	URIN ABNORM	TRÜBER URIN	23:59	0:00	MODE	POSS

Duration and Intensity of Adverse Events by Subject
BODY SYSTEM = NER/CNS/B (NERVOUS SYSTEM/CENTRAL NERVOUS SYSTEM/BRAIN)
Worst Case per Hour

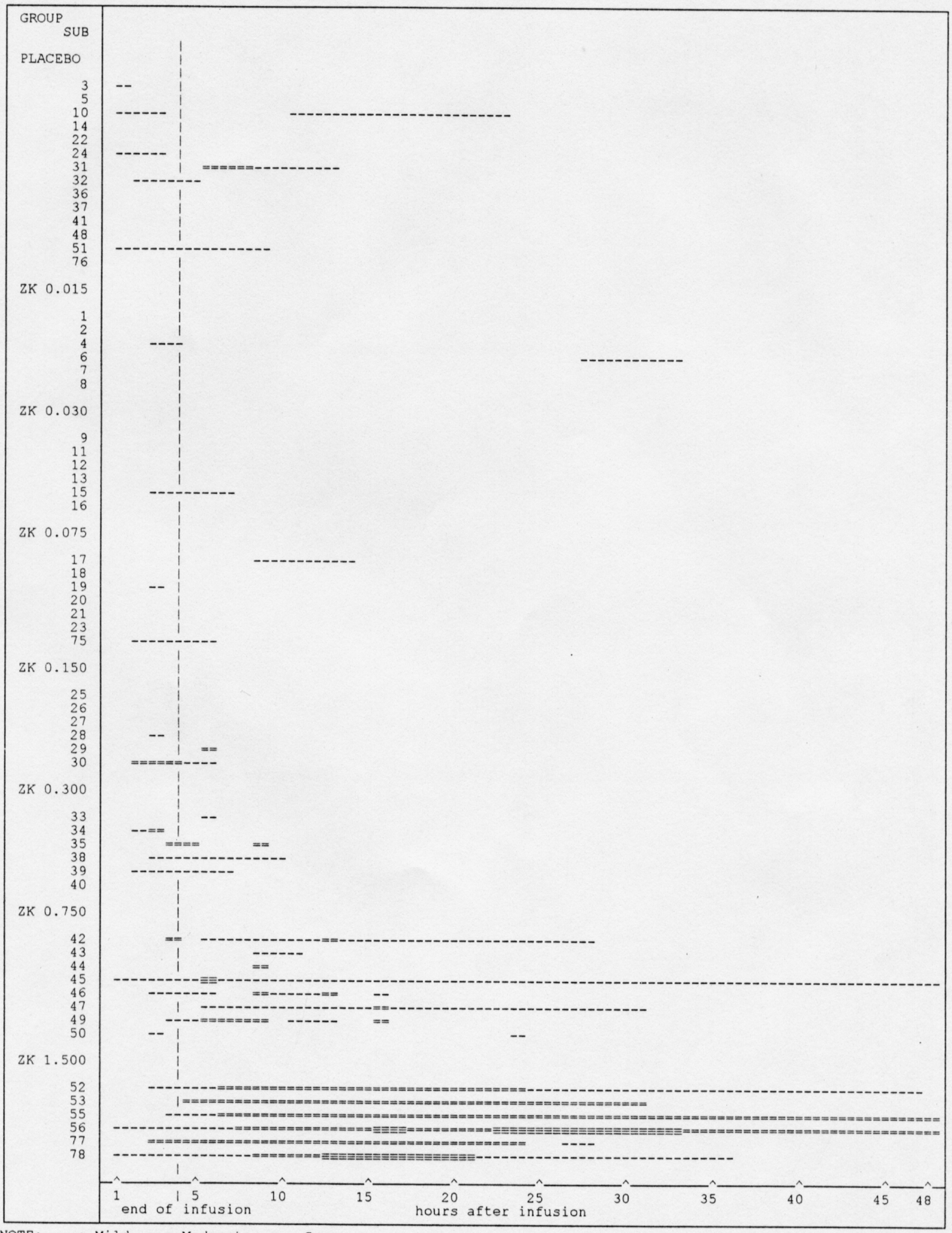

NOTE: - : Mild, = : Moderate, ≡ : Severe

Duration and Intensity of Adverse Events by Subject
BODY SYSTEM = SS/EYE/VIS (SPECIAL SENSES/EYE DISORDERS/VISION DISORDERS)
Worst Case per Hour

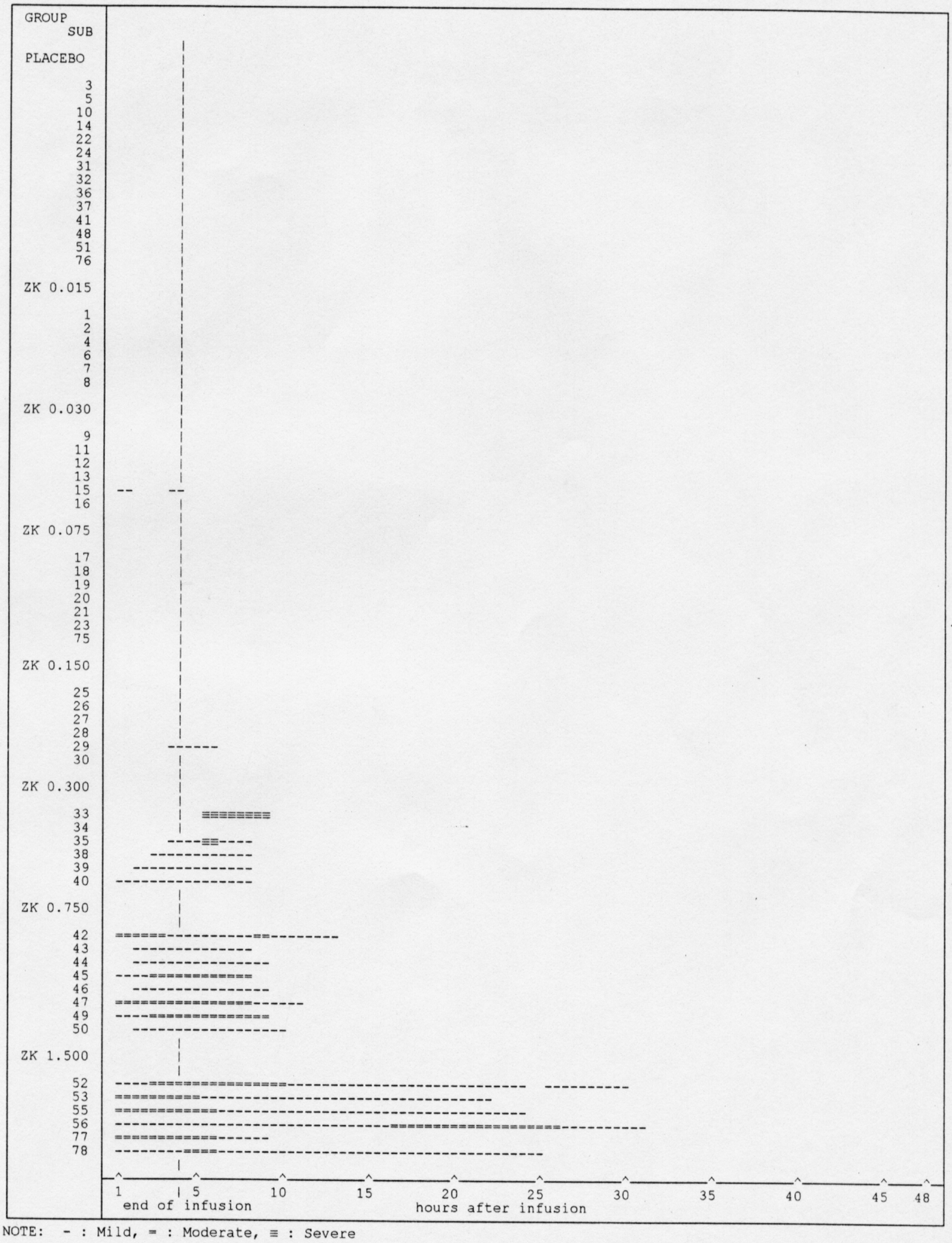

NOTE: - : Mild, = : Moderate, ≡ : Severe

Unterschriften für die Prüfplanendfassung:

Ophthalmologische Untersuchungen nach 4-stündiger Infusion mit ZK 200775 bei älteren gesunden Freiwilligen

Datum: 07 Feb. 97 **Autor: Y. Tauchmann / Dr. Th. Staks**

Funktion	Name	Verantwortlichkeit	Endfassung: Unterschrift, Datum
Leiter der klinischen Prüfung	Dr. Th. Staks	Planung, Durchführung, Auswertung	Th. Staks 10.2.97
Supervisor	Dr. W. Seifert	Beratung	W. Seifert 11.2.97
Biometrie	Dr. K. Fichte	biometr. Planung/ Auswertung	Fichte, 10.2.97
Vertriebsleiter	Dr. Th. Staks	Inverkehrbringen der Prüfpräparate	Th. Staks 10.2.97
Leiter 'Klinische Pharmakologie'	Dr. W. Seifert	Bereitstellung der Logistik	W. Seifert 11.2.97
Klinischer Entwicklungsleiter	Dr. C. Stürzebecher	Sponsor	[illegible] 13.2.97

Unterschriften für die Prüfplanendfassung:

Ophthalmologische Untersuchungen nach 4-stündiger Infusion mit ZK 200775 bei älteren gesunden Freiwilligen

Datum: 27.02.97 **Autor: Y. Tauchmann / Dr. Th. Staks**

Funktion	Name	Verantwortlichkeit	Endfassung: Unterschrift, Datum
Externer Prüfarzt	Dr. K. Rüther	Durchführung der ophthalmologischen Untersuchungen	25.3.97 [illegible]
