# Supplementary figures and images for: Effects of the AMPA Antagonist ZK 200775 on Visual Function: A Randomized Controlled Trial
Source: PLoS One. 2010 Aug 12;5(8):e12111. doi: 10.1371/journal.pone.0012111 (PMC2920815; doi:10.1371/journal.pone.0012111)

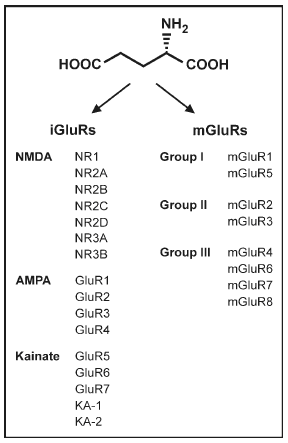

Supplement: Figure S1 — Glutamate receptor subtypes. (0.02 MB TIF) [file pone.0012111.s003.tif]
