# Supplementary material for: Effects of the AMPA Antagonist ZK 200775 on Visual Function: A Randomized Controlled Trial
Source: PLoS One. 2010 Aug 12;5(8):e12111. doi: 10.1371/journal.pone.0012111 (PMC2920815; doi:10.1371/journal.pone.0012111)
Supplement: Table S1 — Ionotropic glutamate receptor expression in retinal neurons and retinal layers. Immunocytochemistry, in situ hybridization and polymerase chain reaction. Based on Webvision, completed by the authors (http://webvision.med.utah.edu, author: Vikki P. Connaughton). Some papers could not be considered in this survey. (0.15 MB DOC) [file pone.0012111.s004.doc]

| **Retinal cell type or layer** | **Non-NMDA receptor subunits** | **NMDA receptor subunits** | **Species** | **Reference** |
| --- | --- | --- | --- | --- |
| Photoreceptors | GluR6/7 (single cone outer segments) |  | Goldfish | Peng et al.(1) |
|  | GluR6, KA1, (KA2) (rods) |  | Mouse | Jakobs et al.(2) |
| OPL | GluR2, GluR2/3, GluR6/7 |  | Rat | Peng et al. (1) |
|  | GluR2, 3 |  | Goldfish | Vandenbranden et al.(3) |
|  |  | NR2A (punctate) | Cat | Harveit et al.(4) |
|  | GluR2, GluR2/3 (photoreceptors) |  | Goldfish | Peng et al. (1) |
| Bipolar cells | GluR2 (Mb cells) |  | Goldfish | Peng et al. (1) |
|  | GluR2, GluR2/3 |  | Rat | Peng et al. (1) |
|  |  | NR2D (RBC) | Rat | Wenzel et al.(5) |
|  | GluR2 and/or GluR4 | NR1 (RBC) | Rat | Hughes(6) |
|  | GluR2 (RBC) |  | Rat | Hughes et al.(7) |
|  | GluR1 (flat midget bipolar cells) |  | Marmoset | Puller et al.(8) |
|  | GluR1, (2), 5, KA2 |  | Mouse | Jakobs et al.(2) |
|  | GluR4, 6 (RBC) |  | Macaque | Hanna et al.(9) |
|  | GluR2 (ON-BC) |  | Rat | Kamphuis et al.(10,11) |
|  | GluR4 (dendrites) |  | Goldfish | Vandenbranden et al.(3) |
| Horizontal cells | GluR6/7 |  | Goldfish | Peng et al. (1) |
|  | GluR2 |  | Goldfish | Klooster et al.(12) |
|  | GluR5-7 |  | Goldfish | Vandenbranden et al.(3) |
| INL | GluR2/3, GluR6/7 |  | Rat | Peng et al. (1) |
|  |  | NR2A (inner) | Rat | Hartveit et al. (4) |
|  | GluR1, 2, 5 > GluR4 (outer third), GluR1, 2, 5 (middle third), GluR1-5 (inner third) |  | Rat | Hughes et al.(7) |
|  | GluR1-7 |  | Rat, cat | Hamassaki-Britto et al.(13) |
|  | KA2 (homogeneous), GluR6 (inner), GluR7 (inner two-thirds) | NR1 (homogeneous), NR2A-B (inner third, patchy), NR2C (inner two-thirds) | Rat | Brandstatter et al.(14) |
|  | GluR1-4 (inner border), GluR3 (outer border) |  | Goldfish | Vandenbranden et. al.(3) |
| IPL | GluR1, GluR2/3, GluR6/7 |  | Rat | Peng et al. (1) |
|  |  | NR2A | Rat, cat, rabbit, monkey | Harveit et al. (4) |
| Amacrine cells | GluR6 | NR2A-C | Rat | Brandstatter et al. (14) |
|  | GluR1, GluR2/3 |  | Rat | Peng et al. (1) |
|  | GluR1, 2, 6, KA2 |  | Mouse | Jakobs et al.(2) |
| Ganglion cells | GluR1 |  | Rat | Peng et al. (1) |
|  | Glu R4, KA1, 2, δ1, 2 | NR1, 2A-D, 3A | Mouse | Jakobs et al.(2) |
|  | GluR1, 3, 4 |  | Goldfish | Vandenbranden et al.(3) |
| GCL | GluR2/3, GluR6/7 |  | Rat | Peng et al. (1) |
|  | GluR1-5 |  | Rat | Hughes et al. (7) |
|  | GluR1-7 |  | Rat, cat | Hamassaki-Britto et al. (13) |
|  | GluR6/7, KA2 | NR1, NR2A-C | Rat | Brandstatter et al. (14) |
| Muller cells | GluR4 |  | Rat | Peng et al. (1) |
|  | GluR4 |  | Goldfish | Vandenbranden et al.(3) |

Reference List

1. Peng YW, Blackstone CD, Huganir RL, Yau KW (1995) Distribution of glutamate receptor subtypes in the vertebrate retina. Neuroscience 66:483-497.

2. Jakobs TC, Ben Y, Masland RH (2007) Expression of mRNA for glutamate receptor subunits distinguishes the major classes of retinal neurons, but is less specific for individual cell types. Mol Vis 13:933-948.

3. Vandenbranden CA, Kamphuis W, Nunes CB, Kamermans M (2000) Expression and localization of ionotropic glutamate receptor subunits in the goldfish retina--an in situ hybridization and immunocytochemical study. J Neurocytol 29:729-742.

4. Hartveit E, Brandstatter JH, Sassoe-Pognetto M, Laurie DJ, Seeburg PH et al. (1994) Localization and developmental expression of the NMDA receptor subunit NR2A in the mammalian retina. J Comp Neurol 348:570-582.

5. Wenzel A, Benke D, Mohler H, Fritschy JM (1997) N-methyl-D-aspartate receptors containing the NR2D subunit in the retina are selectively expressed in rod bipolar cells. Neuroscience 78:1105-1112.

6. Hughes TE (1997) Are there ionotropic glutamate receptors on the rod bipolar cell of the mouse retina? Vis Neurosci 14:103-109.

7. Hughes TE, Hermans-Borgmeyer I, Heinemann S (1992) Differential expression of glutamate receptor genes (GluR1-5) in the rat retina. Vis Neurosci 8:49-55.

8. Puller C, Haverkamp S, Grunert U (2007) OFF midget bipolar cells in the retina of the marmoset, Callithrix jacchus, express AMPA receptors. J Comp Neurol 502:442-454.

9. Hanna MC, Calkins DJ (2007) Expression of genes encoding glutamate receptors and transporters in rod and cone bipolar cells of the primate retina determined by single-cell polymerase chain reaction. Mol Vis 13:2194-2208.

10. Kamphuis W, Klooster J, Dijk F (2003) Expression of AMPA-type glutamate receptor subunit (GluR2) in ON-bipolar neurons in the rat retina. J Comp Neurol 455:172-186.

11. Kamphuis W, Dijk F, O'Brien BJ (2003) Gene expression of AMPA-type glutamate receptor subunits in rod-type ON bipolar cells of rat retina. Eur J Neurosci 18:1085-1092.

12. Klooster J, Studholme KM, Yazulla S (2001) Localization of the AMPA subunit GluR2 in the outer plexiform layer of goldfish retina. J Comp Neurol 441:155-167.

13. Hamassaki-Britto DE, Hermans-Borgmeyer I, Heinemann S, Hughes TE (1993) Expression of glutamate receptor genes in the mammalian retina: the localization of GluR1 through GluR7 mRNAs. J Neurosci 13:1888-1898.

14. Brandstatter JH, Hartveit E, Sassoe-Pognetto M, Wassle H (1994) Expression of NMDA and high-affinity kainate receptor subunit mRNAs in the adult rat retina. Eur J Neurosci 6:1100-1112.
