# Supplementary material for: Effects of the AMPA Antagonist ZK 200775 on Visual Function: A Randomized Controlled Trial
Source: PLoS One. 2010 Aug 12;5(8):e12111. doi: 10.1371/journal.pone.0012111 (PMC2920815; doi:10.1371/journal.pone.0012111)
Supplement: Table S3 — Eye Motility. Data for both eyes and each group before infusion and 4 and 22 hours after infusion of ZK 200775. No significant changes occurred. (0.05 MB DOC) [file pone.0012111.s006.doc]

| **RIGHT EYE** | | **Group** | | |
| --- | --- | --- | --- | --- |
|  |  | **Low Dose (Group 1)** | **High Dose (Group 2)** | **Control group** |
| Motility of the right eye at baseline | Normal | 4 | 5 | 5 |
|  | Abnormal, no clinical significance | 2 | 1 | 1 |
|  | Abnormal, clinical significance | 0 | 0 | 0 |
| Motility of the right eye 4 hours after infusion | Normal | 4 | 4 | 5 |
|  | Abnormal, no clinical significance | 2 | 2 | 1 |
|  | Abnormal, clinical significance | 0 | 0 | 0 |
| Motility of the right eye 22 hours after infusion | Normal | 4 | 5 | 5 |
|  | Abnormal, no clinical significance | 2 | 1 | 1 |
|  | Abnormal, clinical significance | 0 | 0 | 0 |

| **LEFT EYE** | | **Group** | | |
| --- | --- | --- | --- | --- |
|  |  | **Low Dose (Group 1)** | **High Dose (Group 2)** | **Control group** |
| Motility of the left eye at baseline | Normal | 4 | 5 | 5 |
|  | Abnormal, no clinical significance | 2 | 1 | 1 |
|  | Abnormal, clinical significance | 0 | 0 | 0 |
| Motility of the left eye 4 hours after infusion | Normal | 4 | 4 | 5 |
|  | Abnormal, no clinical significance | 2 | 2 | 1 |
|  | Abnormal, clinical significance | 0 | 0 | 0 |
| Motility of the left eye 22 hours after infusion | Normal | 4 | 5 | 5 |
|  | Abnormal, no clinical significance | 2 | 1 | 1 |
|  | Abnormal, clinical significance | 0 | 0 | 0 |
