# Supplementary material for: Effects of the AMPA Antagonist ZK 200775 on Visual Function: A Randomized Controlled Trial
Source: PLoS One. 2010 Aug 12;5(8):e12111. doi: 10.1371/journal.pone.0012111 (PMC2920815; doi:10.1371/journal.pone.0012111)
Supplement: Table S5 — Amsler Card examination. Data of the left eye for each group before infusion and 4 and 22 hours after infusion of ZK 200775. No significant changes occurred. (0.04 MB DOC) [file pone.0012111.s008.doc]

|  | | | | **Group** | | |
| --- | --- | --- | --- | --- | --- | --- |
|  | | | | **Low Dose (Group 1)** | **High Dose (Group 2)** | **Control Group** |
|  | **Screening** |  | Normal | 6 | 6 | 6 |
|  |  |  | Abnormal, without clinical relevance | 0 | 0 | 0 |
|  |  |  | Abnormal, with clinical relevance | 0 | 0 | 0 |
|  | **Baseline** |  | Normal | 6 | 6 | 6 |
|  |  |  | Abnormal, without clinical relevance | 0 | 0 | 0 |
|  |  |  | Abnormal, with clinical relevance | 0 | 0 | 0 |
|  | **+4 hours** |  | Normal | 4 | 6 | 6 |
|  |  |  | Abnormal, without clinical relevance | 0 | 0 | 0 |
|  |  |  | Abnormal, with clinical relevance | 2 | 0 | 0 |
|  | **+22 hours** |  | Normal | 6 | 6 | 6 |
|  |  |  | Abnormal, without clinical relevance | 0 | 0 | 0 |
|  |  |  | Abnormal, with clinical relevance | 0 | 0 | 0 |
