# Supplementary material for: Effects of the AMPA Antagonist ZK 200775 on Visual Function: A Randomized Controlled Trial
Source: PLoS One. 2010 Aug 12;5(8):e12111. doi: 10.1371/journal.pone.0012111 (PMC2920815; doi:10.1371/journal.pone.0012111)
Supplement: Table S6 — Inclusion and exclusion criteria. (0.04 MB DOC) [file pone.0012111.s009.doc]

| **Inclusion criteria** | **Exclusion criteria** |
| --- | --- |
| - Voluntary participation - Age between 55 and 65 years - Body weight must not exceed the following value: Body height in cm minus 100 = body weight [kg] +/- 20% - Male sex - Written informed consent - Physical examination: Normal findings without clinical relevance, mental and physical health | - Clinical history   - Substantial pre-existing medical condition   - Allergy to the employed effective or galenic components |
|  | - Medicaments and drugs   - Intake of systemically or locally acting drugs which conflict with the aim of the trial or that can influence the results (antipsychotic drugs, antidepressants, barbiturates and benzodiazepines)   - A clinical history that hints to substance or alcohol abuse   - Nicotine abuse of more than 10 cigarettes a day   - Consumption of alcoholic beverages on the day prior to the examinations   - Extreme physical stress (sports- or work-related) within 8 days prior to the examinations   - Blood donation within two monts prior to the examinations   - Relevant vaccination or stay abroad   - Special or onesided alimentation (strict vegetarianism, low-caloric diet)   - Simultaneous participation in another clinical trial |
|  | - Vital signs (after 3 minutes of rest)   - Blood pressure with systolic values > 160 mmHg and / or diastolic values > 95 mmHg   - Heart frequency below 50 or above 100 beats per minute |
|  | - Electrocardiogram   - Abnormal 12-channel ECG |
|  | - Laboratory findings   - Hepatitis antigen (HbsAG), hepatitis C antibodies or positive HIV-test |
|  | - Clinical pharmacology   - Positive drug test   - Clinically relevant abnormalities of the examined parameters |
|  | - Opinion of the investigator   - When due to scientific or personal reasons or matters of compliance or safety a patient should not take part in the trial |
|  | - Ophthalmologic findings   - Opacification of the optic media, retinal disease, optic nerve disease, amblyopida or color vision defects   - Status post intraocular surgery (exception: cataract surgery with implantation of a posterior chamber lens), laser coagulation   - Myopia of more than -5 diopters, hyperopia of more than +5 diopters   - Narrow angle glaucoma |
